# Taxonomic revision and molecular phylogenetics of the *Idarnes incertus* species-group (Hymenoptera, Agaonidae, Sycophaginae)

**DOI:** 10.7717/peerj.2842

**Published:** 2017-01-05

**Authors:** Fernando H.A. Farache, Astrid Cruaud, Gwenaëlle Genson, Jean-Yves Rasplus, Rodrigo A.S. Pereira

**Affiliations:** 1 Departamento de Biologia, FFCLRP, Universidade de São Paulo, Ribeirão Preto, SP, Brazil; 2 INRA, UMR 1062 CBGP, Centre de Biologie pour la Gestion des Populations, Montferrier-sur-Lez, France

**Keywords:** Fig wasp, Ficus, Chalcidoidea, Neotropic, Gall maker, Taxonomy

## Abstract

Sycophaginae is a group of non-pollinating fig wasps considered closely related to the fig pollinators (Agaoninae, Tetrapusiinae, and Kradibiinae) in the most recent phylogenetic analyses. They occur in all tropical regions and are associated with *Ficus* subgenera *Urostigma* and *Sycomorus*. There are six described genera of Sycophaginae, and two are native and confined to the Neotropics, namely *Idarnes* Walker, 1843 and *Anidarnes* Bouček, 1993. Genus *Idarnes* is divided into three morphologically distinct groups that were proven to be monophyletic by recent molecular phylogenetic analyses. In this paper we reviewed the *Idarnes incertus* species-group and provide detailed morphological descriptions and illustrations for the species belonging to this group. Three previously described species were redescribed: *I. brasiliensis* (Mayr, 1906) **comb. nov.**, *I. hansoni* Bouček, 1993, and *I. incertus* (Ashmead, 1900). Seventeen new species are described by Farache and Rasplus: *I. amacayacuensis* **sp. n.**, *I. amazonicus* **sp. n.**, *I. americanae* **sp. n.**, *I. badiovertex* **sp. n.**, *I. brevis* **sp. n.**, *I. brunneus* **sp. n.**, *I. comptoni* **sp. n.**, *I. cremersiae* **sp. n.**, *I. dimorphicus* **sp. n.**, *I. flavicrus* **sp. n.**, *I. flaviventris* **sp. n.**, *I. gibberosus* **sp. n.**, *I. gordhi* **sp. n.**, *I. maximus* **sp. n.**, *I. nigriventris* **sp. n.**, *I. pseudoflavus* **sp. n.** and *I. ramirezi* **sp. n*.*** We provided keys for the identification of the species as well as for recognising the different species-groups of *Idarnes* and a closely related genus (*Sycophaga* Westwood, 1840). Additionally, phylogenetic relationships among 13 species of the *I. incertus* species-group were inferred using four molecular markers and discussed in the light of *Ficus* taxonomy and host specificity.

## Introduction

Fig trees (*Ficus* spp., Moraceae) host diverse assemblages of wasps that use the fig inflorescences (syconia or figs) to reproduce and develop. Fig pollinators (subfamilies Agaoninae, Tetrapusiinae, and Kradibiinae) form a very specialized clade of wasps that enter the fig trough a small pore enclosed by bracts, called ostiole. They lay eggs in the ovaries of pistilate flowers (Galil & Eisikowitch, 1969) and pollinate. Several other lineages of chalcid wasps use the fig to oviposit but do not pollinate, and are referred to as non-pollinating fig wasps (NPFW). These wasps exhibit variable life history traits (Elias, Menezes Jr & Pereira, 2008; Pereira, Teixeira & Kjellberg, 2007; Tzeng et al., 2008). They are gallers, parasitoids, cleptoparasites or even facultative or obligatory seed predators (Pereira, Teixeira & Kjellberg, 2007; Wang et al., 2014).

The Sycophaginae are NPFW that occur in all tropical regions. They are associated with *Ficus* subgenera *Urostigma* and *Sycomorus* (Cruaud et al., 2011a; Wiebes, 1966). Six genera and ca. 74 described species belong to the Sycophaginae (Cruaud et al., 2011b; Farache et al., 2013; Farache & Rasplus, 2014; Farache & Rasplus, 2015). However, the overall diversity of the Sycophaginae is estimated to ca. 700 species (Cruaud et al., 2011b).

Bouček (1988) assigned all NPFW subfamilies (namely Epichrysomallinae, Otitesellinae, Sycoecinae, Sycophaginae and Sycoryctinae), and pollinators to Agaonidae, mostly based on the morphology of the postgenal bridge. However, molecular phylogenetic analyses and a re-evaluation of the postgenal bridge morphology evidenced this grouping as non-monophyletic (Rasplus et al., 1998). Heraty et al. (2013) recovered Sycophaginae as sister to the pollinating fig wasps (Agaonidae) and proposed the inclusion of Sycophaginae in Agaonidae; most of the other fig wasp subfamilies were assigned to Pteromalidae.

Life history traits and oviposition behaviour are variable in Sycophaginae. Most species oviposit through the fig wall and induce galls in pistilate flowers. They also may oviposit in galls induced by other wasps and develop as cleptoparasites or parasitoids. Several species (a clade within *Sycophaga*) enter the fig through the ostiole as pollinators do, and induce galls in pistilate flowers (Cook & Rasplus, 2003; Cook & Segar, 2010; Cruaud et al., 2011b; Elias et al., 2012; Galil, Dulberger & Rosen, 1970).

Two genera of Sycophaginae are associated with *Ficus* in the Neotropical region, namely *Anidarnes* Bouček, 1993 and *Idarnes* Walker, 1843 (Bouček, 1993; Rasplus & Soldati, 2005). They are strictly associated with *Ficus* section *Americanae*. *Idarnes* is the most diverse NPFW genus in the Neotropics. Twenty-three species are recognised as belonging to *Idarnes* (Bouček, 1993; Cruaud et al., 2011b; Gordh, 1975), but the overall diversity of the genus is estimated to nearly 300 species (Cruaud et al., 2011b). Some Old-World species were classified under *Idarnes*; however, they all belong to *Sycophaga* Westwood, 1840 (= *Apocryptophagus* Ashmead, 1904) (Bouček, 1993; Cruaud et al., 2011b; Gordh, 1975) and consequently, *Idarnes* is restricted to the Neotropics. Concerning nomenclature, *Idarnes* should be treated as masculine as well as *Anidarnes* and other derived names (Farache et al., 2013). The name probably refers to an eminent Persian commander, *Hydarnes*, who was given command of the “Immortals” and fought the Greeks in the battle of Thermopylae, 480 BC.

Three morphological species-groups of *Idarnes* are recognised, namely *I. carme*, *I. flavicollis* and *I. incertus* species-groups. They exhibit clear morphological differences (Bouček, 1993) and contrasted life history traits. Species belonging to the *I. incertus* species-group are gall-makers and oviposit before pollination. *Idarnes flavicollis* species-group species are also gall-makers but oviposit at the same time as pollinators. The species belonging to the *I. carme* species-group oviposit after pollination and are probably cleptoparasites associated with pollinator’s larvae (Elias, Menezes Jr & Pereira, 2008; Elias et al., 2012).

The purpose of this paper is to provide a taxonomic revision of the *Idarnes incertus* species-group. Three species are re-described and 17 species are described from samples collected in Brazil, Colombia, Costa Rica and French Guiana. All species are illustrated and an identification key is provided. Phylogenetic relationships including 13 species of *Idarnes incertus* species-group and eight outgroups were inferred using multiple genes, and their relationships were discussed in the light of the taxonomy of their hosts.

## Materials & Methods

### Specimen collection and morphological study

Figs were sampled before maturity and transferred to tissue bags until wasp emergence. Wasps were killed using ethyl acetate or freezing and stored in 70% ethanol. Geographical coordinates and altitude were recorded in the field using a GPS device or estimated using label information. ICMBio provided permissions for material sampling to RASP in Brazil (Permit #1870297).

Specimens were dehydrated through an ethanol and HMDS series (Heraty & Hawks, 1998) or critical point dried (Gordh & Hall, 1979) using BALTEC CPD 030′. Insects were card-mounted following Noyes (1982). Morphological terminology follows Gibson (1997). Measurements were taken using Leica application suite V3.6. Abbreviations for measurements used in the text include: POL = distance between lateral ocelli; OOL = distance between one posterior ocellus and adjacent composite eye.

Multi-entry online keys were produced using Lucid^®^ v. 3.3. They are available at figweb (http://www.figweb.org—Van Noort & Rasplus, 2016) and as Supplemental Information 1.

The electronic version of this article in Portable Document Format (PDF) will represent a published work according to the International Commission on Zoological Nomenclature (ICZN), and hence the new names contained in the electronic version are effectively published under that Code from the electronic edition alone. This published work and the nomenclatural acts it contains have been registered in ZooBank, the online registration system for the ICZN. The ZooBank LSIDs (Life Science Identifiers) can be resolved and the associated information viewed through any standard web browser by appending the LSID to the prefix http://zoobank.org/. The LSID for this publication is: *urn:lsid:zoobank.org:pub:22286699-8306-4931-8D7F-7BF05EB2B304*. The online version of this work is archived and available from the following digital repositories: PeerJ, PubMed Central and CLOCKSS.

*Acronyms for repositories* follow Arnett, Samuelson & Nishida (1993) when available:

**Table:** 

**BMNH:** The Natural History Museum, London, U.K.
**CBGP:** Centre de Biologie pour la Gestion des Populations, Montferrier-sur-Lez, France.
**EBCR:** Escuela de Biologia, Universidad de Costa Rica, San José, Costa Rica.
**MZSP:** Museu de Zoologia da Universidade de São Paulo, São Paulo, Brazil.
**NMW:** Naturhistorisches Museum, Wien, Austria.
**SAMC:** Iziko South African Museum, Cape Town, South Africa.
**RPSP:** Universidade de São Paulo, Ribeirão Preto, Brazil.
**USNM:** National Museum of Natural History, Washington D.C., U.S.A.

*Illustrations.* Images of specimens were produced with a Leica MZ16 stereoscope connected to a digital camera and a computer workstation running *Leica Application Suite* (LAS) V3.6 imaging software. Image series comprising about 15–20 focal planes were merged to produce a single image with increased depth of field.

Some specimens were dissected, mounted and sputter-coated with gold for scanning electron microscopy (SEM), which was performed with a Zeiss EVO 50 microscope. SEM images of species with few specimens were obtained with a low vacuum protocol.

**Figure 1:**
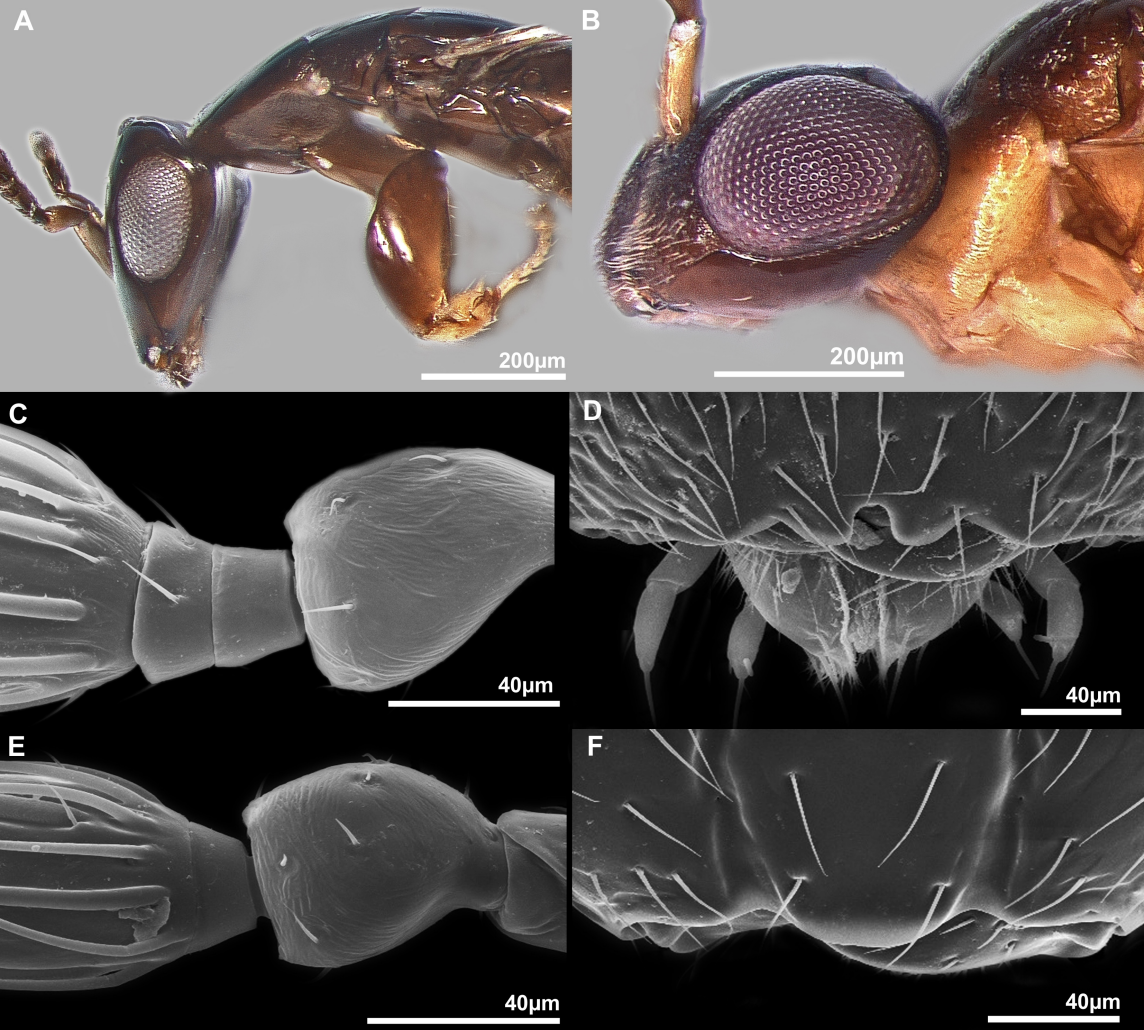
Sycophaginae morphology. (A) *Sycophaga sycomori*, lateral view of head and mesosoma; (B) *Sycophaga testacea*, lateral view of head; (C) *Idarnes flavicollis* sp. group, detail of antenna; (D) *Idarnes flavicollis* sp. group, detail of clypeus; (E) *Idarnes carme* sp. group, detail of antenna; (F) *Idarnes carme* sp. group, detail of clypeus.

**Figure 2:**
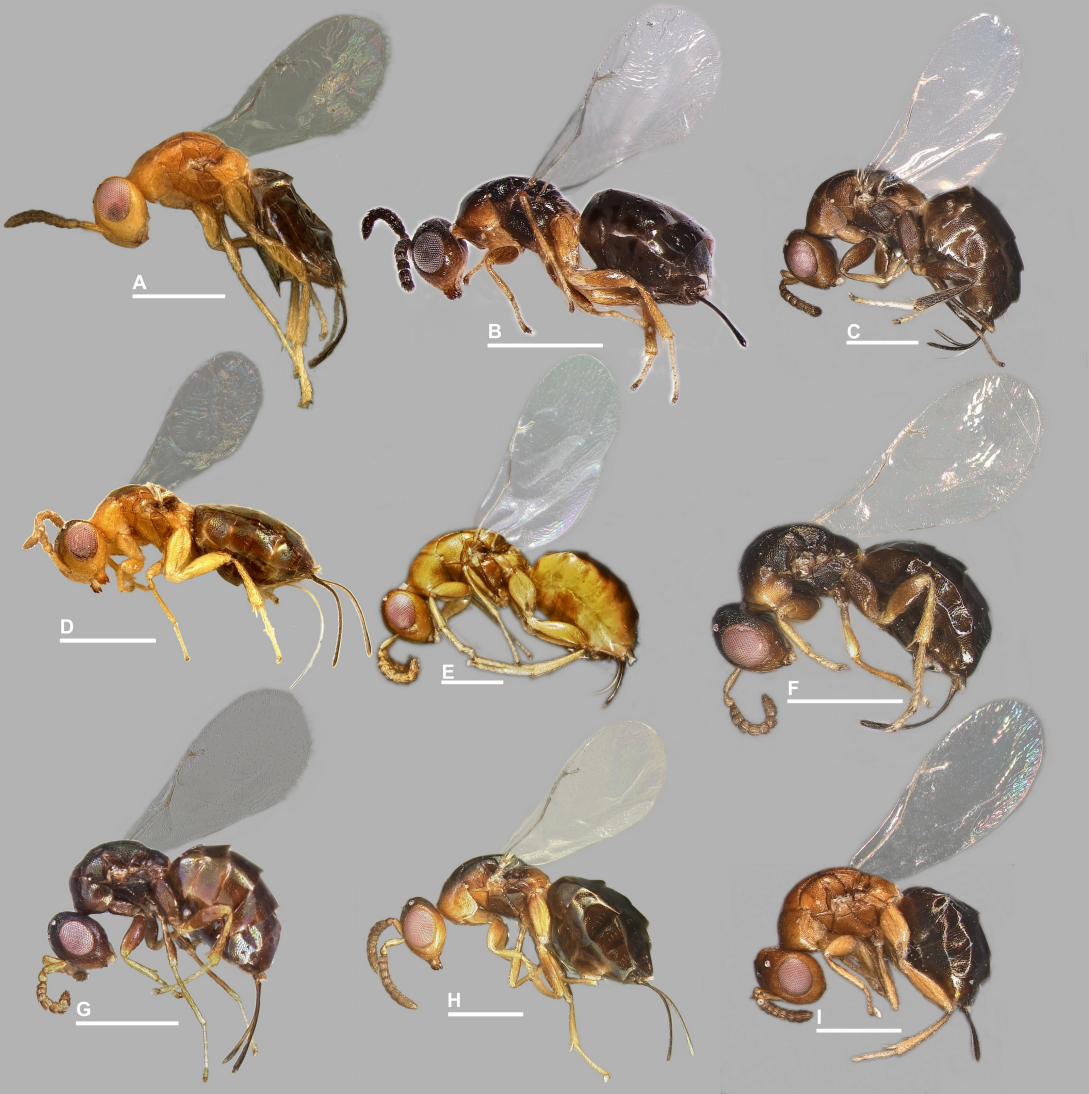
*Habitus* in lateral view, *Idarnes incertus* sp. group, females. (A) *I. amacayacuensis* sp. n.; (B) *I. amazonicus* sp. n.; (C) *I. americanae* sp. n.; (D) *I. badiovertex* sp. n.; (E) *I. brevis* sp. n.; (F) *I. brunneus* sp. n.; (G) *I. comptoni* sp. n.; (H) *I. cremersiae* sp. n.; (I) *I. dimorphicus* sp. n. Scale = 500 µm.

**Figure 3:**
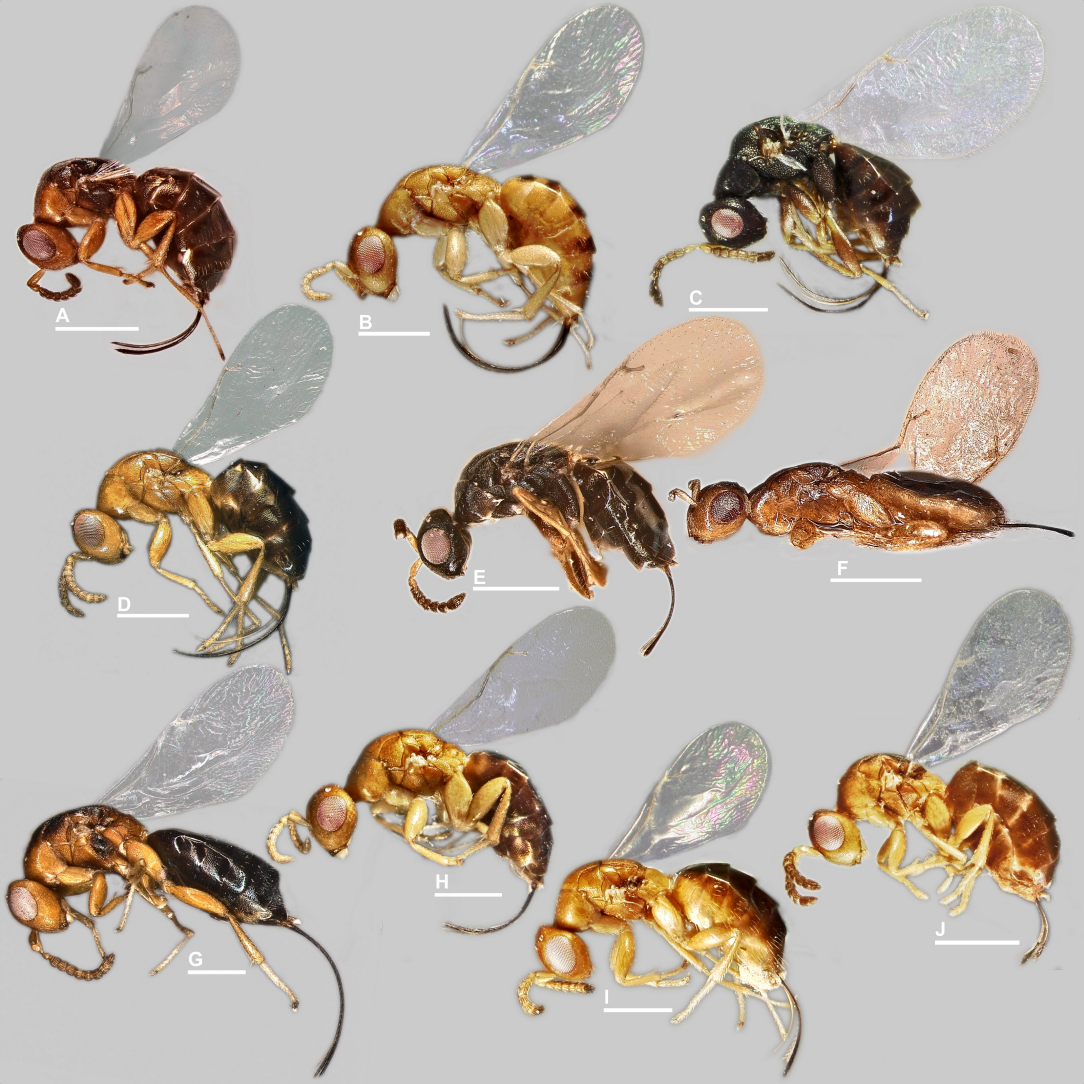
*Habitus* in lateral view, *Idarnes incertus* sp. group, females. (A) *I. flavicrus* sp. n.; (B) *I. flaviventris* sp. n.; (C) *I. gibberosus* sp. n.; (D) *I. gordhi* sp. n.; (E) *I. hansoni* Bouček, 1993, Paratype; (F) *I. incertus* (Ashmead, 1900), Paralectotype USNM; (G) *I. maximus* sp. n.; (H) *I. nigriventris* sp. n.; (I) *I. pseudoflavus* sp. n.; (J) *I. ramirezi* sp. n. Scale = 500 µm.

**Figure 4:**
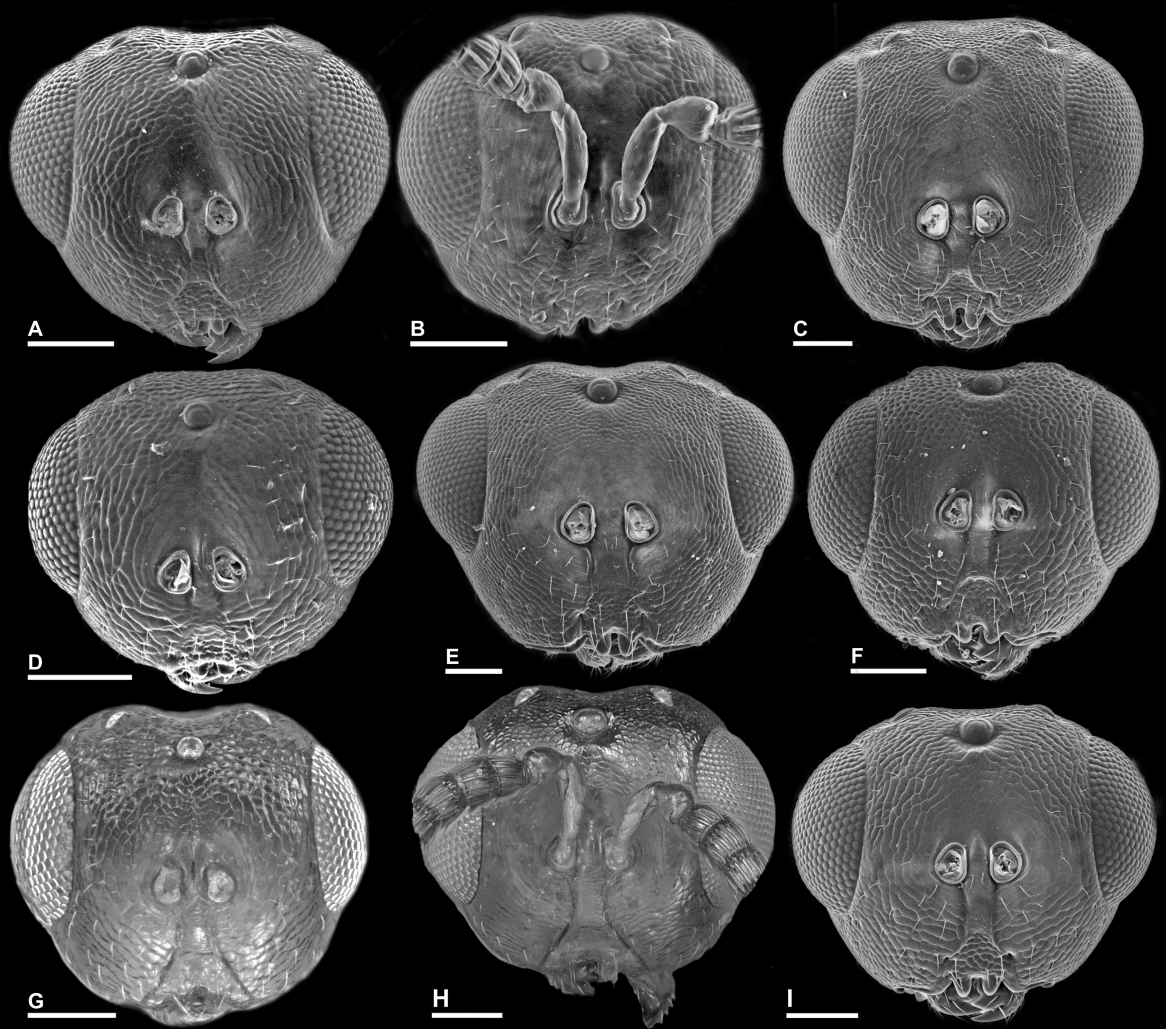
Head in frontal view, *Idarnes incertus* sp. group, females. (A) *I. amacayacuensis* sp. n.; (B) *I. amazonicus* sp. n.; (C) *I. americanae* sp. n.; (D) *I. badiovertex* sp. n.; (E) *I. brevis* sp. n.; (F) *I. brunneus* sp. n.; (G) *I. comptoni* sp. n.; (H) *I. cremersiae* sp. n.; (I) *I. dimorphicus* sp. n. Scale = 100 µm.

Pictures include details comparing *Sycophaga* and *Idarnes* species groups (Fig. 1). Images of species belonging to *Idarnes incertus* species-group include: habitus in lateral view (Figs. 2 and 3), head in frontal view (Figs. 4 and 5), antenna (Figs. 6 and 7), head and part of mesosoma (Figs. 8 and 9), mesosoma (Figs. 10 and 11), SEM of mesosoma (Figs. 12 and 13) and wing venation (Figs. 14 and 15).

**Figure 5:**
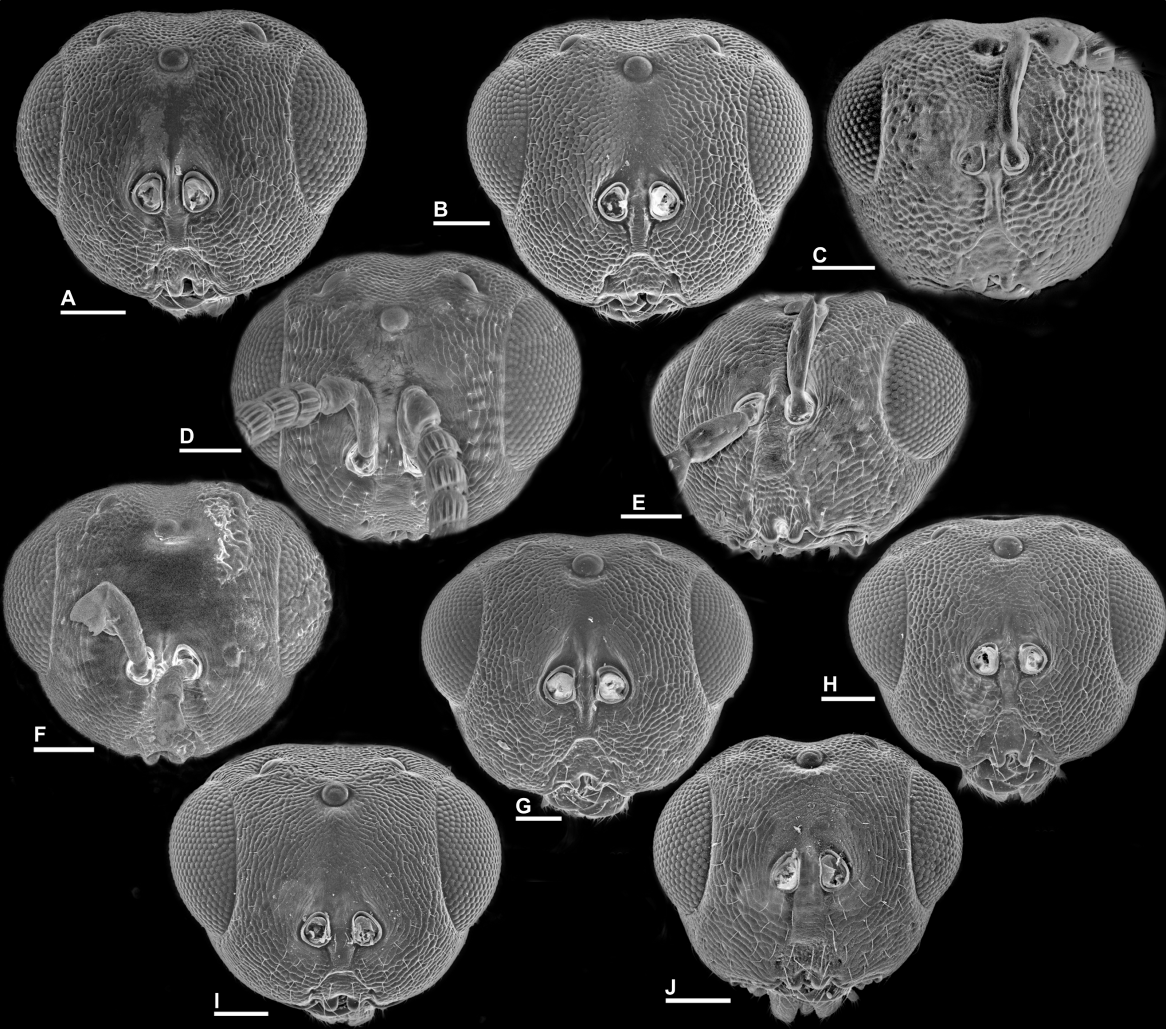
Head in frontal view, *Idarnes incertus* sp. group, females. (A) *I. flavicrus* sp. n.; (B) *I. flaviventris* sp. n.; (C) *I. gibberosus* sp. n.; (D) *I. gordhi* sp. n.; (E) *I. hansoni* Bouček, 1993, Paratype; (F) *I. incertus* (Ashmead, 1900), Paralectotype USNM; (G) *I. maximus* sp. n.; (H) *I. nigriventris* sp. n.; (I) *I. pseudoflavus* sp. n.; (J) *I. ramirezi* sp. n. Scale = 100 µm.

**Figure 6:**
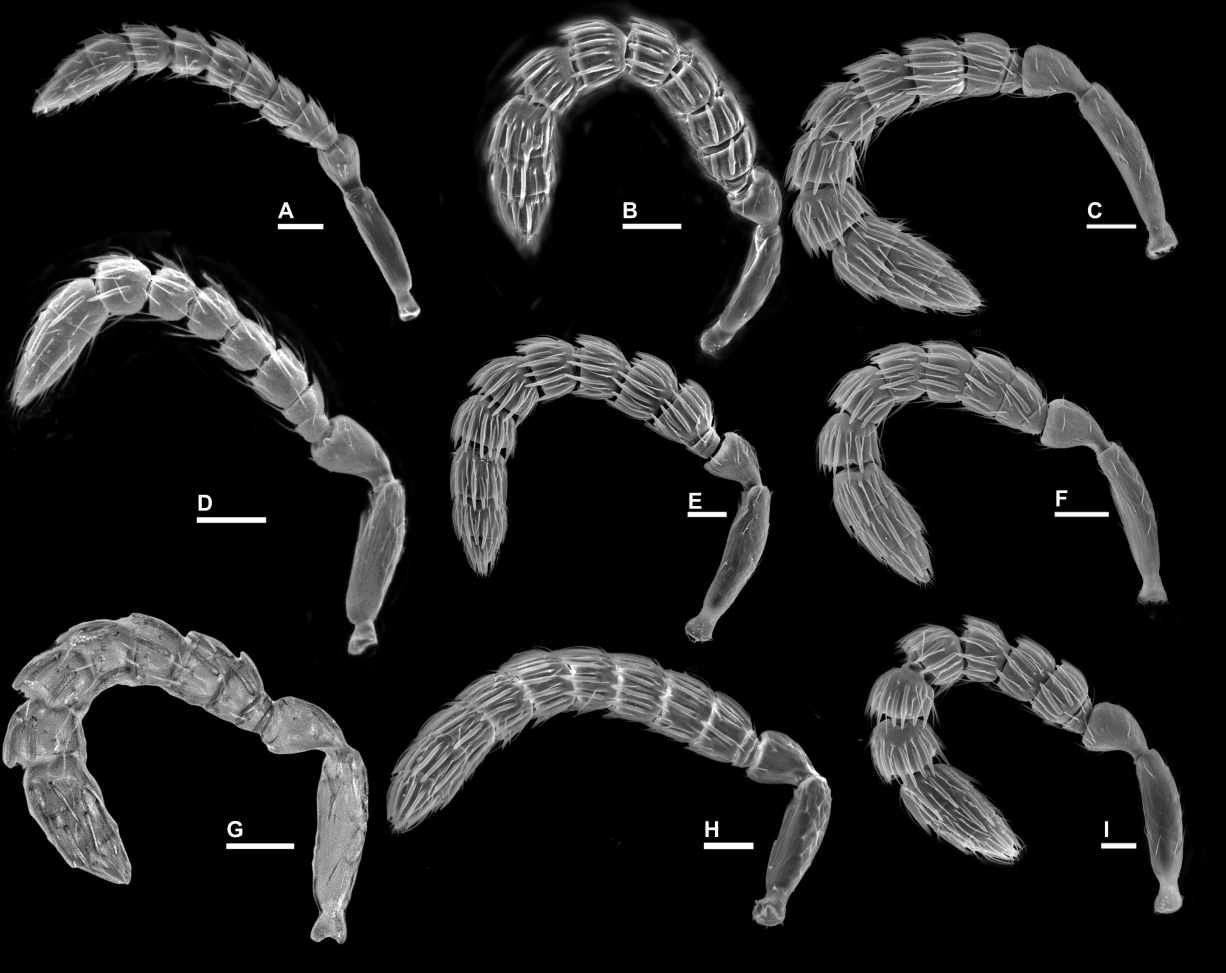
Antenna, *Idarnes incertus* sp. group, females. (A) *I. amacayacuensis* sp. n.; (B) *I. amazonicus* sp. n.; (C) *I. americanae* sp. n.; (D) *I. badiovertex* sp. n.; (E) *I. brevis* sp. n.; (F) *I. brunneus* sp. n.; (G) *I. comptoni* sp. n.; (H) *I. cremersiae* sp. n.; (I) *I. dimorphicus* sp. n. Scale = 50 µm.

**Figure 7:**
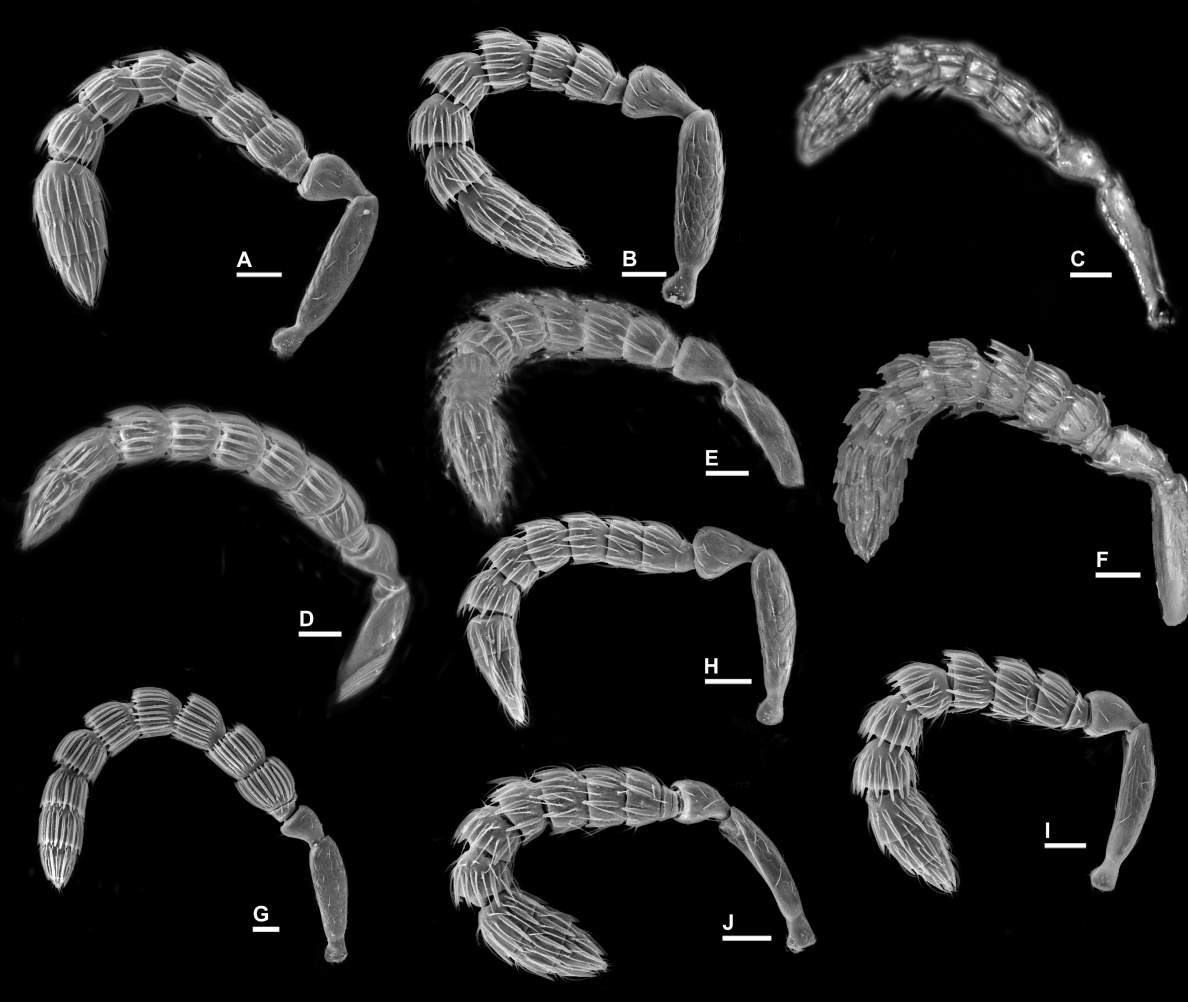
Antenna, *Idarnes incertus* sp. group, females. (A) *I. flavicrus* sp. n.; (B) *I. flaviventris* sp. n.; (C) *I. gibberosus* sp. n.; (D) *I. gordhi* sp. n.; (E) *I. hansoni* Bouček, 1993, Paratype; (F) *I. aff. incertus* (Ashmead, 1900) (JRAS01219), Paralectotype; (G) *I. maximus* sp. n.; (H) *I. nigriventris* sp. n.; (I) *I. pseudoflavus* sp. n.; (J) *I. ramirezi* sp. n. Scale = 50 µm.

**Figure 8:**
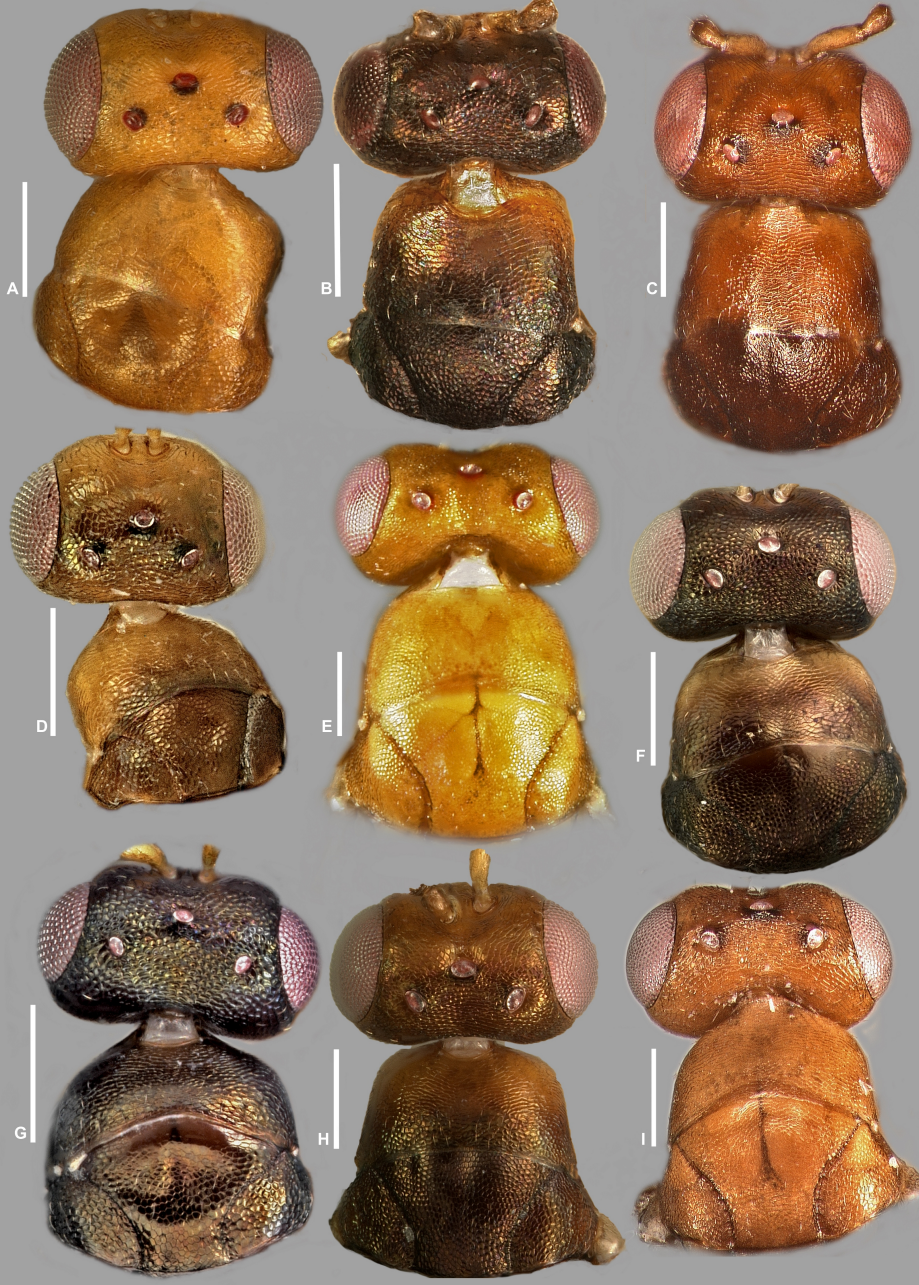
Head and mesosoma (part), *Idarnes incertus* sp. group, females. (A) *I. amacayacuensis* sp. n.; (B) *I. amazonicus* sp. n.; (C) *I. americanae* sp. n.; (D) *I. badiovertex* sp. n.; (E) *I. brevis* sp. n.; (F) *I. brunneus* sp. n.; (G) *I. comptoni* sp. n.; (H) *I. cremersiae* sp. n.; (I) *I. dimorphicus* sp. n. Scale = 200 µm.

**Figure 9:**
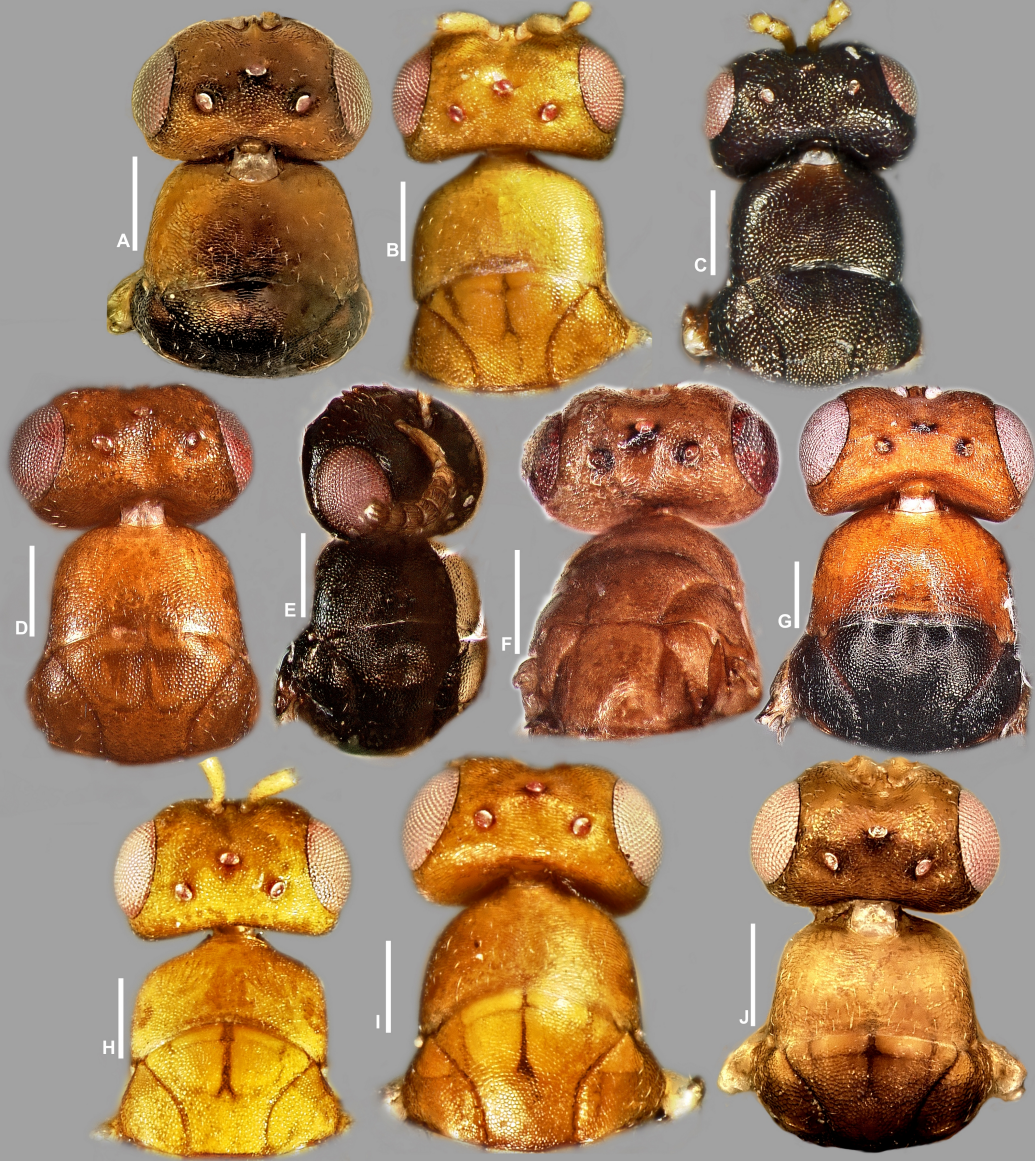
Head and mesosoma (part), *Idarnes incertus* sp. group, females. (A) *I. flavicrus* sp. n.; (B) *I. flaviventris* sp. n.; (C) *I. gibberosus* sp. n.; (D) *I. gordhi* sp. n.; (E) *I. hansoni* Bouček, 1993, Paratype; (F) *I. incertus* (Ashmead, 1900), Paralectotype USNM; (G) *I. maximus* sp. n.; (H) *I. nigriventris* sp. n.; (I) *I. pseudoflavus* sp. n.; (J) *I. ramirezi* sp. n. Scale = 200 µm.

**Figure 10:**
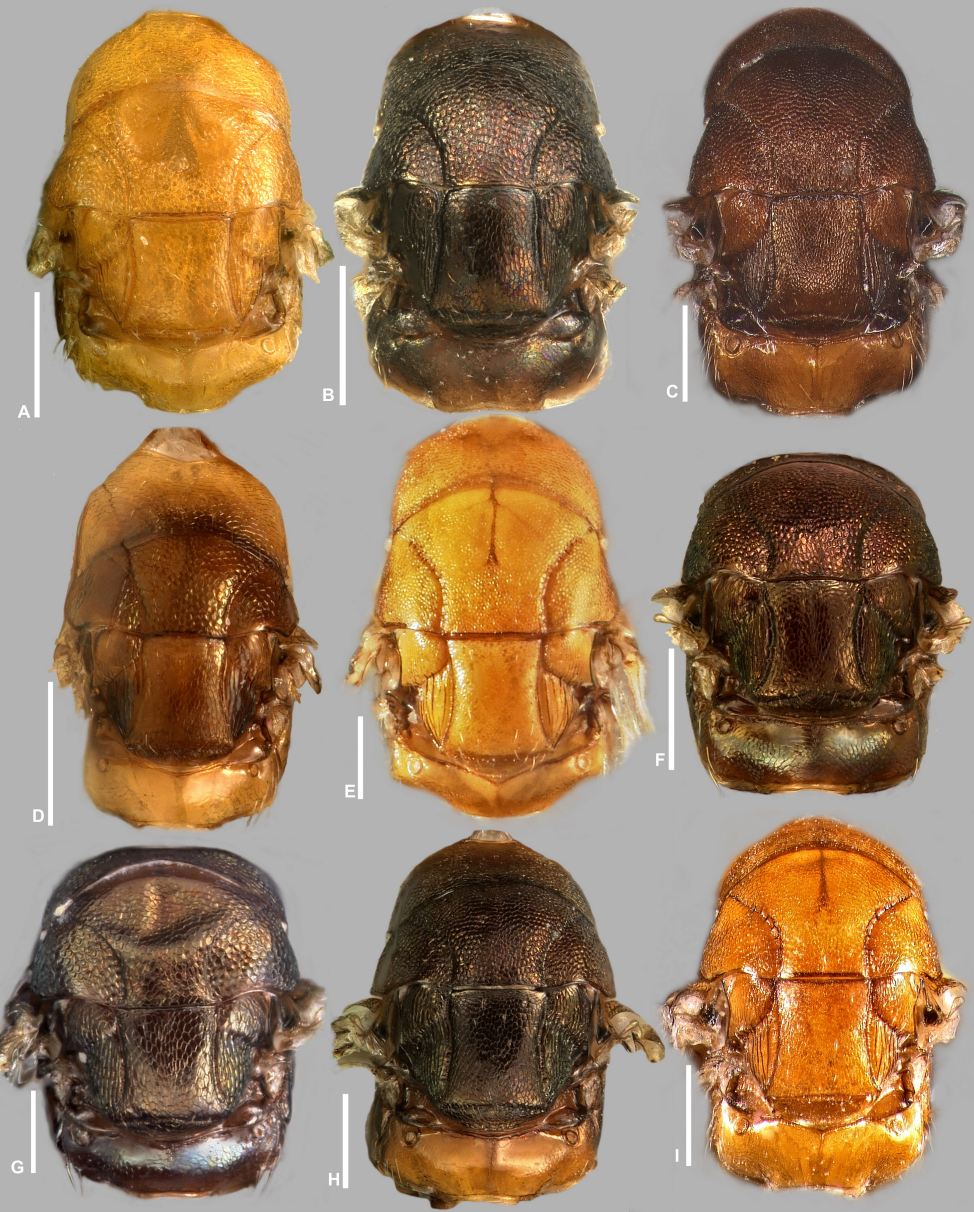
Mesosoma in dorsal view, *Idarnes incertus* sp. group, females. (A) *I. amacayacuensis* sp. n.; (B) *I. amazonicus* sp. n.; (C) *I. americanae* sp. n.; (D) *I. badiovertex* sp. n.; (E) *I. brevis* sp. n.; (F) *I. brunneus* sp. n.; (G) *I. comptoni* sp. n.; (H) *I. cremersiae* sp. n.; (I) *I. dimorphicus* sp. n. Scale = 200 µm.

**Figure 11:**
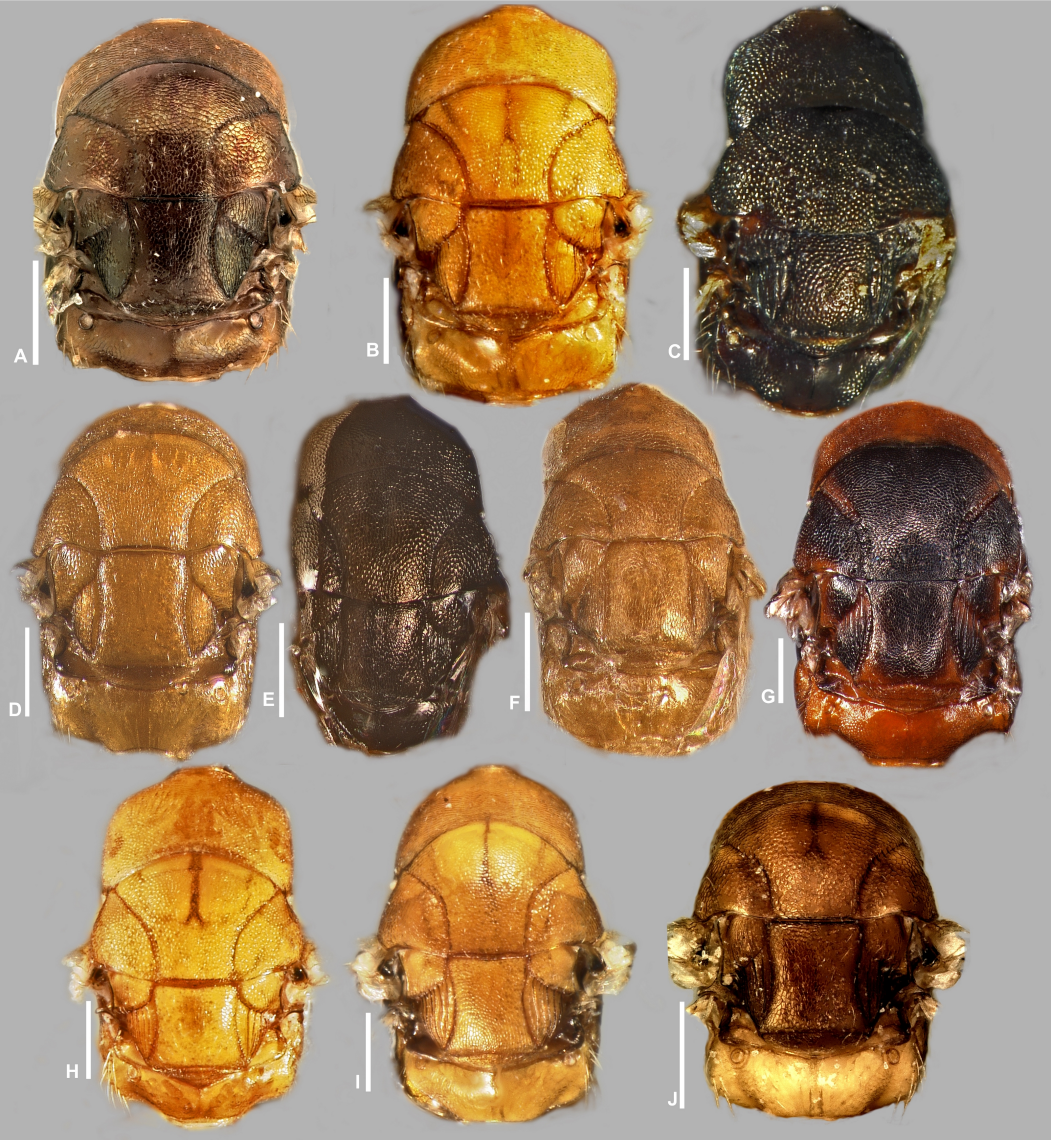
Mesosoma in dorsal view, *Idarnes incertus* sp. group, females. (A) *I. flavicrus* sp. n.; (B) *I. flaviventris* sp. n.; (C) *I. gibberosus* sp. n.; (D) *I. gordhi* sp. n.; (E) *I. hansoni* Bouček, 1993, Paratype; (F) *I. incertus* (Ashmead, 1900), Paralectotype USNM; (G) *I. maximus* sp. n.; (H) *I. nigriventris* sp. n.; (I) *I. pseudoflavus* sp. n.; (J) *I. ramirezi* sp. n. Scale = 200 µm.

**Figure 12:**
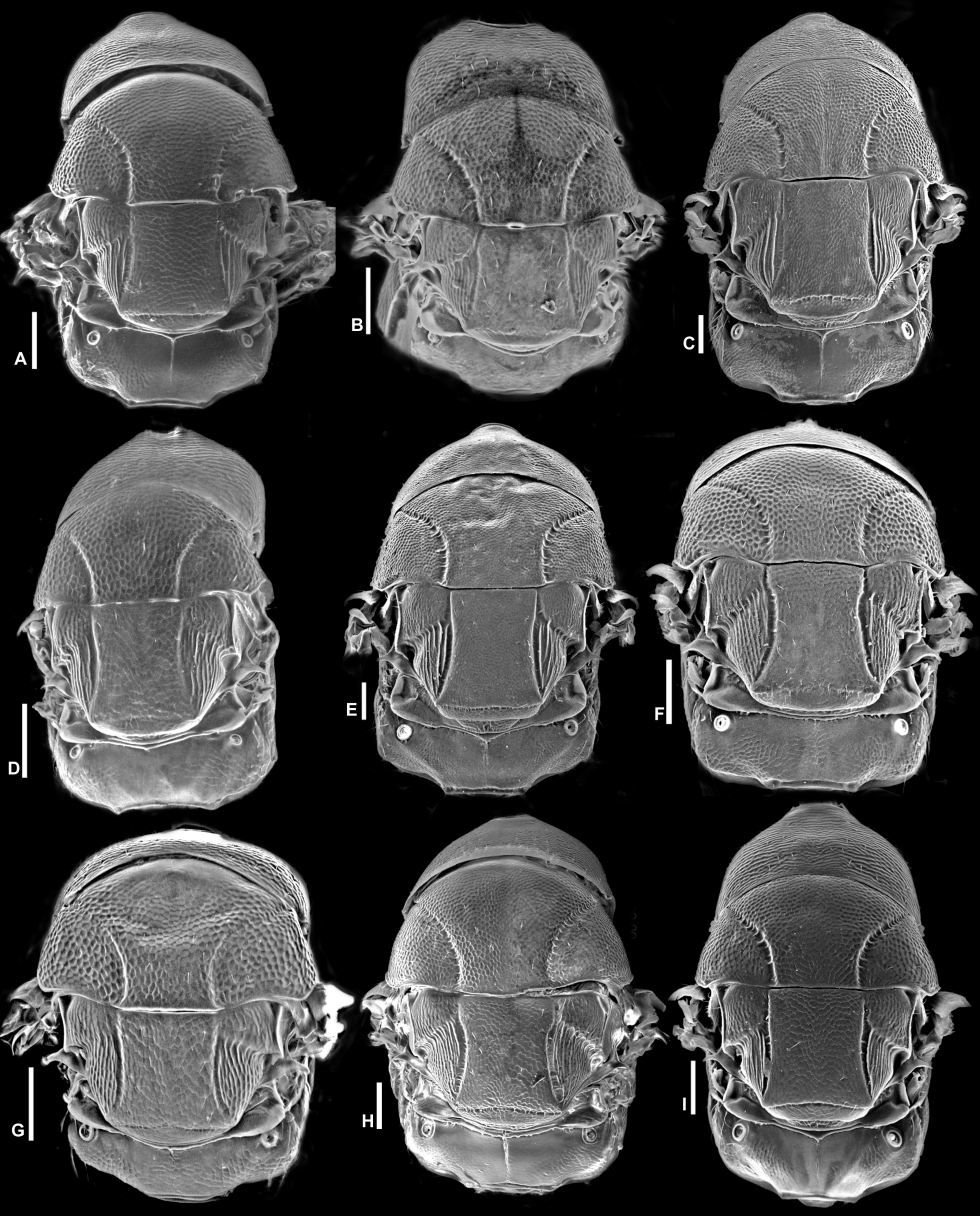
Mesosoma in dorsal view (SEM), *Idarnes incertus* sp. group, females. (A) *I. amacayacuensis* sp. n.; (B) *I. amazonicus* sp. n.; (C) *I. americanae* sp. n.; (D) *I. badiovertex* sp. n.; (E) *I. brevis* sp. n.; (F) *I. brunneus* sp. n.; (G) *I. comptoni* sp. n.; (H) *I. cremersiae* sp. n.; (I) *I. dimorphicus* sp. n. Scale = 100 µm.

**Figure 13:**
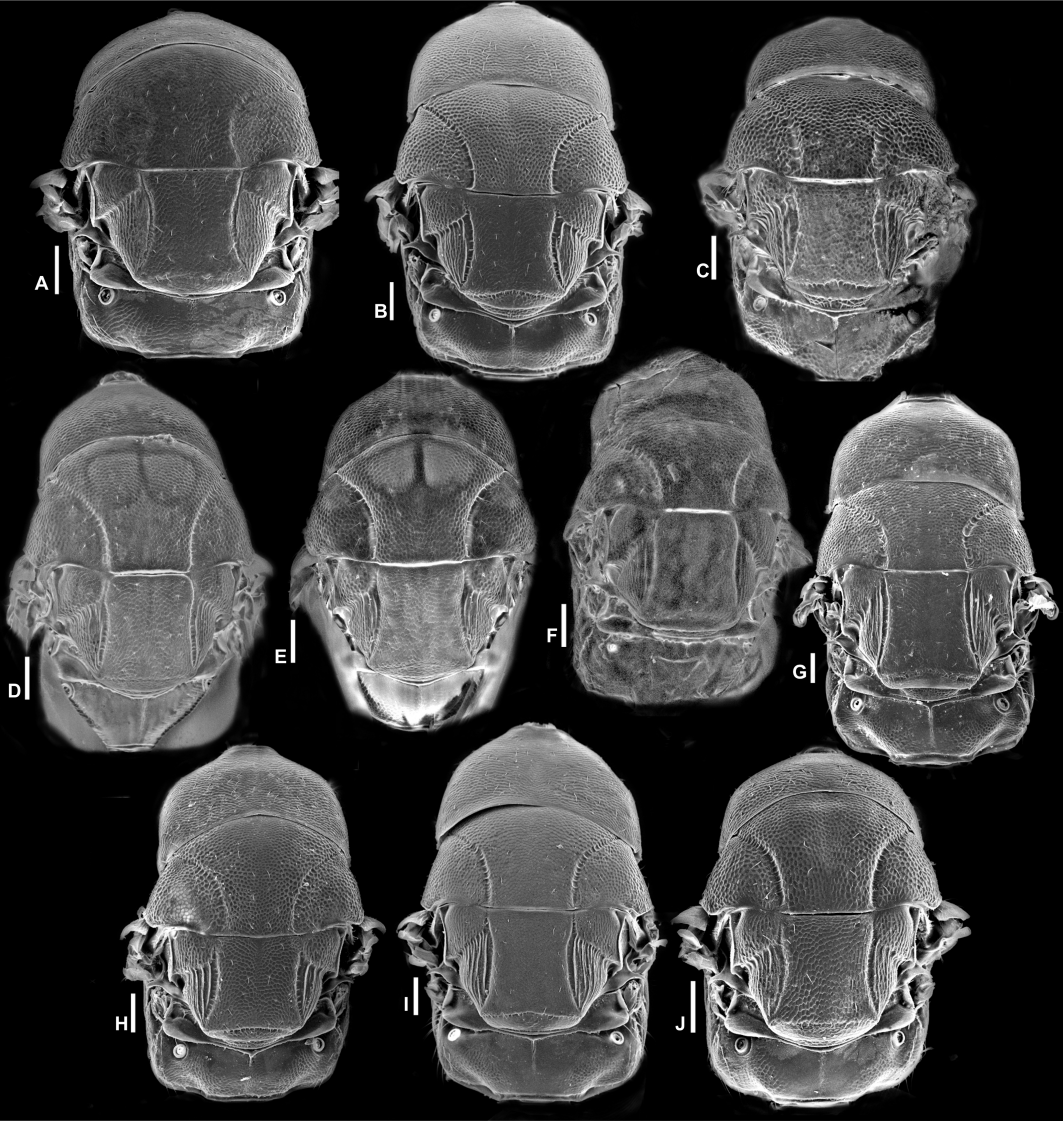
Mesosoma in dorsal view (SEM), *Idarnes incertus* sp. group, females. (A) *I. flavicrus* sp. n.; (B) *I. flaviventris* sp. n.; (C) *I. gibberosus* sp. n.; (D) *I. gordhi* sp. n.; (E) *I. hansoni* Bouček, 1993, Paratype; (F) *I. incertus* (Ashmead, 1900), Paralectotype USNM; (G) *I. maximus* sp. n.; (H) *I. nigriventris* sp. n.; (I) *I. pseudoflavus* sp. n.; (J) *I. ramirezi* sp. n. Scale = 100 µm.

**Figure 14:**
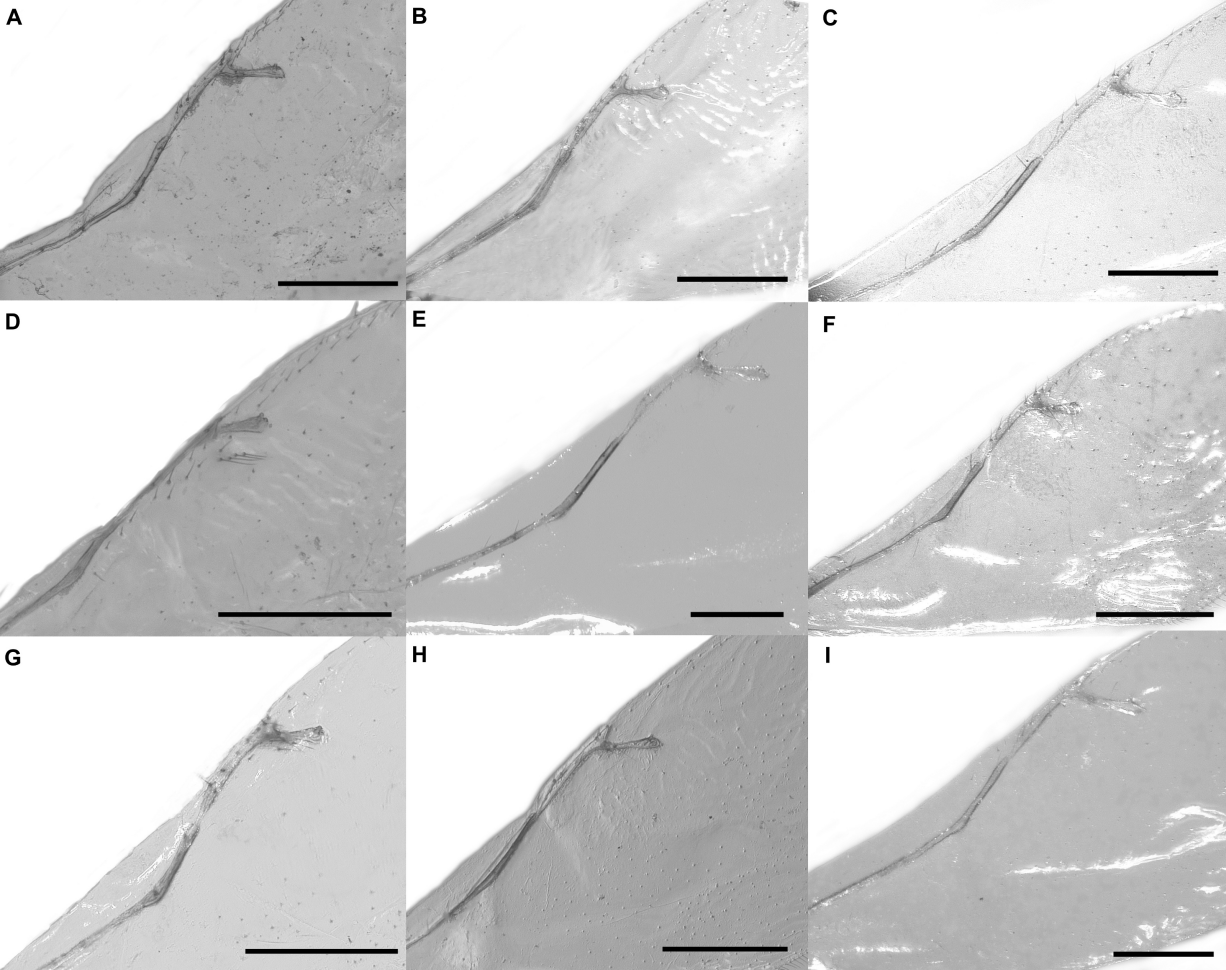
Wing venation, *Idarnes incertus* sp. group, females. (A) *I. amacayacuensis* sp. n.; (B) *I. amazonicus* sp. n.; (C) *I. americanae* sp. n.; (D) *I. badiovertex* sp. n.; (E) *I. brevis* sp. n.; (F) *I. brunneus* sp. n.; (G) *I. comptoni* sp. n.; (H) *I. cremersiae* sp. n.; (I) *I. dimorphicus* sp. n. Scale = 200 µm.

**Figure 15:**
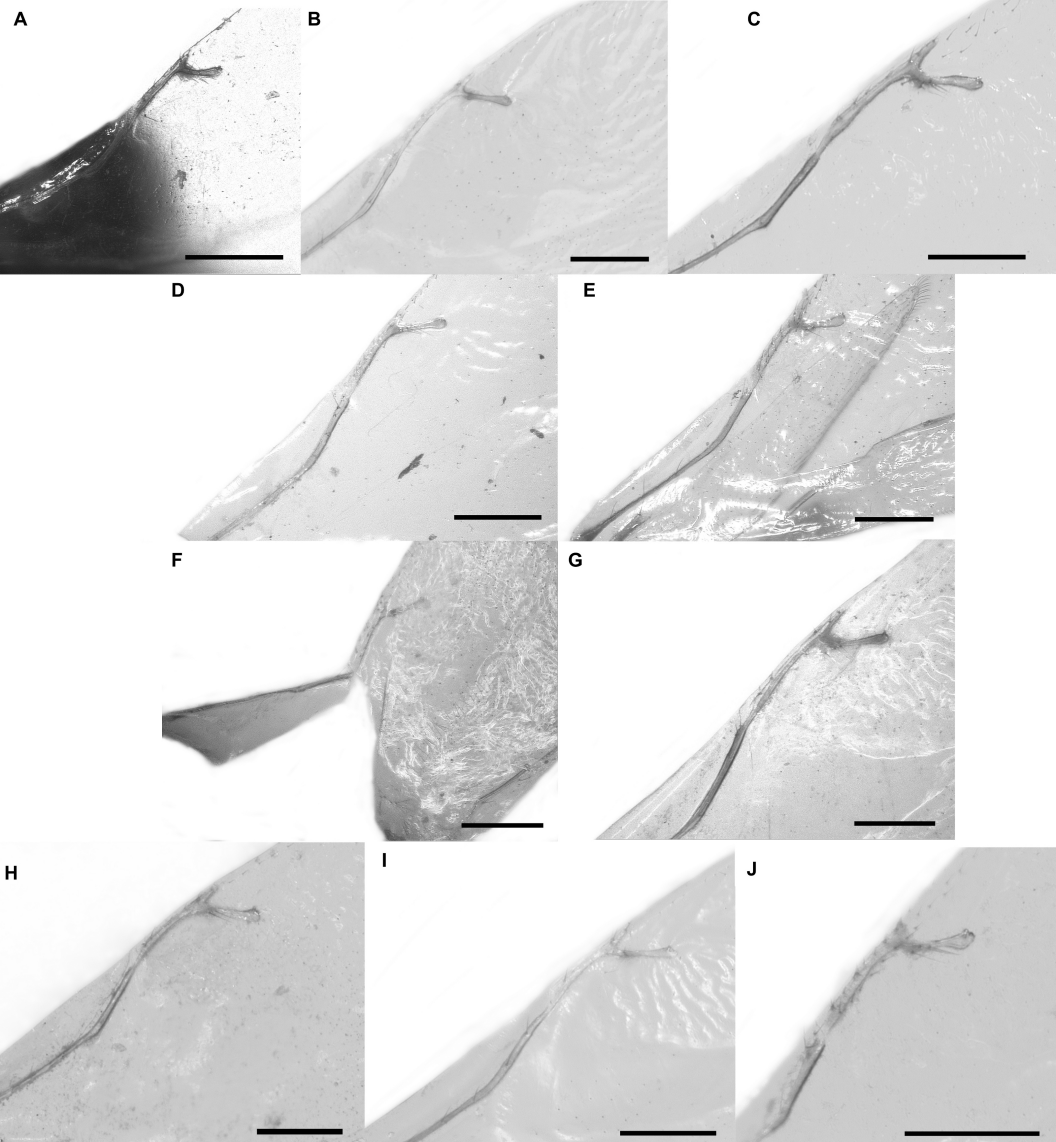
Wing venation, *Idarnes incertus* sp. group, females. (A) *I. flavicrus* sp. n.; (B) *I. flaviventris* sp. n.; (C) *I. gibberosus* sp. n.; (D) *I. gordhi* sp. n.; (E) *I. hansoni* Bouček, 1993, Paratype; (F) *I. incertus* (Ashmead, 1900), Paralectotype USNM; (G) *I. maximus* sp. n.; (H) *I. nigriventris* sp. n.; (I) *I. pseudoflavus* sp. n.; (J) *I. ramirezi* sp. n. Scale = 200 µm.

### Molecular protocols and phylogenetic analyses

In this study, we amplified one nuclear protein-coding region (F2 copy of elongation factor-1*α*, *EF-1α*), two mitochondrial protein-coding regions (Cytochrome Oxydase I—*COI* and Cytochrome B—*CytB*), and two regions of the *rRNA 28S* (D2–D3 and D4–D5 expansion regions). DNA extraction, PCR conditions, and sequencing protocols follow Cruaud et al. (2010) and Cruaud et al. (2011a). Forward and reverse strands for each fragment were assembled using the software Geneious v.6.1.8. All the sequences were deposited in GenBank (accession numbers in Supplemental Information 2). Our dataset consisted of 33 terminals, comprehending 25 specimens for 13 ingroup species belonging to the *Idarnes incertus* species-group and eight outgroup species, representing all other *Idarnes* species-groups, all known Sycophaginae genera, and an Epichrysomallinae genus (Pteromalidae).

Sequence alignment for all markers was performed in MAFFT v. 7 (Katoh & Standley, 2013) using the L-INS-i algorithm, and visually inspected. In protein coding genes, we checked protein translations to detect frameshift mutations and premature stop codons using MEGA 4 (Kumar et al., 2008). The most appropriate model of sequence evolution for each data subset most likely to have experienced similar evolutionary processes (*mitochondrial genes, EF-1α*, *rRNA 28S*) was identified using Akaike information criterion (Akaike, 1973) as implemented in jModeltest v. 2.1.7 (Darriba et al., 2012; Guindon & Gascuel, 2003). Since we used multiple loci to infer phylogenetic relationships, we established different partitions for each locus included in the analyses, allowing parameters to vary among partitions.

Phylogenetic analyses were performed using maximum likelihood (ML) and Bayesian methods, conducted in the CIPRES Science Gateway (Miller, Pfeiffer & Schwartz, 2010).

Partitioned ML analyses were performed using RAxML v 8 (Stamatakis, 2014), and the GTRCAT approximation was used for performing associated bootstrapping (1,000 replicates). Bootstrap percentage (ML_BP_) > 95% was considered as strong support and a ML_BP_ < 70% as weak.

Bayesian phylogenetic analyses were conducted using MrBayes v. 3.2.2 (Ronquist et al., 2012). We assumed across-partition heterogeneity in model parameters by considering the parameter m. Parameter values were initiated with default priors; branch lengths were estimated using default exponential priors. The optimization of the posterior probability was achieved using Metropolis-coupled Markov chain Monte Carlo (MCMC). To improve mixing of the cold chain and avoid converging on local optima, we executed two independent runs including a cold chain and three incrementally heated chains for each run. The heating parameter was set to 0.02 in order to allow more frequent swapping between cold and heated chains. The runs were executed for 10 million generations, and values were sampled every 1,000 generations. A NEXUS file including gene alignment and MrBayes block is included as Supplemental Information 3. We also ensure the convergence between parameters from the two chains by analysing estimates and frequency distributions of each parameter using Tracer v. 1.5 (Rambaut et al., 2013). We examined the plot of overall model likelihood against generation number to find the point where the likelihood started to fluctuate around a constant value, and applied a 10% relative burn-in. The results were based on the pooled samples from the stationary phases of the two independent runs. Posterior probabilities (PP) >0.95 were considered as strong support.

## Results

### Morphological definition; key to genera and species-groups

*Idarnes* is the sister group of *Sycophaga*, a diversified Old World genus mostly associated with *Ficus* subgenus *Sycomorus*, but two species are associated with *F.* subg. *Urostigma*. *Idarnes carme* species-group is sister to a clade grouping *I. flavicollis* species-group and *I. incertus* species-group (Cruaud et al., 2011a; Cruaud et al., 2011b).

*Sycophaga* and all *Idarnes* species-groups can be identified using the following key:

1 Body smooth, sculpturation shallow. Notaulus, axillula, frenal sulcus and other sutures without obvious crenulation. Head flattened dorsoventrally (Fig. 1A). Oviposits internally in figs................................................................................................. ............................................. Old World, ***Sycophaga*** **(part)**
— Body sculpture at least slightly reticulate. Notaulus, axillula, frenal sulcus and other sutures at least slightly crenulated. Head globose or subglobose in lateral view (Fig. 1B). Oviposits through the fig wall .........................................................................**2**
2 Malar sulcus present (Fig. 1B). Antenna with two anelli **and** postmarginal vein longer than stigmal vein........................................................................................................ ............................Old World, ***Sycophaga*** (**part**, formerly ***Apocryptophagus***)
— Malar sulcus absent. Antenna with one anellus **or**, if two anelli (*I. flavicollis* and *I. incertus* species-groups) postmarginal vein shorter than stigmal vein (only exception known is the fossil species *Idarnes thanatos* Farache et al., 2016)......................... .....................................................................................New World, ***Idarnes*.** **3**
3 Body mostly without metallic tinge (Figs. 2 and 3). Ovipositor sheaths shorter than body length. Funicular segments transverse......................***I. incertus*** **species-group**
— Body with metallic tinge, ovipositor as long as body or longer. Funicular segments nearly as long than wide or longer than wide............................................................ **4**
4 Postmarginal vein shorter than stigmal vein. Head sculpture homogeneous. Antenna with 2 anelli (Fig. 1C). Mandibles tridentate. Clypeal margin bilobed (Fig. 1D) (trilobed in *I. micheneri* Gordh, 1975)...................................................................................... ............................................................................... ***I. flavicollis*** **species-group**
— Postmarginal vein longer than stigmal. Head sculpture stronger near vertex Antenna with one anellus (Fig. 1E). Mandibles bidentate. Clypeal margin usually straight or unilobed (excepted one undescribed species) (Fig. 1F).................................................... ***I. carme*** **species-group**

The *I. incertus* species-group shares similarities with *Anidarnes* Bouček, 1993—another neotropical Sycophaginae genus—but can be distinguished by the following characters: (1) antennae usually inserted closer to the clypeal margin than to the median ocellus, or at most at the same distance, whereas in *Anidarnes* they are inserted closer to the median ocellus; (2) ovipositor without the median constriction apomorphic of *Anidarnes* (to the exception of *A. dissidens* Farache & Rasplus 2013); (3) metascutellum at least 3× as wide as long in *incertus* species-group whereas at most 2× as wide as long, or trapezoidal, in *Anidarnes* (sometimes the metascutellum is inconspicuous in both groups). Keys to the genera of neotropical fig wasps are provided by Bouček (1993) and Rasplus & Soldati (2005).

***Idarnes incertus*** **species-group**

1993 Bouček, Z., *Journal of Natural History* 27: 200–203—species-group treatment for *Idarnes*.

**Description**

*Females*. Body length 1.3–2.8 mm. Ovipositor length 0.4–1.6 mm. Body colour yellow to black, metallic tinge mostly absent. Wings hyaline. Head transverse (1.2–1.4× as wide as high). Face sculpture reticulate. Malar sulcus absent. Maxillary and labial palpi composed at most of two or three segments, the last one frequently reduced and setae-like. Clypeal margin bilobed. Frontal depression (scrobal cavity) shallow, rarely including median ocellus. Supraclypeal area delimited laterally by subantennal grooves. Antennae inserted closer to clypeal margin than to median ocellus (sometimes nearly equidistant from them). Toruli separated by one torulus diameter or less, but never closer than 0.5× torulus diameter. Antenna with 12–13 antennomeres (11–12 flagellomeres; one or two anelli) and a very small terminal protuberance. Clava not well delimited. Vertex slightly concave. POL 2.2–3.4× OOL. Mesosoma slightly curved dorsally. Pronotum 0.7–1.0× as long as mesoscutum. Notaulus complete and usually crenulated. Mesoscutellum 1.1–1.5× as long as wide near transscutal articulation. Metascutellum transverse, at most as long as frenum, sometimes inconspicuous. Postmarginal vein very short, at most 0.5× as long as stigmal vein. Ovipositor sheaths shorter than body length, and 0.8–3× as long as hind tibia.

*Males.* Similar to females, sometimes showing variation in tinge/colour and body proportions (see Supplemental Information 4). Flagellum shorter than in females. Wings medially infuscated, with dense pilosity, especially at the costal and apical margin, and near marginal vein.

**Remarks.** *Idarnes* is treated here as masculine as explained in Farache et al. (2013).

### Key to species

The key is based on female characters. *I. brasiliensis* (Mayr, 1906) was not included since only one male could be analysed.

1 Body completely black or dark brown (Figs. 2G, 3C, 3E), including pronotum (Figs. 8G, 9C, 9E) and propodeum (Figs. 10G, 11C, 11E). Scrobal depression and face engraved to reticulated (Figs. 4G, 5C, 5E)..............................................................................**2**
— Body not completely black. Pronotum yellow at least laterally (as in Figs. 2B, 2F, 3A), or propodeum yellow (as Figs. 10C, 10H, 11J). Scrobal depression mostly smooth, face sculpture engraved to reticulated (as in Figs. 4A, 4C, 4E)....................................**4**
2 Mesoscutum and scutellar-axillar complex strongly curved in lateral view (Fig. 3C). Supraclypeal area narrower than torulus (Fig. 5C). Frenal sulcus crenulated and conspicuous (Fig. 13C). Metascutellum nearly as long as, or longer than frenum (Fig. 13C). Ex *F. americana* subsp. *andicola*. ........................................................... ............................................................................................ ***I. gibberosus*** **sp. n.**
— Mesoscutum and scutellar-axillar complex not strongly curved in lateral view (Figs. 2G, 3E). Supraclypeal area as wide as, or wider than torulus (Figs. 4G, 5E). Frenal sulcus not crenulated (Figs. 12G, 13E). Metascutellum inconspicuous in dorsal view (Figs. 12G, 13E). Ex *F. hartwegii*. ......................................................................................**3**
3 Frons becoming yellowish near clypeus. Supraclypeal area as wide as torulus (Fig. 4G). Antenna with two anelli (Fig. 6G). Postmarginal vein nearly absent (Fig. 14G). Ex *F. hartwegii*. ...............................................................................***I. comptoni*** **sp. n.**
— Frons completely black. Supraclypeal area wider than torulus (Fig. 5E). Antenna with one anellus (Fig. 7E). Postmarginal vein nearly as long as 0.5× stigmal vein length (Fig. 15E). Ex *F. hartwegii.* ...............................................***I. hansoni*** **Bouček, 1993**
4 Head and mesosoma brown black in lateral view; pronotum yellow (Figs. 2B, 2F, 2H, 3A), at least laterally, or propodeum yellow dorsally (Figs. 10C, 10H).......................**5**
— Head and mesosoma predominantly yellow in lateral view (as in Figs. 2A, 2D, 2E)..........................................................................................................................**9**
5 Axillula reticulated, without longitudinal striae (Fig. 13A). Notauli not crenulated (Fig. 13A). Ex *F. americana.* .........................................................***I. flavicrus*** **sp. n.**
— Axillula longitudinally striated (Figs. 12B, 12C, 12F, 12H). Notauli at least slightly crenulated (Figs. 12B, 12C, 12F, 12H). ................................................................................**6**
6 Propodeum dorsally yellow (Figs. 10C, 10H). Frenal sulcus conspicuous (Figs. 12C, 12H). Body length 1.8–2.1 mm. .............................................................................................**7**
— Propodeum dorsally brown (Figs. 10B, 10F). Frenal sulcus inconspicuous (Figs. 12B, 12F). Body length 1.3–1.6 mm. ............................................................................................**8**
7 Pronotum laterally brown. Legs brown. Ovipositor 1.1–1.3× hind tibia length (Fig. 2C). Ex *F. americana* & *F. costaricana.* ........................................***I. americanae*** **sp. n.**
— Pronotum laterally yellow. Legs predominantly yellow. Ovipositor 1.5× hind tibia length (Fig. 2H). Ex *F. cremersi.* ..................................................***I. cremersiae*** **sp. n.**
8 Hind coxae yellow (Fig. 2B). Propodeal median line absent (Fig. 12B). Ex *F. americana* subsp. *guianensis* form *mathewsii.*....................................***I. amazonicus*** **sp. n.**
— Hind coxae brown (Fig. 2F). Propodeal median line present as a faint longitudinal reticulation (Fig. 12F). Ex *F. colubrinae.* .............................***I. brunneus*** **sp. n.**
9 Ovipositor nearly 2.5× as long as hind tibia or longer (Figs. 3B, 3D, 3G). .............**10**
— Ovipositor as long as or shorter than 2× hind tibia (as in Figs. 2I, 3I, 3J). ............**12**
10 Metasoma laterally yellow (Fig. 3B), dorsally with brown black transversal stripes. Frenal sulcus conspicuous (Fig. 13B). Ex *F. aurea* form *isophlebia.* ...................................... ................................................................................***I. flaviventris*** **sp. n.**
— Metasoma black (Figs. 3D, 3G). Frenal sulcus inconspicuous, sparsely crenulated (Figs. 13D, 13G). ..................................................................................................................**11**
11 Scutellar-axillar complex dorsally black (Fig. 11G). Supraclypeal area narrower than torulus (Fig. 5G). Anterior margin of propodeum angulose medially (Fig. 11G). First funicular segment with 12–17 multiporous late sensilla (Fig. 7G). Ex *F. obtusifolia.* ...............................................................................................***I. maximus*** **sp. n.**
— Mesosoma dorsally yellow (Fig. 11D). Supraclypeal area wider than torulus (Fig. 5D). Anterior margin of propodeum concave medially (Fig. 11D). First funicular segment with 6–8 multiporous plate sensilla (Fig. 7D). Ex *F. popenoei.* ......***I. gordhi*** **sp. n.**
12 First funicular segment with more than four multiporous plate sensilla (as in Figs. 6E, 6I, 7I, 7J). ....................................................................................................................**13**
— First funicular segment with four our less multiporous plate sensilla (Figs. 6A, 6D). Colombia, Ex * F. pertusa.* .................................................................................**18**
13 Ovipositor as long as, or shorter than hind tibia (Figs. 2E, 2I)..............................**14**
— Ovipositor longer than hind tibia (as in Figs. 3H, 3I). ............................................**15**
14 Metasoma laterally yellow (Fig. 2E). Subantennal groove as long as torulus (Fig. 4E). Supraclypeal area wider than torulus (Fig. 4E). Ex *F. citrifolia.* .................................... ........................................................................................***I. brevis*** **sp. n.**
— Metasoma entirely black (Fig. 2I). Subantennal groove longer than torulus (Fig. 4I). Supraclypeal area as wide as torulus (Fig. 4I). Ex *F. citrifolia.* ........................... ..............................................................***I. dimorphicus*** **sp. n.**
15 Ovipositor 1.8–2× as long as hind tibia (Figs. 3H, 3I). Supraclypeal area narrower than torulus (Figs. 5H, 5I). ........................................................................................**16**
— Ovipositor 1× to 1.5× as long as hind tibia (Figs. 3F, 3J). Supraclypeal area as wide as torulus or wider (Fig. 5J). ...............................................................................**17**
16 Metasoma ventrally yellow (Fig. 3I). Distance from torulus to median ocellus 1.4× distance from torulus to oral margin (Fig. 5I). Antenna with one anellus. Ex *F crocata.* ........................................................................................***I. pseudoflavus*** **sp. n.**
— Metasoma ventrally brown-black (Fig. 3H). Distance from torulus to median ocellus 0.9× distance from torulus to oral margin (Fig. 3H). Antenna with 2 anelli. *F. aurea* form * isophlebia.* .........................................................................***I. nigriventris*** **sp. n.**
17 Scutellar-axillar complex smoky yellow, propodeum more yellow (Fig. 11J). Metasoma brown black (Fig. 3J). POL 3× OOL Ex *F. americana* subsp. *americana.*.........................................................................................................***I. ramirezi*** **sp. n.**
— Scutellar-axillar complex yellow, nearly same colour as propodeum (Fig. 11F). Metasoma brown black, first tergite and ventral region yellow (Fig. 3F). POL 2.2× OOL Ex *F. aurea* & *F. citrifolia.*.......................................................................................... ..........................................................................................***I. incertus*** **(Ashmead, 1900)**
18 Head and mesosoma dorsally brown, slightly metallic green (Fig. 8D). Frenal sulcus inconspicuous (Fig. 12D). Ex *F. pertusa.* ..........................***I. badiovertex*** **sp. n.**
— Head and mesosoma yellow (Fig. 8A). Frenal sulcus conspicuous (Fig. 12A). Ex *F. pertusa.*......................................................................***I. amacayacuensis*** **sp. n.**

#### Species descriptions

**Table:** 

***Idarnesamacayacuensis*Farache & Rasplus, sp. n.**
urn:lsid:zoobank.org:act:48D01597-E7B0-41AC-8A7E-DCE21AA97EE6
(Figs. 2A, 4A, 6A, 8A, 10A, 12A, 14A)

**Type material.** Holotype: ♀, **COLOMBIA:** **Leticia:** PN Amacayacu, −3.30°, −70.14°, 130 m, 20.XI.1993, Lachaise D., ex *Ficus pertusa* (CBGP).

Paratypes: **COLOMBIA:** **Leticia:** PN Amacayacu, −3.30°, −70.14°, 130 m, 6♀, 5♂, 20.XI.1993, Lachaise D., ex *Ficus pertusa* (4♀ 4♂ CBGP, 2♀ 1♂ RPSP).

**Etymology**. The specific name refers to the type locality, the Amacayacu National Natural Park in Colombia.

**Diagnosis** (♀). Head, antennae, mesosoma, and legs yellow. Metasoma dark brown. Metascutellum inconspicuous in dorsal view. Propodeal median line present, traceable at least in the anterior half of propodeum. Ovipositor sheaths 1.6× as long as hind tibia.

**Female**.

*Size and colour.* Body length 1.5 mm. Ovipositor length 0.5 mm. Head, antennae, mesosoma, and legs yellow. Metasoma dark brown.

*Head.* Supraclypeal area narrower than torulus. Subantennal groove as long as torulus. Distance from torulus to median ocellus 1.3× distance from torulus to oral margin. POL 3.0× OOL. Scape 1.9× as long as pedicel. Antenna with two anelli (character sometimes inconspicuous). First funicular segment 0.7× as long as wide, with 1–3 multiporous plate sensilla.

*Mesosoma.* Mesoscutum reticulate. Mesoscutum and scutellar-axillar complex not strongly curved in lateral view. Notaulus crenulated. Mesoscutellum 1.2× as long as wide near transscutal articulation. Axillula with longitudinal striae. Frenal sulcus barely crenulated, conspicuous. Metascutellum inconspicuous in dorsal view. Anterior margin of propodeum angulose medially. Propodeal median line present, traceable at least in the anterior half of propodeum. Stigmal vein 0.9–1× as long as marginal vein. Stigmal vein without adstigmal setae. Postmarginal vein very short, as long as 1/3× stigmal vein length.

*Metasoma.* Ovipositor sheaths 1.6× as long as hind tibia.

**Male**. Similar to female. Body colour paler. Mesoscutellum infuscated. Distance from torulus to median ocellus 1.5× distance from torulus to oral margin. POL 2.3 × OOL. First funicular segment 0.5× as long as wide.

**Host plant.** *Ficus pertusa* Linnaeus filius.

**Table:** 

***Idarnes amazonicus*Farache & Rasplus, sp. n.**
urn:lsid:zoobank.org:act:6F44A1B2-73CC-4267-9F02-AF4E7FF600BC
(Figs. 2B, 4B, 6B, 8B, 10B, 12B, 14B)

**Type material.** Holotype: ♀, **BRAZIL:** **Amazonas:** São Gabriel da Cachoeira, Igarapé da Cachoeirinha, −0.13° −67.09°, 19.XI.2007, Santos O.A., ex *Ficus americana* subsp. *guianensis* form *mathewsii* n° JRAS02147_03 (MZSP).

Paratypes. **BRAZIL:** **Amazonas:** São Gabriel da Cachoeira, Igarapé da Cachoeirinha, −0.13° −67.09°, 2♀, 19.XI.2007, Santos O.A., ex *Ficus americana* subsp. *guianensis* form *mathewsii* n° JRAS02147_03 (1♀ CBGP, 1♀ RPSP).

**Etymology**. The specific name refers to the province where the type was collected.

**Diagnosis** (♀). Body predominantly brown black. Pronotum and propodeum slightly yellow. Legs yellow, femora slightly brown. Supraclypeal area as wide as torulus, or slightly wider. Subantennal groove slightly longer than torulus. Distance from torulus to median ocellus 1× distance from torulus to oral margin. Metascutellum inconspicuous to about 0.5× as long as frenum in dorsal view. Ovipositor sheaths 1.3× as long as hind tibia.

**Female**.

*Size and colour.* Body length 1.4–1.5 mm. Ovipositor length 0.4 mm. Predominantly brown black. Lower face yellow. Scape and pedicel yellow, pedicel slighly brown. Pronotum laterally yellow. Propodeum slightly yellow near its posterior margin. Legs yellow, femora slightly brown.

*Head.* Supraclypeal area as wide as torulus. Subantennal groove as long as torulus. Distance from torulus to median ocellus 1× distance from torulus to oral margin. POL 2.5× OOL. Scape 2.3× as long as pedicel. Antenna with two anelli (character sometimes inconspicuous). First funicular segment 0.5× as long as wide, with 6–9 multiporous plate sensilla.

*Mesosoma.* Mesoscutum reticulate. Mesoscutum and scutellar-axillar complex not strongly curved in lateral view. Notaulus with shallow crenulation. Mesoscutellum 1.4× as long as wide near transscutal articulation. Axillula longitudinally striate to reticulate. Frenal sulcus barely crenulated and inconspicuous. Metascutellum inconspicuous to approximately 0.5× as long as frenum in dorsal view. Anterior margin of propodeum concave medially. Propodeal median line inconspicuous. Stigmal vein 0.7× as long as marginal vein, with 3 adstigmal setae. Postmarginal vein very short, as long as 1/3× stigmal vein length.

*Metasoma.* Ovipositor sheaths 1.3× as long as hind tibia.

**Male**. Unknown.

**Host plant**. *Ficus americana* Aublet subsp. *guianensis* (Desvaux) Berg form *mathewsii* (Miquel) Berg.

**Table:** 

***Idarnes americanae*Farache & Rasplus, sp. n.**
urn:lsid:zoobank.org:act:0FF58956-AEB0-45C4-8AF9-3BB4C8EF2465
(Figs. 2C, 4C, 6C, 8C, 10C, 12C, 14C)

**Type material.** Holotype: ♀, **COSTA RICA:** **La Fortuna:** Arenal, 10.49916° −84.71019°, 18.IV.2010, Cruaud A. & Rasplus J.Y., ex *Ficus americana* n° JRAS02841_01 (CBGP).

Paratypes. **COSTA RICA:** **Heredia:** Santo Domingo, 9.98° −84.71°, 4♀, 3♂, 15.XI.2002, Hanson P., ex. *Ficus costaricana* n° JRAS01364 (3♀ 2♂ CBGP, 1♀ 1♂ RPSP); **La Fortuna:** Arenal, 10.49916° −84.71019°, 4♀, 3♂, 18.IV.2010, Cruaud A. & Rasplus J.Y., ex *Ficus americana* n° JRAS02841_01 (2♀ 2♂ CBGP, 1♀ 1♂ MZSP, 1♀ BMNH).

**Etymology**. The specific name refers to the *Ficus* section to which *Idarnes* is associated with.

**Diagnosis** (♀). Body colour and legs predominantly brown. Pronotum and propodeum mostly yellow. Supraclypeal area narrower than torulus. Subantennal groove shorter than torulus. Distance from torulus to median ocellus 1.2× distance from torulus to oral margin. Mesoscutum medially with longitudinal striae. Metascutellum nearly 0.5× as long as frenum or shorter in dorsal view. Ovipositor sheaths 1.1–1.3× as long as hind tibia.

**Female**.

*Size and colour.* Body length 1.9–2.1 mm. Ovipositor length 0.5–0.6 mm. Predominantly brown. Scape yellow. Pedicel yellow brown, flagellum brown. Pronotum laterally yellow. Lateral panel of metanotum brown black. Propodeum yellow. Legs brown, fore coxa and tarsi yellow. Fore tibia yellow brown.

*Head.* Supraclypeal area narrower than torulus. Subantennal groove shorter than torulus. Distance from torulus to median ocellus 1.2× distance from torulus to oral margin. POL 3.4× OOL. Scape 2.3× as long as pedicel. Antenna with two anelli (character sometimes inconspicuous). First funicular segment 0.7× as long as wide, with 7–9 multiporous plate sensilla.

*Mesosoma.* Mesoscutum reticulate to punctate reticulate, medially with longitudinal striae. Mesoscutum and scutellar-axillar complex not strongly curved in lateral view. Notaulus crenulated. Mesoscutellum 1.3× as long as wide near transscutal articulation. Axillula with longitudinal striae. Frenal sulcus crenulated and conspicuous. Metascutellum nearly 0.5× as long as frenum or shorter in dorsal view. Anterior margin of propodeum angulose medially. Propodeal median line present, traceable at least in the anterior half of propodeum. Stigmal vein as long as marginal vein, with 2-3 adstigmal setae. Postmarginal vein very short, as long as 1/3× stigmal vein length.

*Metasoma.* Ovipositor sheaths 1.1–1.3× as long as hind tibia.

**Male**. Body predominantly yellow. Lateral panel of metanotum yellow brown. Metasoma brown, yellow at the margin of tergites. Distance from torulus to median ocellus 1.5× distance from torulus to oral margin. POL 3.1× OOL. First funicular segment 0.5× as long as wide.

**Host plant**. *Ficus americana* subsp. *americana* Aublet and *Ficus costaricana* (Liebmann) Miquel.

**Table:** 

***Idarnes badiovertex*Farache & Rasplus, sp. n.**
urn:lsid:zoobank.org:act:28673472-103B-4576-A41E-4851E4194771
(Figs. 2D, 4D, 6D, 8D, 10D, 12D, 14D)

**Type material.** Holotype: ♀, **COLOMBIA:** **Leticia:** PN Amacayacu, −3.30° −70.14°, 130 m, 20.XI.1993, Lachaise D., ex *Ficus pertusa* (CBGP).

Paratypes: **COLOMBIA:** **Leticia:** PN Amacayacu, −3.30° −70.14°, 130 m, 16♀, 1♂, 20.XI.1993, Lachaise D., ex *Ficus pertusa* (13♀ 1♂ CBGP, 3 ♀ RPSP).

**Etymology**. The specific name refers to the brown colouration of the top of the head.

**Diagnosis** (♀). Head yellow, dorsally brown. Mesosoma dorsally brown black, axillula slightly metallic green. Propodeum yellow. First funicular segment with 1–2 multiporous plate sensilla. Ovipositor sheaths ca. 1.8× as long as hind tibia.

**Female**.

*Size and colour.* Body length 1.3 mm. Ovipositor length 0.5 mm. Head and mesosoma yellow. Antennae yellow. Head dorsally brown, slightly metallic green. Mesosoma dorsally brown black, axillula slightly metallic green. Propodeum dorsally yellow. Legs yellow. Metasoma brown.

*Head.* Supraclypeal area as wide as torulus. Subantennal groove shorter than torulus. Distance from torulus to median ocellus 1.4× distance from torulus to oral margin. POL 3× OOL. Scape 1.7–1.8× as long as pedicel. Antenna with two anelli (character sometimes inconspicuous). First funicular segment 0.5× as long as wide, with 0–2 multiporous plate sensilla.

*Mesosoma.* Mesoscutum reticulate. Mesoscutum and scutellar-axillar complex not strongly curved in lateral view. Notaulus with shallow crenulation. Mesoscutellum 1.4× as long as wide near transscutal articulation. Axillula with longitudinal striae. Frenal sulcus smooth and faint. Metascutellum inconspicuous in dorsal view. Anterior margin of propodeum angulose medially. Propodeal median line inconspicuous. Stigmal vein 0.9× as long as marginal vein, with 3-4 adstigmal setae. Postmarginal vein nearly absent, shorter than 1/5× stigmal vein length.

*Metasoma.* Ovipositor sheaths ca. 1.8× as long as hind tibia.

**Male**. Similar to female. Body colour paler.

**Host plant.***Ficus pertusa* Linnaeus filius.

**Table:** 

***Idarnes brasiliensis*(Mayr, 1906) (comb. nov.)**
(Figs in Supplemental Information 5)

1906 Mayr, G. *Entomologische Zeitung Wien* 25:185. Description (♀♂) (Comb. *Sycophila brasiliensis*).

**Type material.** Lectotype (here designated) **BRAZIL:** **Santa Catarina:** Blumenau, 1♂, [no date], Fritz Müller, ex *Ficus doliaria* (=*F. gomelleira*) (NMW).

**Diagnosis** (♂). Body colour predominantly yellow orange. Mesosoma 1.4× as long as wide. Axillula longitudinally striated. Frenal sulcus inconspicuous. Metascutellum inconspicuous in dorsal view. Propodeal median line present, conspicuous. Postmarginal vein very short, as long as 1/3× stigmal vein length.

**Female:** Described by Mayr (1906), but we could not find any female specimens at NMW.

**Host plant**. *Ficus gomelleira* Kunth & Bouché

**Remarks:** There is only one male specimen collected by Mayr at NMW, minuten-mounted and decapitated. Despite the absence of head, the following characters ascertain its position within the *Idarnes incertus* species-group: (1) winged male, (2) body colour, (3) postmarginal vein compared to stigmal vein, (4) shape of mesoscutellum and (4) striated axillula.

**Table:** 

***Idarnes brevis*Farache & Rasplus, sp. n.**
urn:lsid:zoobank.org:act:06317A88-E1C5-48AB-83FB-66E79424360C
(Figs. 2E, 4E, 6E, 8E, 10E, 12E, 14E)

**Type material.** Holotype: ♀, **COSTA RICA:** **San José:** Santiago de Puriscal, 9.84132°−84.31540°, 2.I.2007, Fernandez, ex *Ficus citrifolia* n° JRAS01954_02 (CBGP).

Paratypes. **COSTA RICA:** **Alajuela:** San Ramon, Piedades Sur 10.11° −84.53°, 23♀, 16♂, 5.I.2008, Vasquez J., ex *Ficus citrifolia* n° JRAS03857 (20♀ 15 ♂ CBGP, 3♀ 1♂ RPSP); **Heredia:** Santo Domingo, 9.988886° −84.083926°, 5♀, X.2005, Hanson P., ex *Ficus hemsleyana* (= *F. citrifolia*) n° JRAS01530_02 (1♀ CBGP, 2♀ RPSP 1♀ MZSP, 1♀ SAMC); **San José:** Santiago de Puriscal, 9.84132° −84.31540°, 1♀, 7♂, 2.I.2007, Fernandez, ex *Ficus citrifolia* n° JRAS01954_02 (1♀ 7♂ CBGP), Univ. San José, Est. Fabio B. Moreno, 10.00° −84.27°, 3♀, 5.III.2008, Rasplus J.Y. & Ramírez W., ex *Ficus hemsleyana* (= *F. citrifolia*) n° JRAS02284_03 (CBGP).

**Etymology**. The specific name refers to the short ovipositor.

**Diagnosis** (♀). Predominantly yellow orange. Metasoma dorsally brown black, mostly at the margin of tergites. Supraclypeal area wider than torulus. Subantennal groove as long as torulus. Distance from torulus to median ocellus 0.9× distance from torulus to oral margin. First funicular segment with 12–15 multiporous plate sensilla. Ovipositor sheaths 0.8–0.9× as long as hind tibia.

**Female**.

*Size and colour.* Body length 2.1–2.3 mm. Ovipositor length 0.4–0.5 mm. Predominantly yellow orange. Metasoma dorsally brown black, mostly at the margin of tergites.

*Head.* Supraclypeal area wider than torulus. Subantennal groove nearly as long as torulus. Distance from torulus to median ocellus 0.9× distance from torulus to oral margin. POL 3.1× OOL. Scape 2.2–2.5× as long as pedicel. Antenna with two anelli. First funicular segment 0.7–0.8× as long as wide, with 12–15 multiporous plate sensilla.

*Mesosoma.* Mesoscutum reticulate. Mesoscutum and scutellar-axillar complex not strongly curved in lateral view. Notaulus crenulated. Mesoscutellum 1.3× as long as wide near transscutal articulation. Axillula with longitudinal striae. Frenal sulcus conspicuous, barely crenulated or crenulated. Metascutellum nearly as long as, or longer than frenum in dorsal view. Anterior margin of propodeum angulose medially. Propodeal median line present, traceable at least in the anterior half of sclerite. Stigmal vein 0.9× as long as marginal vein, with 2 adstigmal setae. Postmarginal vein very short, as long as 1/3× stigmal vein length.

*Metasoma.* Ovipositor sheaths 0.8–0.9× as long as hind tibia.

**Male**. Similar to female. Predominant body colour paler, yellow white. Metasoma dorsally brown, or sometimes completely brown. Distance from torulus to median ocellus 1.3× distance from torulus to oral margin. POL 2.1× OOL. Mesoscutellum 1.6× as long as wide near transscutal articulation.

**Host plant**. *Ficus citrifolia* Miller.

**Table:** 

***Idarnes brunneus*Farache & Rasplus, sp. n.**
urn:lsid:zoobank.org:act:67D1DC74-C6AF-4E66-9897-1DC515D4F253
(Figs. 2F, 4F, 6F, 8F, 10F, 12F, 14F)

**Type material.** Holotype: ♀, **COSTA RICA:** **Limón:** near Bananito, 9.838917° −83.048111°, 15.IV.2010, Cruaud A. & Rasplus J.Y., ex *Ficus colubrinae* n° JRAS02832_05 & JRAS02833 (CBGP).

Paratypes. **COSTA RICA:** **Limón:** 3 km W Guacimo, 10.210894° −83.770628°, 1♀, 2.III.2008, Rasplus J.Y. & Ramírez W., ex *Ficus colubrinae* n° JRAS02282_03 (CBGP), near Bananito, 9.838917° −83.048111°, 17♀, 7♂, 15.IV.2010, Cruaud A. & Rasplus J.Y., ex *Ficus colubrinae* n° JRAS02832_05 & JRAS02833 (12♀ 3♂ CBGP, 2♀ 1♂ RPSP, 1♀ 1♂ MZSP, 1♀ 1♂ BMNH, 1♀ 1♂ SAMC).

**Etymology**. The specific name refers to the predominant body colour.

**Diagnosis** (♀). Body colour predominantly dark brown. Pronotum laterally yellow. Legs yellow, femora and coxae yellow brown. Supraclypeal area as wide as torulus, or slightly narrower. Subantennal groove as long as torulus. Distance from torulus to median ocellus 0.9× distance from torulus to oral margin. Metascutellum nearly 0.5× as long as frenum to inconspicuous in dorsal view. Ovipositor sheaths 1.5× as long as hind tibia.

**Female**.

*Size and colour.* Body length 1.3–1.6 mm. Ovipositor length 0.5 mm. Predominantly dark brown. Scape yellow. Pedicel and flagellum yellow brown. Lower face yellow. Pronotum laterally yellow. Legs yellow, femora and coxae yellow brown.

*Head.* Supraclypeal area as wide as torulus. Subantennal groove as long as torulus. Distance from torulus to median ocellus 0.9× distance from torulus to oral margin. POL 2.9× OOL. Scape 2.1× as long as pedicel. Antenna with two anelli (character sometimes inconspicuous). First funicular segment 0.7–0.8× as long as wide, with 5–9 multiporous plate sensilla.

*Mesosoma.* Mesoscutum reticulate to punctate reticulate. Mesoscutum and scutellar-axillar complex not strongly curved in lateral view. Notaulus crenulated. Mesoscutellum 1.2× as long as wide near transscutal articulation. Axillula longitudinally striate to reticulate. Frenal sulcus barely crenulated and inconspicuous. Metascutellum nearly 0.5× as long as frenum to inconspicuous in dorsal view. Anterior margin of propodeum concave medially. Propodeal median line present as a faint longitudinal reticulation. Stigmal vein 0.9× as long as marginal vein, with 2–3 adstigmal setae. Postmarginal vein very short, as long as 1/3× stigmal vein length.

*Metasoma.* Ovipositor sheaths 1.5× as long as hind tibia.

**Male**. Similar to female. Funicular segments, pronotum and legs yellow. Distance from torulus to median ocellus 1.2× distance from torulus to oral margin. POL 2.6× OOL.

**Host plant**. *Ficus colubrinae* Standley.

**Table:** 

***Idarnes comptoni*Farache & Rasplus, sp. n.**
urn:lsid:zoobank.org:act:3D938DAE-2869-40B2-888B-041FC96A7FDB
(Figs. 2G, 4G, 6G, 8G, 10G, 12G, 14G)

**Type material.** Holotype: ♀, **COSTA RICA:** **Puntarenas:** 8 km N Ciudad Neily, 8.712278°−82.937611°, 23.IV.2010, Cruaud A. & Rasplus J.Y., ex *Ficus hartwegii* n° JRAS02861 (CBGP).

Paratypes: **COSTA RICA:** **Puntarenas:** 8 km N Ciudad Neily, 8.712278° −82.937611°, 4♀, 1♂, 23.IV.2010, Cruaud A. & Rasplus J.Y., ex *Ficus hartwegii* n° JRAS02861 (2 ♀ 1 ♂ CBGP, 2 ♀ RPSP).

**Etymology**. The species is dedicated to our friend and colleague, Dr Stephen G. Compton, for his great contribution to the study of fig wasps and figs.

**Diagnosis** (♀). Body colour mostly brown. Frons more yellow near clypeus. Supraclypeal area as wide as torulus. Flagellum with 2 anelli. Propodeum dorsally yellow. Postmarginal vein nearly absent. Legs predominantly yellow. Axillula longitudinally striate to reticulate.

**Female**.

*Size and colour.* Body length 1.1–1.3 mm. Ovipositor length 0.5 mm. Body colour mostly brown. Scape and pedicel yellow. Flagellomeres yellow brown. Frons more yellow near clypeus. Head and mesosoma with faint metallic luster. Tibiae and tarsi yellow.

*Head.* Supraclypeal area as wide as torulus. Subantennal groove as long as torulus. Distance from torulus to median ocellus 1× distance from torulus to oral margin. POL 2.8× OOL. Scape 2× as long as pedicel. Antenna with two anelli. First funicular segment 0.6× as long as wide, with 3–4 multiporous plate sensilla.

*Mesosoma.* Mesoscutum reticulate. Mesoscutum and scutellar-axillar complex not strongly curved in lateral view. Notaulus mostly without crenulation. Mesoscutellum 1.1× as long as wide near transscutal articulation. Axillula longitudinally striate to reticulate. Frenal sulcus barely crenulated, inconspicuous. Metascutellum inconspicuous in dorsal view. Anterior margin of propodeum concave medially. Propodeal median line present as a faint longitudinal reticulation. Stigmal vein 0.6× as long as marginal vein, with three adstigmal setae. Postmarginal vein nearly absent, shorter than 1/5× stigmal vein length.

*Metasoma.* Ovipositor sheaths 1.7–1.8× as long as hind tibia.

**Male**. Similar to female.

**Host plant.** *Ficus hartwegii* (Miquel) Miquel.

**Table:** 

***Idarnes cremersiae*Farache & Rasplus, sp. n.**
urn:lsid:zoobank.org:act:5F8B227F-7568-4965-BBA4-41C8AEE08EB3
(Figs. 2H, 4H, 6H, 8H, 10H, 12H, 14H)

**Type material.** Holotype: ♀, **FRENCH GUIANA:** savanne roche, route de Kourou à Sinnamary, 5.115317°−52.783200°, 16.V.2011, Conchou L., ex *Ficus cremersii*, n° JRAS03711 (CBGP).

Paratypes: **FRENCH GUIANA:** savanne roche, route de Kourou à Sinnamary, 5.115317°−52.783200°, 2♀, 2♂, 16.V.2011, Conchou L., ex *Ficus cremersii*, n° JRAS03711 (1♀ 1♂ CBGP, 1♀ 1♂ RPSP).

**Etymology**. The specific name refers to the host plant.

**Diagnosis** (♀). Head yellow, brown in dorsal view. Mesosoma predominantly brown. Pronotum in lateral view and prepectus mostly yellow. Axillula longitudinally striate to reticulate. Ovipositor sheaths 1.5× as long as hind tibia.

**Female**.

*Size and colour.* Body length 1.8–2 mm. Ovipositor length 0.6 mm. Head yellow, brown in dorsal view. Scape yellow, pedicel and flagellomeres yellow brown. Mesosoma predominantly brown. Pronotum in lateral view and prepectus mostly yellow. Axillula slightly metallic green. Propodeum yellow. Legs predominantly yellow, slightly brown. Metasoma brown black.

*Head.* Supraclypeal area as wide as torulus. Subantennal groove as long as torulus. Distance from torulus to median ocellus 1× distance from torulus to oral margin. POL 2.7× OOL. Scape 2.2× as long as pedicel. Flagellum with 2 anelli. First funicular segment 0.9× as long as wide, with 8–11 multiporous plate sensilla.

*Mesosoma.* Mesoscutum reticulate. Mesoscutum and scutellar-axillar complex not strongly curved in lateral view. Notaulus with shallow crenulation. Mesoscutellum 1.2× as long as wide near transscutal articulation. Axillula longitudinally striate to reticulate. Frenal sulcus barely crenulated, conspicuous. Metascutellum nearly 0.5× as long as frenum to inconspicuous in dorsal view. Anterior margin of propodeum concave medially. Propodeal median line present, conspicuous. Stigmal vein 0.7× as long as marginal vein, with 1 adstigmal seta. Postmarginal vein nearly absent, shorter than 1/5× stigmal vein length.

*Metasoma.* Ovipositor sheaths 1.5× as long as hind tibia.

**Male**. Similar to female, body mostly pale yellow and wings medially infuscate. POL 1.7× OOL. Mesoscutellum 1.4× as long as wide near transscutal articulation.

**Host plant.** *Ficus cremersii* Berg.

**Table:** 

***Idarnes dimorphicus*Farache & Rasplus, sp. n.**
urn:lsid:zoobank.org:act:6C619C93-7DB1-437B-B4B9-0100C0F3886E
(Figs. 2I, 4I, 6I, 8I, 10I, 12I, 14I)

**Type material.** Holotype: ♀, **BRAZIL:** **São Paulo:** Gália, −22.30241° −49.62102°, 696 m, 9.VII.2009, Farache F.H.A., ex *Ficus citrifolia* n° FHAF00183_05 (MZSP).

Paratypes. **BRAZIL:** **Amazonas:** Manaus, −3.06°, −60.11°, 2♀, 23.VIII.2006, Santos, O.A., ex *Ficus citrifolia* n° FHAF00119_02 (RPSP), Manaus, −3.061583° −60.109444°, 30 m, 2♀, 3♂, 6.X.2011, Farache F.H.A. & Costa P.C., ex *Ficus citrifolia* n° FHAF00235_06 (RPSP), Manaus, Ponta Negra, Hotel Tropical, −3.06°, −60.11°, 6♀, 2♂, 23.VIII.2006, Santos O.A., ex *Ficus citrifolia* n° JRAS02136_02 (3♀ 1♂ RPSP, 3♀ 1♂ CBGP); **Rondônia:** Porto Velho, Estrada Belmont, −8.66937°, −63.91303°, 69 m, 19♀, 3♂, 28.VIII.2012, Farache F.H.A. & Costa P.C., ex *Ficus citrifolia* n° FHAF00329_02 (14♀ 1♂ CBGP, 5♀ 2♂ RPSP); **São Paulo:** Gália, −22.39544°, −49.78056°, 656 m, 5♀, 22.IX.2008, Cerezini M.T. & Farache F.H.A., ex *Ficus citrifolia* n° FHAF00065_07 (RPSP), Gália, −22.3748°, −49.6911°, 676 m, 5♀, 25.IX.2008, Farache F.H.A. & Pereira R.A.S., ex *Ficus citrifolia* n° FHAF00064_04 (CBGP), Gália, −22.30241°, −49.62102°, 696 m, 1♀, 9♂, 9.VII.2009, Farache F.H.A., ex *Ficus citrifolia* n° FHAF00183_05 (RPSP), Ribeirão Preto, −21.29459°, −47.90941°, 4♀, 4♂, 12.VII.2010, Farache F.H.A., ex *Ficus citrifolia* n° FHAF00198_01 (1♀ 1♂ MZSP, 3♀ 3♂ RPSP), Ribeirão Preto, −21.19216°, −47.78117°, 10♀, 1♂, 23.IV.2009, Cerezini M.T. & Teixeira L.M.R., ex *Ficus citrifolia* n° FHAF00099_03 (1♀ 1♂MZSP, 9♀ RPSP), Teodoro Sampaio, −22.3867°, −52.3106°, 445 m, 5♀, 3♂, 14.IX.2008, Farache F.H.A., ex *Ficus citrifolia* n° FHAF00171_04 (1♀ 1♂ BMNH, 1♀ 1♂ SAMC, 1♀ 1♂ MZSP, 2♀ RPSP).

**Etymology**. The name refers to the sexual colour dimorphism observed in this species.

**Diagnosis** (♀). Head and mesosoma predominantly yellow orange. Metasoma black. Predominant colour of males brown black. Supraclypeal area as wide as torulus. Subantennal groove longer than torulus. Distance from torulus to median ocellus 1–1.1× the distance from torulus to oral margin. Metascutellum nearly 0.3–1.0× as long as frenum in dorsal view. Anterior margin of propodeum angulose medially. Ovipositor sheaths 0.9–1× as long as hind tibia.

**Female**.

*Size and colour.* Body length 1.7–2.1 mm. Ovipositor length 0.4–0.5 mm. Head and mesosoma yellow orange. Vertex dark orange, black in ocellar margin. Antennae and legs paler. Metasoma black. Ovipositor sheaths 0.9–1× as long as hind tibia.

*Head.* Supraclypeal area as wide as torulus. Subantennal groove as long as, or slightly longer than torulus. Distance from torulus to median ocellus 1–1.1× distance from torulus to oral margin. POL 2.5–3× OOL. Scape 2–2.5× as long as pedicel. Antenna with two anelli. First funicular segment 0.7–0.9× as long as wide, with 9–14 multiporous plate sensilla.

*Mesosoma.* Mesoscutum reticulate. Mesoscutum and scutellar-axillar complex not strongly curved in lateral view. Notaulus crenulated. Mesoscutellum 1.2–1.3× as long as wide near transscutal articulation. Axillula with longitudinal striae. Frenal sulcus barely crenulated and conspicuous. Metascutellum nearly 0.3–1.0× as long as frenum in dorsal view. Anterior margin of propodeum angulose medially. Propodeal median line present, traceable at least in the anterior half of propodeum. Stigmal vein 0.9× as long as marginal vein, with 2–3 adstigmal setae. Postmarginal vein very short, as long as 1/3× stigmal vein length.

*Metasoma.* Ovipositor sheaths 0.9–1× as long as hind tibia.

**Male.** Similar to female. Body predominantly brown black. Legs distally yellow. Antennae yellow brown.

**Host plant**. *Ficus citrifolia* Miller.

**Table:** 

***Idarnes flavicrus*Farache & Rasplus, sp. n.**
urn:lsid:zoobank.org:act:4DFA1180-913B-48E1-B228-1ACE7F4603F0
(Figs. 3A, 5A, 7A, 9A, 11A, 13A, 15A)

**Type material.** Holotype: ♀, **COSTA RICA:** **La Fortuna:** Arenal, 10.49916° −84.71019°, 18.IV.2010, Cruaud A. & Rasplus J.Y., ex *Ficus americana* n° JRAS02841_01 (CBGP).

Paratypes: **COSTA RICA:** **La Fortuna:** Arenal, 10.49916° −84.71019°, 1♀, 2♂, 18.IV.2010, Cruaud A. & Rasplus J.Y., ex *Ficus americana* n° JRAS02841_01 (1♀ 2♂ CBGP).

**Etymology**. The specific name refers to the yellow legs contrasting with the dark brown mesosoma and metasoma.

**Diagnosis** (♀). Head yellow orange. Mesosoma and metasoma predominantly brown black. Supraclypeal area narrower than torulus. Subantennal groove shorter than torulus. Distance from torulus to median ocellus 1–1.2× distance from torulus to oral margin. Notaulus nearly without crenulation. Axillula reticulate, without longitudinal striae. Frenal sulcus smooth and inconspicuous. Metascutellum inconspicuous in dorsal view. Wing with with 4 adstigmal setae. Ovipositor sheaths 2–2.1× as long as hind tibia.

**Female**.

*Size and colour.* Body length 1.8 mm. Ovipositor length 0.7–0.8 mm. Head yellow orange. Scape and pedicel yellow. Flagellum yellow brown. Mesosoma bown black. Pronotum laterally yellow. Legs yellow. Metasoma brown black

*Head.* Supraclypeal area narrower than torulus. Subantennal groove shorter than torulus. Distance from torulus to median ocellus 1–1.2× distance from torulus to oral margin. POL 2.9× OOL. Scape 2.3× as long as pedicel. Antenna with two anelli. First funicular segment 0.8–0.9× as long as wide, with approximately 6 multiporous plate sensilla.

*Mesosoma.* Mesoscutum slightly reticulate. Mesoscutum and scutellar-axillar complex not strongly curved in lateral view. Notaulus nearly without crenulation. Mesoscutellum 1.1× as long as wide near transscutal articulation. Axillula reticulate, without longitudinal striae. Frenal sulcus smooth and inconspicuous. Metascutellum inconspicuous in dorsal view. Anterior margin of propodeum concave medially. Propodeal median line present as a faint longitudinal reticulation. Stigmal vein 0.6× as long as marginal vein, with 4 adstigmal setae. Postmarginal vein short, as long as 1/3× stigmal vein length.

*Metasoma.* Ovipositor sheaths 2–2.1× as long as hind tibia.

**Male**. Similar to female. Body colour predominantly yellow. Vertex yellow brown. Mesosoma dorsally yellow brown, particularly brown at mesoscutum and scutellar-axillar complex. Metasoma brown black.

**Host plant**. *Ficus americana* subsp. *americana* Aublet.

**Table:** 

***Idarnes flaviventris*Farache & Rasplus, sp. n.**
urn:lsid:zoobank.org:act:8061097A-9783-4A71-8C5B-017C24BD48B5
(Figs. 3B, 5B, 7B, 9B, 11B, 13B, 15B)

**Type material.** Holotype: ♀, **COSTA RICA:** **Heredia:** Santo Domingo, 9.94952°−84.08068°, 12.IV.2010, Cruaud A. & Rasplus J.Y., ex *Ficus aurea* form *isophlebia* n° JRAS02809 (CBGP).

Paratypes. **COSTA RICA:** **Heredia:** Santo Domingo, 9.94952°−84.08068°, 8♀ 4 ♂, 12.IV.2010, Cruaud A. & Rasplus J.Y., ex *Ficus aurea* form *isophlebia* n° JRAS02809 (5♀ 4♂ CBGP, 1♀ BMNH, 2♀ MZSP, 1♀ SAMC); **Limón:** 12 Km SW Bribri, 9.559778°, −82.9135°, 6♀ 3♂ 21.IV.2010, Cruaud A. & Rasplus J.Y., ex. *Ficus aurea* form *isophlebia*, n° JRAS02829 (3♀ 3♂ CBGP, 3♀ RPSP), Puerto Viejo de Talamanca, 9.637585°, −82.708600, 10♀ 4 ♂, 14.IV.2010, Cruaud A. & Rasplus J.Y., ex *Ficus aurea* form *isophlebia* n° JRAS02824_02 (7♀ 2♂ CBGP, 3♀ 2♂ RPSP).

**Etymology**. The specific name refers to the coloration of the metasoma, especially in ventral view.

**Diagnosis** (♀). Body predominantly yellow orange. Margin of metasomal tergites brown. Subantennal groove shorter than torulus. Distance from torulus to median ocellus 1.3× distance from torulus to oral margin. Metascutellum inconspicuous to approximately 0.5× as long as frenum in dorsal view. Anterior margin of propodeum angulose medially. Propodeal median line present, traceable at least in the anterior half of propodeum. Ovipositor sheaths 2.7–2.9× as long as hind tibia.

**Female**.

*Size and colour.* Body length 2.2–2.3 mm. Ovipositor length 1.3–1.4 mm. Head, mesosoma, and antennae yellow orange. Legs more yellow. Metasoma yellow, margin of tergites brown.

*Head.* Supraclypeal area narrower than torulus. Subantennal groove shorter than torulus. Distance from torulus to median ocellus 1.3× distance from torulus to oral margin. POL 2.5× OOL. Scape 2.2–2.3× as long as pedicel. Antenna with two anelli (character sometimes inconspicuous). First funicular segment 0.7–0.8× as long as wide, with 7–8 multiporous plate sensilla.

*Mesosoma.* Mesoscutum reticulate to punctate reticulate. Mesoscutum and scutellar-axillar complex not strongly curved in lateral view. Notaulus crenulated. Mesoscutellum 1.1× as long as wide near transscutal articulation. Axillula longitudinally striate to reticulate. Frenal sulcus crenulated and conspicuous. Metascutellum inconspicuous to approximately 0.5× as long as frenum in dorsal view. Anterior margin of propodeum angulose medially. Propodeal median line present, traceable at least in the anterior half of propodeum. Stigmal vein 0.7× as long as marginal vein, with 1–2 adstigmal setae. Postmarginal vein very short, as long as 1/3× stigmal vein length.

*Metasoma.* Ovipositor sheaths 2.7–2.9× as long as hind tibia.

**Male.** Similar to female. Distance from torulus to median ocellus 1.7× distance from torulus to oral margin. POL 2.0× OOL. Antennal flagellum shorter than scape plus pedicel (longer in female).

**Host plant**. *Ficus aurea* Nuttal, form *isophlebia* (Standley) Berg. *Ficus isophlebia* was synonymized with *F. aurea*, however the differences observed between entities within the *F. aurea* species complex led C. C. Berg to recognize four informal entities within the species (Berg, 2007). Taking into account the morphological differences observed in the host-plants (*Ficus aurea* form *isophlebia* and *Ficus aurea* form *aurea*), the fact that these forms are sympatrically pollinated by different pollinator species and that non-pollinating communities associated with these forms are composed of different species (J-Y Rasplus, 2016, unpublished data), including different species of the *Idarnes incertus* species-group, we suspect that these forms of *Ficus aurea* may be in fact different but closely related *Ficus* species.

**Table:** 

***Idarnes gibberosus*Farache & Rasplus, sp. n.**
urn:lsid:zoobank.org:act:20EC9435-6547-4FFA-AC77-9205B471F40C
(Figs. 3C, 5C, 7C, 9C, 11C, 13C, 15C)

**Type material.** Holotype: ♀, **COLOMBIA: Cundinamarca:** Bogota, Ciudad Universitaria, 4.638568° −74.089985°, 2,620 m, 3.III.2006, Jansen-G. S., ex *Ficus americana* subsp. *andicola* n° JRAS01682_02 (CBGP).

Paratype. ♀, **COLOMBIA: Cundinamarca:** Bogota, Ciudad Universitaria, 4.638568°−74.089985°, 2,620 m, 3.III.2006, Jansen-G. S., ex *Ficus americana* subsp. *andicola* n° JRAS01682_02 (CBGP).

**Etymology**. The specific name refers to the mesoscutum, which is particularly curved in lateral view.

**Diagnosis** (♀). Body predominantly black. Supraclypeal area narrower than torulus. Subantennal groove longer than torulus. Distance from torulus to median ocellus 0.8× distance from torulus to oral margin. Mesoscutum and scutellar-axillar complex strongly curved in lateral view. Metascutellum nearly as long as, or longer than frenum in dorsal view. Ovipositor sheaths 2× as long as hind tibia.

**Female**.

*Size and colour.* Body length 1.8 mm. Ovipositor length 0.9 mm. Predominantly black. Scape brown, pedicel and flagellum yellow. Legs brown black. Tibia, tarsi, proximal portion of femur, trochanter and trochantellus yellow.

*Head.* Supraclypeal area narrower than torulus. Subantennal groove longer than torulus. Distance from torulus to median ocellus 0.8× distance from torulus to oral margin. POL 2.5× OOL. Scape 2.3× as long as pedicel. Antenna with two anelli. First funicular segment 0.7–0.8× as long as wide, with 8–9 multiporous plate sensilla.

*Mesosoma.* Mesoscutum reticulate to punctate reticulate. Mesoscutum and scutellar-axillar complex strongly curved in lateral view. Notaulus sparsely crenulated. Mesoscutellum 1.2× as long as wide near transscutal articulation. Axillula longitudinally striate to reticulate. Frenal sulcus crenulated and conspicuous. Metascutellum nearly as long as, or longer than frenum in dorsal view. Anterior margin of propodeum angulose medially. Propodeal median line present, conspicuous. Stigmal vein as long as 0.6× marginal vein, with 2 adstigmal setae. Postmarginal vein nearly as long as 0.5× stigmal vein length.

*Metasoma.* Ovipositor sheaths 2× as long as hind tibia.

**Male**. Not known.

**Host plant**. *Ficus americana* subsp*. andicola*(Standley) Berg.

**Table:** 

***Idarnes gordhi*Farache & Rasplus, sp. n.**
urn:lsid:zoobank.org:act:57B16D6D-A205-4F3A-B53E-E58399809FEC
(Figs. 3D, 5D, 7D, 9D, 11D, 13D, 15D)

**Type material.** Holotype: ♀, **COSTA RICA:** **Limón:** 8 Km W Guapiles 10.20650°−83.86173°, 13.IV.2010, *ex. Ficus popenoei*, n° JRAS02812_2, Cruaud A. & Rasplus, J.Y. leg. (CBGP).

Paratypes: **COSTA RICA:** **Limón:** 8 Km W Gualipes, 10.20650° −83.86173°, 5♀, 4♂, 13.IV.2010, Cruaud A. & Rasplus J.Y., ex *Ficus popenoei* n° JRAS02812_02 (3♀ 3♂ CBGP, 1♀ 1♂ RPSP, 1♀ MZSP).

**Etymology**. The species is dedicated to the renowned entomologist, Gordon Gordh.

**Diagnosis** (♀). Head and mesosoma yellow orange. Metasoma brown black. Supraclypeal area slightly wider than torulus. Subantennal groove shorter than torulus. Distance from torulus to median ocellus 1.0–1.1× distance from torulus to oral margin. Metascutellum inconspicuous in dorsal view. Ovipositor sheaths 2.4–2.5× as long as hind tibia.

**Female**.

*Size and colour.* Body length 2 mm. Ovipositor length 1.1–1.2 mm. Head and mesosoma yellow orange. Antennae and legs yellow orange. Lateral panel of metanotum brown black. Propodeum more yellow. Metasoma brown black.

*Head.* Supraclypeal area slightly wider than torulus. Subantennal groove shorter than torulus. Distance from torulus to median ocellus 1.1× distance from torulus to oral margin. POL 2.5× OOL. Scape 2.2× as long as pedicel. Antenna with two anelli (character sometimes inconspicuous). First funicular segment 0.8× as long as wide, with 6–8 multiporous plate sensilla.

*Mesosoma.* Mesoscutum reticulate. Mesoscutum and scutellar-axillar complex not strongly curved in lateral view. Notaulus crenulated, crenulation very shallow. Mesoscutellum 1.4× as long as wide near transscutal articulation. Axillula reticulate. Frenal sulcus barely crenulated and inconspicuous. Metascutellum inconspicuous in dorsal view. Anterior margin of propodeum concave medially. Propodeal median line present, traceable at least in the anterior half of propodeum. Stigmal vein as long as marginal vein, with 2–4 adstigmal setae. Postmarginal vein nearly absent, shorter than 1/5× stigmal vein length.

*Metasoma.* Ovipositor sheaths 2.4–2.5× as long as hind tibia.

**Male**. Similar to female. Body colour paler. Distance from torulus to median ocellus 1.7× distance from torulus to oral margin. POL 1.9× OOL. First funicular segment 0.5× as long as wide.

**Host plant**. *Ficus popenoei* Standley.

**Table:** 

***Idarnes hansoni*Bouček, 1993**
(Figs. 3E, 5E, 7E, 9E, 11E, 13E, 15E)

1993 Bouček, Z., *Journal of Natural History* 27: 202–203, Fig. 38. Description (♀♂).

**Type material.** Holotype: ♀, **COSTA RICA:** **San José:** Zarcero, Llano Bonito, XII.1987, Hanson P., ex *Ficus* (BMNH, examined).

Paratypes. **COSTA RICA: Guanacaste**: N.P. Santa Rosa, 1♀, I.1987, Gauld, I (BMNH); **San José:** Zarcero, Llano Bonito, 4♀, XII.1987, Hanson P., ex *Ficus* (BMNH), Zurqui de Moravia, 1600 m, 1♀, 2♂, 7-9.IX.1991, Hanson P., ex *Ficus brenesii*(= *F. hartwegii*) (EBCR, USNM, BMNH)

**Diagnosis** (♀). Body predominantly brown black. Supraclypeal area wider than torulus. Subantennal groove as long as torulus. Distance from torulus to median ocellus 0.9× distance from torulus to oral margin. Metascutellum inconspicuous in dorsal view. Anterior margin of propodeum concave medially. Ovipositor sheaths 1.4–1.5× as long as hind tibia.

Description: See Supplemental Information 5

**Host plant**. *Ficus hartwegii* (Miquel) Miquel. *Ficus brenesii* Standl. is considered a junior synonym of *F. hartwegii* (Miq.).

**Remarks.** One paratype analysed (Guanacaste, N. P. Santa Rosa, January 1987, I. Gauld leg. (BMNH)) actually belongs to an undescribed species. Since only one specimen is known and because we have no host information, we decided not to describe it waiting for more information and specimens. This species can be distinguished from *I. hansoni* by the following characters: (1) head, pronotum, and propodeum yellow brown, (2) propodeal median line present and conspicuous, (3) anterior margin of propodeum slightly angulose medially.

**Table:** 

***Idarnes incertus*(Ashmead, 1900)**
(Figs. 3F, 5F, 7F, 9F, 11F, 13F, 15F)

1900 Ashmead, W.H., *Transactions of the Entomological Society of London* 33:253 Description (♀ ♂) (Comb.: *Sycophila incerta*).

1993 Bouček, Z., *Journal of Natural History* 27: 202, Fig. 37. Lectotype designation. (Comb.: *Idarnes incerta*).

**Type material.** Lectotype. ♀, **USA:** Florida: Coconut Grove (USNM).

Paralectotypes: **ST. VINCENT:** 2♀, Smith H.H. (USNM). **USA:** **Florida:** Florida city, 1♂, V.1989, Nadel H., ex *Ficus citrifolia* (BMNH)

**Diagnosis** (♀). Body predominantly yellow orange. Metasoma dorsally brown black, first tergite yellow. Supraclypeal area as wide as torulus. Subantennal groove as long as torulus. Distance from torulus to median ocellus 1× distance from torulus to oral margin. Frenal sulcus smooth. Metascutellum nearly 0.5× as long as frenum to inconspicuous in dorsal view. Postmarginal vein nearly absent, shorter than 1/5× stigmal vein length. Ovipositor sheaths 1.4× as long as hind tibia.

Description: See Supplemental Information 5

**Host plant**. *Ficus aurea* form *aurea* Nuttal and *Ficus citrifolia* Miller

**Remarks.** Several specimens collected in Guadeloupe (38♀, 7♂, JRAS01219 & JRAS01220, CBGP, RPSP) are probably closely related to *Idarnes incertus*, yet, subtle morphological differences can be observed between these specimens and the type specimens from Florida. Consequently, *Idarnes incertus* may constitute a complex of species associated with *Ficus aurea* and *F. citrifolia* in Florida and in the Caribbean islands. Therefore this species may deserve thorough phylogeographical analyses using large sampling before a better species delimitation.

**Table:** 

***Idarnes maximus*Farache & Rasplus, sp. n.**
urn:lsid:zoobank.org:act:BA9EEC28-FD78-45B6-953A-11274C64995E
(Figs. 3G, 5G, 7G, 9G, 11G, 13G, 15G)

**Type material.** Holotype: ♀, **BRAZIL:** **São Paulo:** Gália, −22.2949°, −49.64812°, 31.III.2008, Farache F.H.A., ex *Ficus obtusifolia* n° FHAF00015_02 (MZSP).

Paratypes. **BRAZIL:** **São Paulo:** Araraquara, Road to Fazenda Salto Grande, −21.804685°−48.203512°, 634 m, 7♀, 7♂, 30.VII.2012, Farache F.H.A., ex *Ficus obtusifolia* n°FHAF00323_01 (3♀ 3♂ RPSP, 1♀1♂ CBGP, 1♀ 1♂ MZSP, 1♀ 1♂ BMNH, 1♀ 1♂ SAMC), Gália, −22.2949°−49.64812°, 5♀, 1♂, 31.03.2008, Farache F.H.A., ex *Ficus obtusifolia* n° FHAF00015_02 (3♀ 1♂ CBGP, 2♀ RPSP), Gália, −22.37042° −49.65974°, 1♀, 31.III.2008, Farache F.H.A., ex *Ficus obtusifolia* n° FHAF00011_12 (RPSP), Gália, −22.37852° −49.71912°, 3♀, 9.VII.2009, Teixeira L.M.R. & Medeiros M.D.F., ex *Ficus obtusifolia* n° FHAF00155_17 (RPSP), Gália, Road SP331, −22.37042°, −49.65974°, 680 m, 19♀, 20♂, 6.IX.2009, Pereira R.A.S., ex *Ficus obtusifolia* n° FHAF00201_05 (RPSP), Garça, −22.2916° −49.74199°, 666 m, 24♀, 5♂, 20.XI.2008, Teixeira L.M.R., ex *Ficus obtusifolia* n° FHAF00070_04 (RPSP), Ribeirão Preto, Bosque Municipal Fábio Barreto, −21.1734°−47.8018°, 550 m, 1♀, 2.VII.2006, Farache F.H.A. & do Ó V.T., ex *Ficus obtusifolia* n° FHAF00134_03 (RPSP).

**Etymology**. The specific name refers to the large body size of this species.

**Diagnosis** (♀). Head and mesosoma predominantly yellow orange in lateral view. Mesonotum and lateral panel of metascutum predominantly black in dorsal view. Metasoma brown black. Supraclypeal area narrower than torulus. Subantennal groove shorter than torulus. Distance from torulus to median ocellus 1.2× distance from torulus to oral margin. First funicular segment with 12–17 multiporous plate sensilla. Ovipositor sheaths 2.7–2.8× as long as hind tibia.

**Female**.

*Size and colour.* Body length 2.4–3.0 mm. Ovipositor length 1.4–1.6 mm. Head and mesosoma predominantly yellow orange in lateral view. Vertex brown, black near ocelli. Antenna yellow. Pronotum slightly brown. Mesonotum and lateral panel of metascutum predominantly black in dorsal view. Legs yellow orange. Mesepimeron black. Mesepisternum ventrally brown black. Metasoma brown black.

*Head.* Supraclypeal area narrower than torulus. Subantennal groove shorter than torulus. Distance from torulus to median ocellus 1.2× distance from torulus to oral margin. POL 2.2× OOL. Scape 2.3× as long as pedicel. Antenna with two anelli. First funicular segment 0.9×as long as wide, with 12–17 multiporous plate sensilla.

*Mesosoma.* Mesoscutum reticulate. Mesoscutum and scutellar-axillar complex not strongly curved in lateral view. Notaulus crenulated. Mesoscutellum 1.1–1.2× as long as wide near transscutal articulation. Axillula with longitudinal striae. Frenal sulcus barely crenulated and faint. Metascutellum nearly 0.5–1.0× as long as frenum in dorsal view. Anterior margin of propodeum angulose medially. Propodeal median line present, traceable at least in the anterior half of propodeum. Stigmal vein 0.8× as long as marginal vein, with 2 adstigmal setae. Postmarginal vein as long as 0.33–0.5× stigmal vein length.

*Metasoma.* Ovipositor sheaths 2.7–2.8× as long as hind tibia.

**Male**. Similar to female. Head yellow brown to brown, especially at frons and near vertex in some specimens. Distance from torulus to median ocellus 1.3–1.4× distance from torulus to oral margin. POL 1.8× OOL.

**Host plant**. *Ficus obtusifolia* Kunth

**Table:** 

***Idarnes nigriventris*Farache & Rasplus, sp. n.**
urn:lsid:zoobank.org:act:0BE190F5-9E67-45CE-9533-30F65905294E
(Figs. 3H, 5H, 7H, 9H, 11H, 13H, 15H)

**Type material.** Holotype: ♀, **COSTA RICA:** **Heredia:** Santo Domingo, 9.94952°−84.08068°, 12.IV.2010, Cruaud A. & Rasplus J.Y., ex *Ficus aurea* form *isophlebia* n° JRAS02809 (CBGP).

Paratypes. **COSTA RICA:** **Heredia:** Santo Domingo, 9.94952° −84.08068°, 3♀ 1 ♂, 12.IV.2010, Cruaud A. & Rasplus J.Y., ex *Ficus aurea* form *isophlebia* n° JRAS02809 (CBGP); **Limón:** 12 Km SW Bribri, 9.559778° −82.9135°, 6♀ 2♂ 21.IV.2010, Cruaud A. & Rasplus J.Y., ex *Ficus aurea* form *isophlebia*, n° JRAS02829 (2♀ 2♂ CBGP, 1♀ MZSP, 1♀ RPSP, 1♀ BMNH, 1♀ SAMC), Puerto Viejo de Talamanca, 9.637565 −82.708577, 3♀ 2♂, 14.IV.2010, Cruaud A. & Rasplus J.Y., ex *Ficus aurea* form *isophlebia* n° JRAS02824_02 (2♀ 1♂ CBGP, 1♀ 1♂ RPSP).

**Etymology**. The specific name refers to the colouration of the metasoma.

**Diagnosis** (♀). Head and mesosoma predominantly yellow orange. Metasoma brown black, first tergite yellow. Supraclypeal area narrower than torulus. Subantennal groove shorter than torulus. Distance from torulus to median ocellus 0.9× distance from torulus to oral margin. Metascutellum nearly 0.5× as long as frenum or shorter in dorsal view. Ovipositor sheaths 1.9–2.2× as long as hind tibia.

**Female**.

*Size and colour.* Body length 1.7–2.1 mm. Ovipositor length 0.8–0.9 mm. Head and mesosoma yellow orange. Antennae and legs paler. Metasoma brown black, first tergite yellow.

*Head.* Supraclypeal area narrower than torulus. Subantennal groove shorter than torulus. Distance from torulus to median ocellus 0.9× distance from torulus to oral margin. POL 2.8× OOL. Scape 2.3× as long as pedicel. Antenna with two anelli. First funicular segment 0.7×as long as wide, with 5–7 multiporous plate sensilla.

*Mesosoma.* Mesoscutum reticulate to punctate reticulate. Mesoscutum and scutellar-axillar complex not strongly curved in lateral view. Notaulus with shallow crenulation. Mesoscutellum 1.1× as long as wide near transscutal articulation. Axillula with longitudinal striae. Frenal sulcus crenulated and conspicuous. Metascutellum nearly 0.5× as long as frenum or shorter in dorsal view. Anterior margin of propodeum angulose medially. Propodeal median line present, traceable at least in the anterior half of propodeum. Stigmal vein 0.9× as long as marginal vein, with two adstigmal setae. Postmarginal vein very short, as long as 1/3× stigmal vein length.

*Metasoma.* Ovipositor sheaths 1.9–2.2× as long as hind tibia.

**Male**. Similar to female. Distance from torulus to median ocellus 1.2× distance from torulus to oral margin. POL 2.4× OOL.

**Host plant**. *Ficus aurea* Nuttal form* isophlebia* (Standley) Berg.

**Table:** 

***Idarnes pseudoflavus*Farache & Rasplus, sp. n.**
urn:lsid:zoobank.org:act:5E9EB253-0BCD-4EA4-A788-0FEED5DE482D
(Figs. 3I, 5I, 7I, 9I, 11I, 13I, 15I)

**Type material.** Holotype: ♀, **COSTA RICA:** **San José:** Pérez Zeledón, 9.337597° −83.641458°, 26.II.2008, Rasplus J.Y., ex *Ficus goldmanii* (= *F. crocata*) n° JRAS02182_02 (CBGP).

Paratypes. **COSTA RICA:** **Puntarenas:** Herradura, 9.65788° −84.63541°, 3♀, 1♂, 19.IV.2010, Cruaud A. & Rasplus J.Y., ex *Ficus goldmanii* (= *F. crocata*) n° JRAS02843_01 (CBGP); **San José:** Pérez Zeledón, 9.337597° −83.641458°, 68♀, 17♂, 26.II.2008, Rasplus J.Y., ex *Ficus goldmanii* (= *F. crocata*) n° JRAS02182_02 (63♀ 12♂ CBGP, 1♀ 1♂ MZSP, 2♀ 2♂ RPSP, 1♀ 1♂ BMNH, 1♀ 1♂ SAMC).

**Etymology**. The specific name refers to the predominant body colour.

**Diagnosis**. Head and mesosoma predominantly yellow orange. Metasoma yellow brown to black. Supraclypeal area narrower than torulus. Subantennal groove shorter than torulus. Distance from torulus to median ocellus 1.4× distance from torulus to oral margin. Metascutellum inconspicuous in dorsal view. Ovipositor sheaths 1.8× as long as hind tibia.

**Female**.

*Size and colour.* Body length 2–2.3 mm. Ovipositor length 0.8–0.9 mm. Head, mesosoma, antennae and legs predominantly yellow orange. Pronotum laterally yellow. Lateral panel of metanotum brown. Propodeum yellow. Mesepisternum, mesepimeron and mesocoxa slightly brown. Metasoma dorsally brown black, laterally yellow, and ventrally brown-yellow.

*Head.* Supraclypeal area narrower than torulus. Subantennal groove shorter than torulus. Distance from torulus to median ocellus 1.4× distance from torulus to oral margin. POL 2.9× OOL. Scape 2.3–2.6× as long as pedicel. Antenna with one anellus. First funicular segment 0.6–0.8× as long as wide, with 7–13 multiporous plate sensilla.

*Mesosoma.* Mesoscutum reticulate. Mesoscutum and scutellar-axillar complex not strongly curved in lateral view. Notaulus crenulated. Mesoscutellum 1.3× as long as wide near transscutal articulation. Axillula with longitudinal striae. Frenal sulcus crenulated. Metascutellum inconspicuous in dorsal view. Anterior margin of propodeum angulose medially. Propodeal median line present, traceable at least in the anterior half of propodeum. Stigmal vein 0.9× as long as marginal vein, with 2 adstigmal setae. Postmarginal vein very short, as long as 1/3× stigmal vein length.

*Metasoma.* Ovipositor sheaths 1.8× as long as hind tibia.

**Male**. Similar to female. Predominant body colour is paler, yellow white. Metasoma brown black except the first and second tergites, which are yellow. Distance from torulus to median ocellus 2.1× distance from torulus to oral margin. POL 2.2× OOL

**Host plant**. *Ficus crocata* (Miquel) Miquel.

**Table:** 

***Idarnes ramirezi*Farache & Rasplus, sp. n.**
urn:lsid:zoobank.org:act:470B1ABF-F6C6-4C8B-BBE5-69ABD5548EA8
(Figs. 3J, 5J, 7J, 9J, 11J, 13J, 15J)

**Type material.** Holotype: ♀, **COSTA RICA:** **Guanacaste:** Pequeña Helvetia, Hotel de los Heroes, 10.475466°−84.830086°, 5.III.2008, Rasplus J.Y. & Ramírez W., ex *Ficus perforata* (= *F. americana* subsp. *americana*) n° JRAS02177_03 (CBGP).

Paratypes: **COSTA RICA:** **Guanacaste:** Pequeña Helvetia, Hotel de los Heroes, 10.475466°−84.830086°, 11♀, 15♂, 5.III.2008, Rasplus J.Y. & Ramírez W., ex *Ficus perforata*(= *F. americana* subsp. *americana*) n° JRAS02177_03 (6♀ 10♂ CBGP, 2♀ 2♂ RPSP, 1♀ 1♂ MZSP, 1♀ 1♂ BMNH, 1♀ 1♂ SAMC).

**Etymology**. The specific name is dedicated to our friend and colleague, Dr. William Ramírez, for his great contribution to the study of fig wasps and figs. The specimens belonging to this species were collected thanks to his valuable help and deep knowledge of the figs of Costa Rica.

**Diagnosis** (♀). Head and mesosoma yellow brown. Mesoscutellum, frenum and axillula smoky yellow to brown. Propodeum yellow. Metasoma brown black. Supraclypeal area wider than torulus. Subantennal groove as long as, or slightly longer than torulus. Distance from torulus to median ocellus 0.9× distance from torulus to oral margin. Metascutellum inconspicuous in dorsal view. Ovipositor sheaths 1.3–1.4× as long as hind tibia.

**Female**.

*Size and colour.* Body length 1.7–1.8 mm. Ovipositor length 0.5 mm. Head and mesosoma yellow brown. Antennae and legs yellow orange. Vertex slightly brown. Mesoscutellum, frenum and axillula slightly brown. Propodeum yellow. Metasoma brown black.

*Head.* Supraclypeal area wider than torulus. Subantennal groove as long as, or slightly longer than torulus. Distance from torulus to median ocellus 0.9× distance from torulus to oral margin. POL 3× OOL. Scape 1.8–2.2× as long as pedicel. Antenna with two anelli. First funicular segment 0.6–0.7× as long as wide, with 5–8 multiporous plate sensilla.

*Mesosoma.* Mesoscutum reticulate to punctate reticulate. Mesoscutum and scutellar-axillar complex not strongly curved in lateral view. Notaulus crenulated. Mesoscutellum 1.2× as long as wide near transscutal articulation. Axillula longitudinally striate to reticulate. Frenal sulcus barely crenulated and inconspicuous. Metascutellum inconspicuous in dorsal view. Anterior margin of propodeum concave medially. Propodeal median line present as a faint longitudinal reticulation. Stigmal vein 0.8× as long as marginal vein, with 3 adstigmal setae. Postmarginal vein nearly absent, shorter than 1/5× stigmal vein length.

*Metasoma.* Ovipositor sheaths 1.3–1.4× as long as hind tibia.

**Male**. Similar to female. Mesosoma paler. Mesoscutellum, frenum, axillula and propodeum yellow. Head smoky yellow to brown. Distance from torulus to median ocellus 1.4× distance from torulus to oral margin. POL 2.1× OOL.

**Host plant**. *Ficus americana* subsp. *americana* Aublet.

### Phylogenetic analyses

Our alignment consisted of 4,024 bp (*COI* = 1, 466 bp; *CytB* = 712 bp; *EF-1α* = 517 bp; *28S rRNA* = 1,329 bp). Protein translations revealed no stop codons or frame shifts. Models chosen by AIC for each partition were GTR + Γ (*mtDNA* & *28S rRNA*), and K80+ Γ (*EF-1α*).

**Figure 16:**
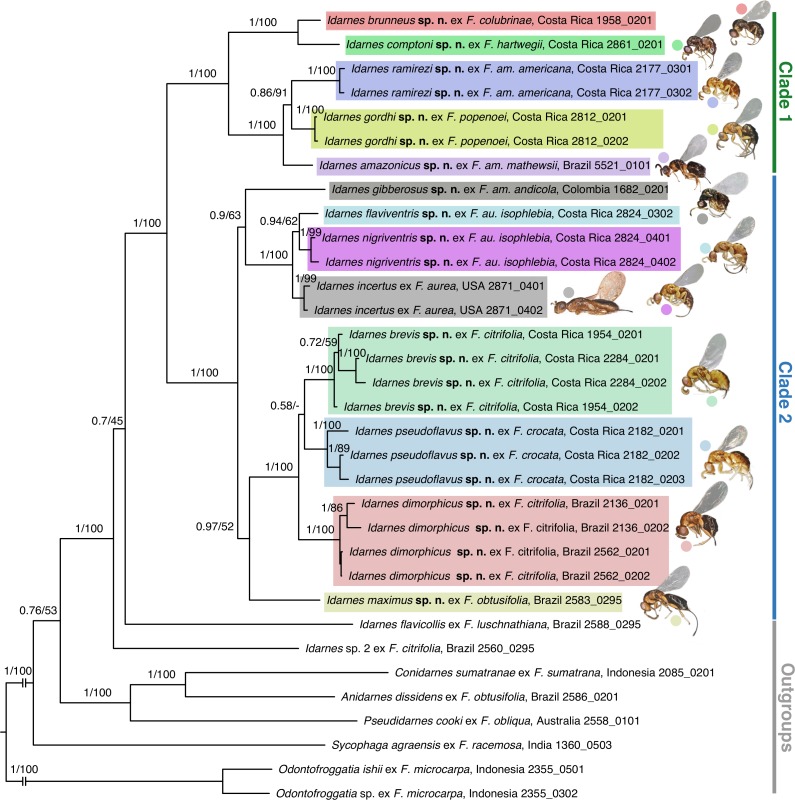
Phylogram of relationships among the *Idarnes incertus* species-group species and eight outgroup species obtained with Bayesian inference. Bayesian posterior probabilities (decimals) and likelihood bootstrap values (percentage) are indicated above nodes (PP/ML_BP_). Boxes indicate specimens belonging to a same species. *F. am.*, *F. americana*; *F. au.*, *F. aurea*.

The trees reconstructed using ML and Bayesian methods showed the same topology (Fig. 16). *Idarnes incertus* species-group was recovered monophyletic (PP = 1; ML_BP_ = 100%) and divided in two main clades (clade 1 and clade 2; Fig. 16). The first clade is well resolved and composed by five species; *I. brunneus* was recovered sister to *I. comptoni* (PP = 1; ML_BP_ = 100%), and *I. amazonicus* was sister to *I. gordhi* plus *I. ramirezi* (PP = 1; ML_BP_ = 100%). The deeper nodes within the second clade were not well resolved, yet we could retrieve a well supported clade formed by *I. pseudoflavus*, *I. brevis*, and *I. dimorphicus* (PP = 1; ML_BP_ = 100%) but the relationships among these tree species were uncertain. Also, *I. incertus* was retrieved as sister to *I. flaviventris* + *I. nigriventris* (PP = 1; ML_BP_ = 100%). The relationships of *I. maximus* and *I. gibberosus* were not well established (Fig. 16). *Idarnes maximus* was recovered sister to *I. brevis* + *I. dimorphicus* + *I. pseudoflavus* with relatively high Bayesian posterior probability support (PP = 0.97) but low maximum likelihood booststrap support (ML_BP_ 52%), while *I. gibberosus* was recovered as sister to the clade *I. flaviventris* + *I. incertus* + *I. nigriventris* with Bayesian posterior probability support (PP = 0.9) yet low ML_BP_ support (ML_BP_ = 63%)

## Discussion

The *Idarnes incertus* species-group is clearly distinct from the remaining *Idarnes*. Indeed, they rarely exhibit metallic tinge and their ovipositor sheaths are always shorter than the body length, whereas the remaining *Idarnes* exhibit metallic colour and the ovipositor is always longer than body. Species belonging to the *I. incertus* species-group are globally similar, and the main differences between species concern the body coloration and the relative length of the ovipositor. According to recent phylogenetic analyses, the *I. incertus* species-group appears to be a recent radiation within Sycophaginae (Cruaud et al., 2011a; Cruaud et al., 2011b) and the morphological similarity of the species may be partly linked to their recent divergence (∼20–10 Ma, during the Miocene; Cruaud et al., 2011a).

Species of the *Idarnes incertus* species-group are usually species-specific with the exception of *I. americanae*, that was found associated with *F. americana* and *F. costaricana* (both species occurring in Costa Rica), and *I. incertus* that is associated with *F. aurea* and *F. citrifolia* in Florida.

Several fig species host more than one species of the *I. incertus* species-group:

1. Four species are associated with *Ficus americana*, namely *I. americanae, I. flavicrus, I. gibberosus*, and *I. ramirezi.* The former two species occurred together within figs of *F. americana* subsp. *americana* in Costa Rica, while *I. ramirezi* occurred in the same subspecies, but in different samples. *Idarnes gibberosus* occurred in figs of *F. americana* subsp. *andicola* in Colombia. *Ficus aurea* hosted three species; the co-occurring *I. flaviventris* and *I. nigriventris* in Costa Rica and *I. incertus* in Florida.
2. *Ficus citrifolia* hosts different species in different parts of its distribution range: *I. dimorphicus* occurs in South America (Brazil: Amazonas, Rondônia, and São Paulo), while *I. brevis* occurs in *F. citrifolia* in Costa Rica, and *I. incertus* in Florida.
3. *Ficus hartwegii* is the host plant of *I. comptoni* and *I. hansoni* in Costa Rica.
4. Finally, *Ficus pertusa* hosts *I. amacayacuensis* and *I. badiovertex.*

These patterns strongly suggest that the diversification of the *I. incertus* species-group within *Ficus* do not follow a “one-to-one rule” of diversification as discussed for pollinators (Rasplus, 1996). Our results clearly show that host shifts between *Ficus* species and diversification on the same *Ficus* host are frequent. Our phylogenetic analyses show one case of diversification within the same host species. Indeed, species associated with the *Ficus aurea* complex (*I. flaviventris* + *I. incertus* + *I. nigriventris*) formed a strongly supported monophyletic clade and were morphologically closely related. On the other hand, species associated with the *F. americana* complex (*I. amazonicus*, *I. gibberosus*, and *I. ramirezi*) belong to different clades, which strongly suggests that host shifts happened. Patterns of diversification within host species were also observed in *Anidarnes* for which sister species occurred on the same host complexes, i.e., *F. aurea* and *F. americana* complexes (Farache et al., 2013). This suggests the existence of different diversification patterns among genera even when they show similar life histories and belong to a same subfamily.

Here we recognized three previously described species belonging to *Idarnes incertus* species-group. Additionally, 17 species new to science are recognized and described. Most species were collected in Costa Rica (11 species), Brazil (4 species) and Colombia (three species). Sampling efforts in Brazil and Costa Rica are comparable, and therefore this shows that the group is probably more diversified in lower latitudes. The high number of new species found in this study highlights the lack of taxonomic information on the Neotropical fig wasps. Despite an increasing number of phylogenetic studies including these wasps (Cruaud et al., 2011a; Cruaud et al., 2010; Cruaud et al., 2011b; Cruaud et al., 2012; Heraty et al., 2013; Munro et al., 2011) just a few recent (i.e., 20th century onwards) taxonomic papers are available on non-pollinating and pollinating wasps (Bouček, 1993; Farache et al., 2013; Jansen-Gonzalez & Sarmiento, 2008; Schiffler, Azevedo & Kawada, 2002; Wiebes, 1995).

This study yields taxonomic and phylogenetic frameworks for a group of *Idarnes*, which represents an important part of the Sycophaginae diversity (ca. 33% of the species). This contribution is an important step to a well-established taxonomic foundation for Agaonidae, and we hope it will subsidize further investigations addressing taxonomy, evolution, and host specificity in fig wasps.

##  Supplemental Information

10.7717/peerj.2842/supp-1 Supplemental Information 1 Lucid key *Idarnes incertus* sp. g Multi-entry taxonomic key for *Idarnes incertus* species-group. The kay is assembled in Lucid: http://www.lucidcentral.com

10.7717/peerj.2842/supp-2 Supplemental Information 2 GenBank assession numbers and sampling information Sampling information and GenBank assession numbers for *Idarnes incertus* sp. gp species and outgroups

10.7717/peerj.2842/supp-3 Supplemental Information 3 MrBayes file and alignment Nexus file with alignment and MrBayes block with parameters for phylogenetic analysis.

10.7717/peerj.2842/supp-4 Supplemental Information 4 Measurements Measurements for *Idarnes incertus* sp. g. species in mm

10.7717/peerj.2842/supp-5 Supplemental Information 5 Species re-descriptions and additional images *Idarnes incertus* sp. gp. Species re-descriptions and additional images.

## Additional Information and Declarations

### Field Study Permissions

The following information was supplied relating to field study approvals (i.e., approving body and any reference numbers): ICMBio provided permissions for material sampling to RASP in Brazil (Permit #1870297).

### DNA Deposition

The following information was supplied regarding the deposition of DNA sequences: Sequences described here are accessible via GenBank accession numbers: COI: HM770634, HM770633, HM770659, HM770620, JQ925920, HM770642, JN001574, KY210168, KY210169, KY210170, KY210171, KY210172, JN001559, JN001560, KY210173, KY210174, KY210175, KY210176, KY210177, HM770643, KY210178, KY210179, KY210180, KY210181, KY210182, HM770626, JN001552, KY210183, HM770625, JN001549; Cytb: HM770588, HM770587, HM770559, HM770576, JQ925902, HM770596, JN001638, KY210184, KY210185, KY210186, KY210187, JN001626, KY210188, KY210189, KY210190, KY210191, KY210192, KY210193, KY210194, KY210195, HM770580, JN001619, KY210196, HM770579, JN001616; EF: HM770537, HM770536, HM770503, HM770522, JQ925887, HM770545, JN001710, KY210197, KY210198, KY210199, KY210200, KY210201, KY210202, KY210203, JN001696, JN001697, KY210204, KY210205, KY210206, KY210207, JN001711, HM770546, KY210208, KY210209, KY210210, KY210211, KY210212, HM770528, JN001689, KY210213, HM770527, JN001686; 28S rRNA: HM770696, KY210146, HM770664, HM770682, JQ925932, HM770704, JN001505, KY210147, KY210148, KY210149, KY210150, KY210151, KY210152, KY210153, JN001503, KY210154, KY210155, KY210156, KY210157, KY210158, KY210159, HM770705, KY210160, KY210161, KY210162, KY210163, KY210164, KY210164, HM770688, KY210165, KY210166, HM770687, KY210167.

### Data Availability

The following information was supplied regarding data availability: The raw data has been supplied as a Supplementary File.

### New Species Registration

The following information was supplied regarding the registration of a newly described species: Publication LSID: urn:lsid:zoobank.org:pub:22286699-8306-4931-8D7F-7BF05EB2B304 genus group: urn:lsid:zoobank.org:act:747168B8-083D-4425-BF1B-A0C4F3B95844 New species: *Idarnes amacayacuensis* Farache & Rasplus, sp. n. urn:lsid:zoobank.org:act:48D01597-E7B0-41AC-8A7E-DCE21AA97EE6 *Idarnes amazonicus* Farache & Rasplus, sp. n. urn:lsid:zoobank.org:act:6F44A1B2-73CC-4267-9F02-AF4E7FF600BC *Idarnes americanae* Farache & Rasplus, sp. n. urn:lsid:zoobank.org:act:0FF58956-AEB0-45C4-8AF9-3BB4C8EF2465 *Idarnes badiovertex* Farache & Rasplus, sp. n. urn:lsid:zoobank.org:act:28673472-103B-4576-A41E-4851E4194771 *Idarnes brevis* Farache & Rasplus, sp. n. urn:lsid:zoobank.org:act:06317A88-E1C5-48AB-83FB-66E79424360C *Idarnes brunneus* Farache & Rasplus, sp. n. urn:lsid:zoobank.org:act:67D1DC74-C6AF-4E66-9897-1DC515D4F253 *Idarnes comptoni* Farache & Rasplus, sp. n. urn:lsid:zoobank.org:act:3D938DAE-2869-40B2-888B-041FC96A7FDB *Idarnes cremersiae* Farache & Rasplus, sp. n. urn:lsid:zoobank.org:act:5F8B227F-7568-4965-BBA4-41C8AEE08EB3 *Idarnes dimorphicus* Farache & Rasplus, sp. n. urn:lsid:zoobank.org:act:6C619C93-7DB1-437B-B4B9-0100C0F3886E *Idarnes flavicrus* Farache & Rasplus, sp. n. urn:lsid:zoobank.org:act:4DFA1180-913B-48E1-B228-1ACE7F4603F0 *Idarnes flaviventris* Farache & Rasplus, sp. n. urn:lsid:zoobank.org:act:8061097A-9783-4A71-8C5B-017C24BD48B5 *Idarnes gordhi* Farache & Rasplus, sp. n. urn:lsid:zoobank.org:act:57B16D6D-A205-4F3A-B53E-E58399809FEC *Idarnes gibberosus* Farache & Rasplus, sp. n. urn:lsid:zoobank.org:act:20EC9435-6547-4FFA-AC77-9205B471F40C *Idarnes maximus* Farache & Rasplus, sp. n. urn:lsid:zoobank.org:act:BA9EEC28-FD78-45B6-953A-11274C64995E *Idarnes nigriventris* Farache & Rasplus, sp. n. urn:lsid:zoobank.org:act:0BE190F5-9E67-45CE-9533-30F65905294E *Idarnes pseudoflavus* Farache & Rasplus, sp. n. urn:lsid:zoobank.org:act:5E9EB253-0BCD-4EA4-A788-0FEED5DE482D *Idarnes ramirezi* Farache & Rasplus, sp. n. urn:lsid:zoobank.org:act:470B1ABF-F6C6-4C8B-BBE5-69ABD5548EA8.
