# Supplementary figures and images for: Taxonomic revision and molecular phylogenetics of the Idarnes incertus species-group (Hymenoptera, Agaonidae, Sycophaginae) (part 2 of 2)
Source: PeerJ. 2017 Jan 5;5:e2842. doi: 10.7717/peerj.2842 (PMC5289451; doi:10.7717/peerj.2842)

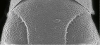

Supplement: Supplemental Information 1 — Multi-entry taxonomic key for Idarnes incertus species-group. The kay is assembled in Lucid: http://www.lucidcentral.com [file peerj-05-2842-s001.zip › Idarnes incertus species group/Media/Thumbs/007_B Mesoscutum without median striae_TN.jpg]

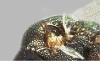

Supplement: Supplemental Information 1 — Multi-entry taxonomic key for Idarnes incertus species-group. The kay is assembled in Lucid: http://www.lucidcentral.com [file peerj-05-2842-s001.zip › Idarnes incertus species group/Media/Thumbs/008_A Mesonotum curved_TN.jpg]

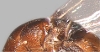

Supplement: Supplemental Information 1 — Multi-entry taxonomic key for Idarnes incertus species-group. The kay is assembled in Lucid: http://www.lucidcentral.com [file peerj-05-2842-s001.zip › Idarnes incertus species group/Media/Thumbs/008_B Mesonotum not strongly curved_TN.jpg]

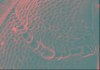

Supplement: Supplemental Information 1 — Multi-entry taxonomic key for Idarnes incertus species-group. The kay is assembled in Lucid: http://www.lucidcentral.com [file peerj-05-2842-s001.zip › Idarnes incertus species group/Media/Thumbs/009_A notaulus crenulate_TN.jpg]

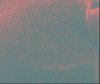

Supplement: Supplemental Information 1 — Multi-entry taxonomic key for Idarnes incertus species-group. The kay is assembled in Lucid: http://www.lucidcentral.com [file peerj-05-2842-s001.zip › Idarnes incertus species group/Media/Thumbs/009_B notaulus not crenulate_TN.jpg]

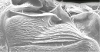

Supplement: Supplemental Information 1 — Multi-entry taxonomic key for Idarnes incertus species-group. The kay is assembled in Lucid: http://www.lucidcentral.com [file peerj-05-2842-s001.zip › Idarnes incertus species group/Media/Thumbs/010_A Axillula striate_TN.jpg]

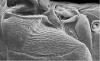

Supplement: Supplemental Information 1 — Multi-entry taxonomic key for Idarnes incertus species-group. The kay is assembled in Lucid: http://www.lucidcentral.com [file peerj-05-2842-s001.zip › Idarnes incertus species group/Media/Thumbs/010_B Axillula reticulate_TN.jpg]

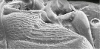

Supplement: Supplemental Information 1 — Multi-entry taxonomic key for Idarnes incertus species-group. The kay is assembled in Lucid: http://www.lucidcentral.com [file peerj-05-2842-s001.zip › Idarnes incertus species group/Media/Thumbs/010_C Axillula striate reticulate_TN.jpg]

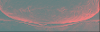

Supplement: Supplemental Information 1 — Multi-entry taxonomic key for Idarnes incertus species-group. The kay is assembled in Lucid: http://www.lucidcentral.com [file peerj-05-2842-s001.zip › Idarnes incertus species group/Media/Thumbs/011_A Frenal sulcus smooth_TN.jpg]

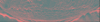

Supplement: Supplemental Information 1 — Multi-entry taxonomic key for Idarnes incertus species-group. The kay is assembled in Lucid: http://www.lucidcentral.com [file peerj-05-2842-s001.zip › Idarnes incertus species group/Media/Thumbs/011_B Frenal barely crenulate_TN.jpg]

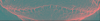

Supplement: Supplemental Information 1 — Multi-entry taxonomic key for Idarnes incertus species-group. The kay is assembled in Lucid: http://www.lucidcentral.com [file peerj-05-2842-s001.zip › Idarnes incertus species group/Media/Thumbs/011_C Frenal sulcus crenulate_TN.jpg]

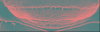

Supplement: Supplemental Information 1 — Multi-entry taxonomic key for Idarnes incertus species-group. The kay is assembled in Lucid: http://www.lucidcentral.com [file peerj-05-2842-s001.zip › Idarnes incertus species group/Media/Thumbs/012_B Frenal sulcus conspicuous_TN.jpg]

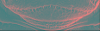

Supplement: Supplemental Information 1 — Multi-entry taxonomic key for Idarnes incertus species-group. The kay is assembled in Lucid: http://www.lucidcentral.com [file peerj-05-2842-s001.zip › Idarnes incertus species group/Media/Thumbs/013_B Frenal sulcus arched_TN.jpg]

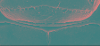

Supplement: Supplemental Information 1 — Multi-entry taxonomic key for Idarnes incertus species-group. The kay is assembled in Lucid: http://www.lucidcentral.com [file peerj-05-2842-s001.zip › Idarnes incertus species group/Media/Thumbs/014_A Metascutellum inconspicuous_TN.jpg]

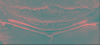

Supplement: Supplemental Information 1 — Multi-entry taxonomic key for Idarnes incertus species-group. The kay is assembled in Lucid: http://www.lucidcentral.com [file peerj-05-2842-s001.zip › Idarnes incertus species group/Media/Thumbs/014_B Metascutellum short_TN.jpg]

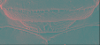

Supplement: Supplemental Information 1 — Multi-entry taxonomic key for Idarnes incertus species-group. The kay is assembled in Lucid: http://www.lucidcentral.com [file peerj-05-2842-s001.zip › Idarnes incertus species group/Media/Thumbs/014_C Metascutellum long_TN.jpg]

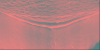

Supplement: Supplemental Information 1 — Multi-entry taxonomic key for Idarnes incertus species-group. The kay is assembled in Lucid: http://www.lucidcentral.com [file peerj-05-2842-s001.zip › Idarnes incertus species group/Media/Thumbs/015_A propodeal margin concave_TN.jpg]

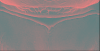

Supplement: Supplemental Information 1 — Multi-entry taxonomic key for Idarnes incertus species-group. The kay is assembled in Lucid: http://www.lucidcentral.com [file peerj-05-2842-s001.zip › Idarnes incertus species group/Media/Thumbs/015_B propodeal margin angulose_TN.jpg]

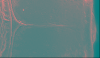

Supplement: Supplemental Information 1 — Multi-entry taxonomic key for Idarnes incertus species-group. The kay is assembled in Lucid: http://www.lucidcentral.com [file peerj-05-2842-s001.zip › Idarnes incertus species group/Media/Thumbs/016_A propodeal sulcus conspicuous_TN.jpg]

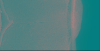

Supplement: Supplemental Information 1 — Multi-entry taxonomic key for Idarnes incertus species-group. The kay is assembled in Lucid: http://www.lucidcentral.com [file peerj-05-2842-s001.zip › Idarnes incertus species group/Media/Thumbs/016_B propodeal sulcus shallow, traceable_TN.jpg]

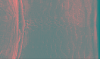

Supplement: Supplemental Information 1 — Multi-entry taxonomic key for Idarnes incertus species-group. The kay is assembled in Lucid: http://www.lucidcentral.com [file peerj-05-2842-s001.zip › Idarnes incertus species group/Media/Thumbs/016_C propodeal sulcus as longit reticulation_TN.jpg]

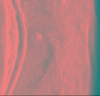

Supplement: Supplemental Information 1 — Multi-entry taxonomic key for Idarnes incertus species-group. The kay is assembled in Lucid: http://www.lucidcentral.com [file peerj-05-2842-s001.zip › Idarnes incertus species group/Media/Thumbs/016_D propodeal sulcus inconspicuous_TN.jpg]

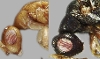

Supplement: Supplemental Information 1 — Multi-entry taxonomic key for Idarnes incertus species-group. The kay is assembled in Lucid: http://www.lucidcentral.com [file peerj-05-2842-s001.zip › Idarnes incertus species group/Media/Thumbs/017_A Pronotum same colour_TN.jpg]

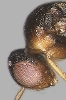

Supplement: Supplemental Information 1 — Multi-entry taxonomic key for Idarnes incertus species-group. The kay is assembled in Lucid: http://www.lucidcentral.com [file peerj-05-2842-s001.zip › Idarnes incertus species group/Media/Thumbs/017_B Pronotum yellow_TN.jpg]

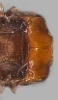

Supplement: Supplemental Information 1 — Multi-entry taxonomic key for Idarnes incertus species-group. The kay is assembled in Lucid: http://www.lucidcentral.com [file peerj-05-2842-s001.zip › Idarnes incertus species group/Media/Thumbs/018_A Propodeum different_TN.jpg]

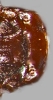

Supplement: Supplemental Information 1 — Multi-entry taxonomic key for Idarnes incertus species-group. The kay is assembled in Lucid: http://www.lucidcentral.com [file peerj-05-2842-s001.zip › Idarnes incertus species group/Media/Thumbs/018_B Propodeum same brown_TN.jpg]

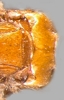

Supplement: Supplemental Information 1 — Multi-entry taxonomic key for Idarnes incertus species-group. The kay is assembled in Lucid: http://www.lucidcentral.com [file peerj-05-2842-s001.zip › Idarnes incertus species group/Media/Thumbs/018_C Propodeum same yellow_TN.jpg]

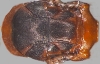

Supplement: Supplemental Information 1 — Multi-entry taxonomic key for Idarnes incertus species-group. The kay is assembled in Lucid: http://www.lucidcentral.com [file peerj-05-2842-s001.zip › Idarnes incertus species group/Media/Thumbs/019_A mes. Dorsally black_TN.jpg]

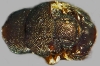

Supplement: Supplemental Information 1 — Multi-entry taxonomic key for Idarnes incertus species-group. The kay is assembled in Lucid: http://www.lucidcentral.com [file peerj-05-2842-s001.zip › Idarnes incertus species group/Media/Thumbs/019_B meso black brown_TN.jpg]

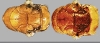

Supplement: Supplemental Information 1 — Multi-entry taxonomic key for Idarnes incertus species-group. The kay is assembled in Lucid: http://www.lucidcentral.com [file peerj-05-2842-s001.zip › Idarnes incertus species group/Media/Thumbs/019_C Mes yellow smoky_TN.jpg]

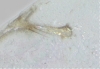

Supplement: Supplemental Information 1 — Multi-entry taxonomic key for Idarnes incertus species-group. The kay is assembled in Lucid: http://www.lucidcentral.com [file peerj-05-2842-s001.zip › Idarnes incertus species group/Media/Thumbs/020_A Pm vein short_TN.jpg]

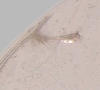

Supplement: Supplemental Information 1 — Multi-entry taxonomic key for Idarnes incertus species-group. The kay is assembled in Lucid: http://www.lucidcentral.com [file peerj-05-2842-s001.zip › Idarnes incertus species group/Media/Thumbs/020_B Pm vein long_TN.jpg]

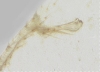

Supplement: Supplemental Information 1 — Multi-entry taxonomic key for Idarnes incertus species-group. The kay is assembled in Lucid: http://www.lucidcentral.com [file peerj-05-2842-s001.zip › Idarnes incertus species group/Media/Thumbs/020_C Pm vein absent_TN.jpg]

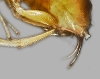

Supplement: Supplemental Information 1 — Multi-entry taxonomic key for Idarnes incertus species-group. The kay is assembled in Lucid: http://www.lucidcentral.com [file peerj-05-2842-s001.zip › Idarnes incertus species group/Media/Thumbs/021_A shorter ovip_TN.jpg]

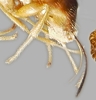

Supplement: Supplemental Information 1 — Multi-entry taxonomic key for Idarnes incertus species-group. The kay is assembled in Lucid: http://www.lucidcentral.com [file peerj-05-2842-s001.zip › Idarnes incertus species group/Media/Thumbs/021_C 1.5x as long as_TN.jpg]

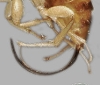

Supplement: Supplemental Information 1 — Multi-entry taxonomic key for Idarnes incertus species-group. The kay is assembled in Lucid: http://www.lucidcentral.com [file peerj-05-2842-s001.zip › Idarnes incertus species group/Media/Thumbs/021_D 2x las lon or more_TN.jpg]

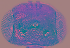

Supplement: Supplemental Information 1 — Multi-entry taxonomic key for Idarnes incertus species-group. The kay is assembled in Lucid: http://www.lucidcentral.com [file peerj-05-2842-s001.zip › Idarnes incertus species group/Media/Thumbs/brown metallic_TN.jpg]

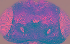

Supplement: Supplemental Information 1 — Multi-entry taxonomic key for Idarnes incertus species-group. The kay is assembled in Lucid: http://www.lucidcentral.com [file peerj-05-2842-s001.zip › Idarnes incertus species group/Media/Thumbs/brown_TN.jpg]

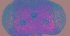

Supplement: Supplemental Information 1 — Multi-entry taxonomic key for Idarnes incertus species-group. The kay is assembled in Lucid: http://www.lucidcentral.com [file peerj-05-2842-s001.zip › Idarnes incertus species group/Media/Thumbs/concolorous_TN.jpg]

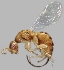

Supplement: Supplemental Information 1 — Multi-entry taxonomic key for Idarnes incertus species-group. The kay is assembled in Lucid: http://www.lucidcentral.com [file peerj-05-2842-s001.zip › Idarnes incertus species group/Media/Thumbs/hab_albiventris_TN.jpg]

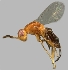

Supplement: Supplemental Information 1 — Multi-entry taxonomic key for Idarnes incertus species-group. The kay is assembled in Lucid: http://www.lucidcentral.com [file peerj-05-2842-s001.zip › Idarnes incertus species group/Media/Thumbs/hab_amacayacuensis_TN.jpg]

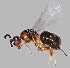

Supplement: Supplemental Information 1 — Multi-entry taxonomic key for Idarnes incertus species-group. The kay is assembled in Lucid: http://www.lucidcentral.com [file peerj-05-2842-s001.zip › Idarnes incertus species group/Media/Thumbs/hab_amazonicus_TN.jpg]

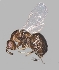

Supplement: Supplemental Information 1 — Multi-entry taxonomic key for Idarnes incertus species-group. The kay is assembled in Lucid: http://www.lucidcentral.com [file peerj-05-2842-s001.zip › Idarnes incertus species group/Media/Thumbs/hab_americanae_TN.jpg]

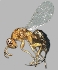

Supplement: Supplemental Information 1 — Multi-entry taxonomic key for Idarnes incertus species-group. The kay is assembled in Lucid: http://www.lucidcentral.com [file peerj-05-2842-s001.zip › Idarnes incertus species group/Media/Thumbs/hab_aureonigrus_TN.jpg]

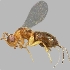

Supplement: Supplemental Information 1 — Multi-entry taxonomic key for Idarnes incertus species-group. The kay is assembled in Lucid: http://www.lucidcentral.com [file peerj-05-2842-s001.zip › Idarnes incertus species group/Media/Thumbs/hab_badiovertex_TN.jpg]

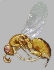

Supplement: Supplemental Information 1 — Multi-entry taxonomic key for Idarnes incertus species-group. The kay is assembled in Lucid: http://www.lucidcentral.com [file peerj-05-2842-s001.zip › Idarnes incertus species group/Media/Thumbs/hab_brevis_TN.jpg]

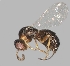

Supplement: Supplemental Information 1 — Multi-entry taxonomic key for Idarnes incertus species-group. The kay is assembled in Lucid: http://www.lucidcentral.com [file peerj-05-2842-s001.zip › Idarnes incertus species group/Media/Thumbs/hab_brunneus_TN.jpg]

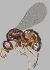

Supplement: Supplemental Information 1 — Multi-entry taxonomic key for Idarnes incertus species-group. The kay is assembled in Lucid: http://www.lucidcentral.com [file peerj-05-2842-s001.zip › Idarnes incertus species group/Media/Thumbs/hab_comptoni_TN.jpg]

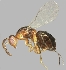

Supplement: Supplemental Information 1 — Multi-entry taxonomic key for Idarnes incertus species-group. The kay is assembled in Lucid: http://www.lucidcentral.com [file peerj-05-2842-s001.zip › Idarnes incertus species group/Media/Thumbs/hab_cremersae_TN.jpg]

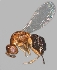

Supplement: Supplemental Information 1 — Multi-entry taxonomic key for Idarnes incertus species-group. The kay is assembled in Lucid: http://www.lucidcentral.com [file peerj-05-2842-s001.zip › Idarnes incertus species group/Media/Thumbs/hab_dimorphicus_TN.jpg]

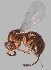

Supplement: Supplemental Information 1 — Multi-entry taxonomic key for Idarnes incertus species-group. The kay is assembled in Lucid: http://www.lucidcentral.com [file peerj-05-2842-s001.zip › Idarnes incertus species group/Media/Thumbs/hab_flavicrus_TN.jpg]

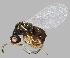

Supplement: Supplemental Information 1 — Multi-entry taxonomic key for Idarnes incertus species-group. The kay is assembled in Lucid: http://www.lucidcentral.com [file peerj-05-2842-s001.zip › Idarnes incertus species group/Media/Thumbs/hab_gibberosus_TN.jpg]

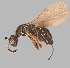

Supplement: Supplemental Information 1 — Multi-entry taxonomic key for Idarnes incertus species-group. The kay is assembled in Lucid: http://www.lucidcentral.com [file peerj-05-2842-s001.zip › Idarnes incertus species group/Media/Thumbs/hab_hansoni_TN.jpg]

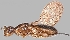

Supplement: Supplemental Information 1 — Multi-entry taxonomic key for Idarnes incertus species-group. The kay is assembled in Lucid: http://www.lucidcentral.com [file peerj-05-2842-s001.zip › Idarnes incertus species group/Media/Thumbs/hab_incertus_TN.jpg]

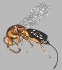

Supplement: Supplemental Information 1 — Multi-entry taxonomic key for Idarnes incertus species-group. The kay is assembled in Lucid: http://www.lucidcentral.com [file peerj-05-2842-s001.zip › Idarnes incertus species group/Media/Thumbs/hab_maximus_TN.jpg]

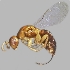

Supplement: Supplemental Information 1 — Multi-entry taxonomic key for Idarnes incertus species-group. The kay is assembled in Lucid: http://www.lucidcentral.com [file peerj-05-2842-s001.zip › Idarnes incertus species group/Media/Thumbs/hab_nigriventris_TN.jpg]

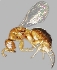

Supplement: Supplemental Information 1 — Multi-entry taxonomic key for Idarnes incertus species-group. The kay is assembled in Lucid: http://www.lucidcentral.com [file peerj-05-2842-s001.zip › Idarnes incertus species group/Media/Thumbs/hab_pseudoflavus_TN.jpg]

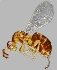

Supplement: Supplemental Information 1 — Multi-entry taxonomic key for Idarnes incertus species-group. The kay is assembled in Lucid: http://www.lucidcentral.com [file peerj-05-2842-s001.zip › Idarnes incertus species group/Media/Thumbs/hab_williamsi_TN.jpg]

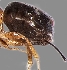

Supplement: Supplemental Information 1 — Multi-entry taxonomic key for Idarnes incertus species-group. The kay is assembled in Lucid: http://www.lucidcentral.com [file peerj-05-2842-s001.zip › Idarnes incertus species group/Media/Thumbs/mesosoma dark_TN.jpg]

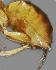

Supplement: Supplemental Information 1 — Multi-entry taxonomic key for Idarnes incertus species-group. The kay is assembled in Lucid: http://www.lucidcentral.com [file peerj-05-2842-s001.zip › Idarnes incertus species group/Media/Thumbs/mesosoma yellow_TN.jpg]

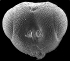

Supplement: Supplemental Information 1 — Multi-entry taxonomic key for Idarnes incertus species-group. The kay is assembled in Lucid: http://www.lucidcentral.com [file peerj-05-2842-s001.zip › Idarnes incertus species group/Media/Thumbs/torulus middle_TN.jpg]
