# Supplementary figures and images for: Taxonomic revision and molecular phylogenetics of the Idarnes incertus species-group (Hymenoptera, Agaonidae, Sycophaginae) (part 1 of 2)
Source: PeerJ. 2017 Jan 5;5:e2842. doi: 10.7717/peerj.2842 (PMC5289451; doi:10.7717/peerj.2842)

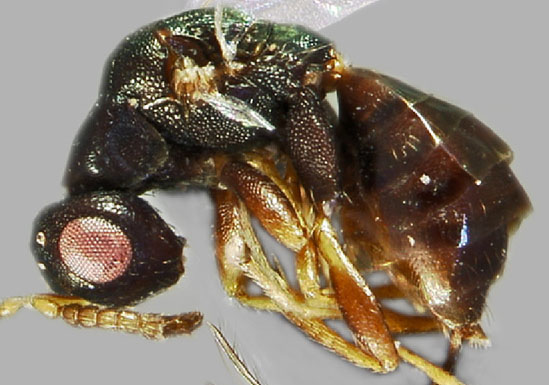

Supplement: Supplemental Information 1 — Multi-entry taxonomic key for Idarnes incertus species-group. The kay is assembled in Lucid: http://www.lucidcentral.com [file peerj-05-2842-s001.zip › Idarnes incertus species group/Media/Images/001_A Body black.jpg]

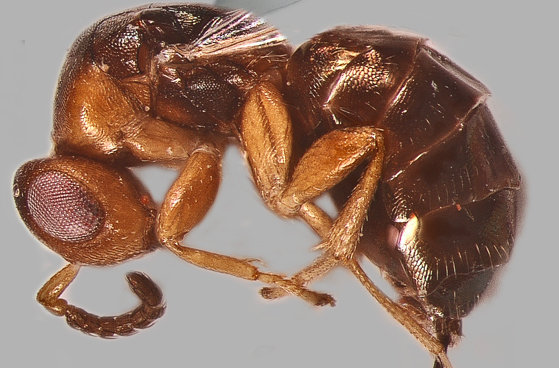

Supplement: Supplemental Information 1 — Multi-entry taxonomic key for Idarnes incertus species-group. The kay is assembled in Lucid: http://www.lucidcentral.com [file peerj-05-2842-s001.zip › Idarnes incertus species group/Media/Images/001_B_Body brown yellow.jpg]

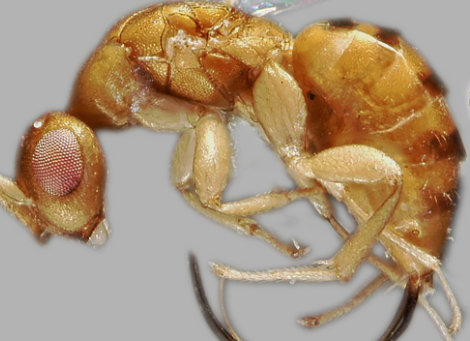

Supplement: Supplemental Information 1 — Multi-entry taxonomic key for Idarnes incertus species-group. The kay is assembled in Lucid: http://www.lucidcentral.com [file peerj-05-2842-s001.zip › Idarnes incertus species group/Media/Images/001_C Body yellow.jpg]

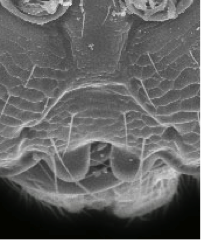

Supplement: Supplemental Information 1 — Multi-entry taxonomic key for Idarnes incertus species-group. The kay is assembled in Lucid: http://www.lucidcentral.com [file peerj-05-2842-s001.zip › Idarnes incertus species group/Media/Images/002_A Epistomal delim clypeus.png]

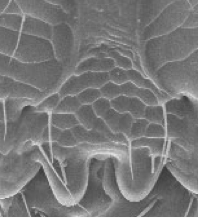

Supplement: Supplemental Information 1 — Multi-entry taxonomic key for Idarnes incertus species-group. The kay is assembled in Lucid: http://www.lucidcentral.com [file peerj-05-2842-s001.zip › Idarnes incertus species group/Media/Images/002_B Epistomal Shallow.png]

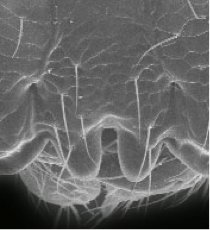

Supplement: Supplemental Information 1 — Multi-entry taxonomic key for Idarnes incertus species-group. The kay is assembled in Lucid: http://www.lucidcentral.com [file peerj-05-2842-s001.zip › Idarnes incertus species group/Media/Images/002_C Epistomal inconspicuous.png]

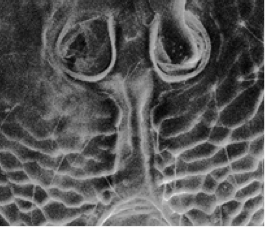

Supplement: Supplemental Information 1 — Multi-entry taxonomic key for Idarnes incertus species-group. The kay is assembled in Lucid: http://www.lucidcentral.com [file peerj-05-2842-s001.zip › Idarnes incertus species group/Media/Images/003_A Supraclyp narrow.png]

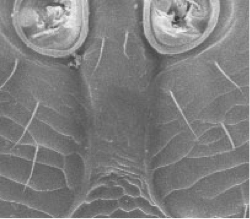

Supplement: Supplemental Information 1 — Multi-entry taxonomic key for Idarnes incertus species-group. The kay is assembled in Lucid: http://www.lucidcentral.com [file peerj-05-2842-s001.zip › Idarnes incertus species group/Media/Images/003_B Supraclyp as wide.png]

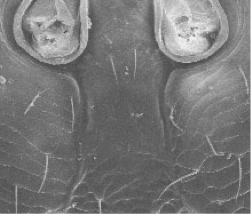

Supplement: Supplemental Information 1 — Multi-entry taxonomic key for Idarnes incertus species-group. The kay is assembled in Lucid: http://www.lucidcentral.com [file peerj-05-2842-s001.zip › Idarnes incertus species group/Media/Images/003_C Supraclyp wider.png]

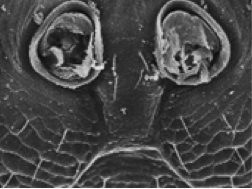

Supplement: Supplemental Information 1 — Multi-entry taxonomic key for Idarnes incertus species-group. The kay is assembled in Lucid: http://www.lucidcentral.com [file peerj-05-2842-s001.zip › Idarnes incertus species group/Media/Images/004_A Subantennal short.png]

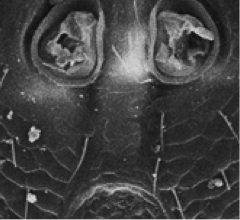

Supplement: Supplemental Information 1 — Multi-entry taxonomic key for Idarnes incertus species-group. The kay is assembled in Lucid: http://www.lucidcentral.com [file peerj-05-2842-s001.zip › Idarnes incertus species group/Media/Images/004_B Subantennal as long.png]

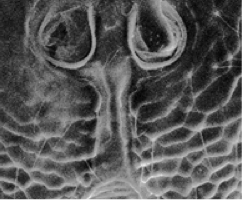

Supplement: Supplemental Information 1 — Multi-entry taxonomic key for Idarnes incertus species-group. The kay is assembled in Lucid: http://www.lucidcentral.com [file peerj-05-2842-s001.zip › Idarnes incertus species group/Media/Images/004_C Subantennal longer.png]

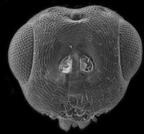

Supplement: Supplemental Information 1 — Multi-entry taxonomic key for Idarnes incertus species-group. The kay is assembled in Lucid: http://www.lucidcentral.com [file peerj-05-2842-s001.zip › Idarnes incertus species group/Media/Images/005_A Torulus closer ocellus.png]

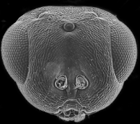

Supplement: Supplemental Information 1 — Multi-entry taxonomic key for Idarnes incertus species-group. The kay is assembled in Lucid: http://www.lucidcentral.com [file peerj-05-2842-s001.zip › Idarnes incertus species group/Media/Images/005_B Torulus closer oral margin.png]

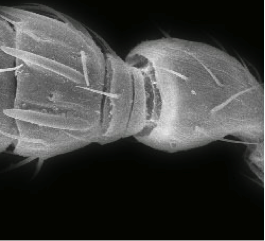

Supplement: Supplemental Information 1 — Multi-entry taxonomic key for Idarnes incertus species-group. The kay is assembled in Lucid: http://www.lucidcentral.com [file peerj-05-2842-s001.zip › Idarnes incertus species group/Media/Images/006_A funicle 2 anelli.png]

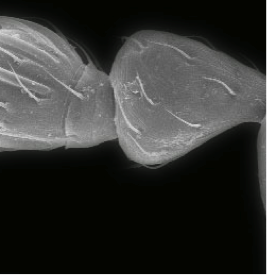

Supplement: Supplemental Information 1 — Multi-entry taxonomic key for Idarnes incertus species-group. The kay is assembled in Lucid: http://www.lucidcentral.com [file peerj-05-2842-s001.zip › Idarnes incertus species group/Media/Images/006_B funicle 2 anelli (nearly fused).png]

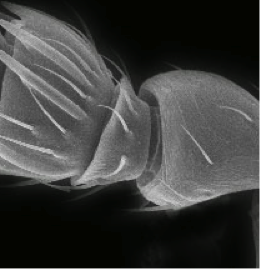

Supplement: Supplemental Information 1 — Multi-entry taxonomic key for Idarnes incertus species-group. The kay is assembled in Lucid: http://www.lucidcentral.com [file peerj-05-2842-s001.zip › Idarnes incertus species group/Media/Images/006_C funicle 1 anellus.png]

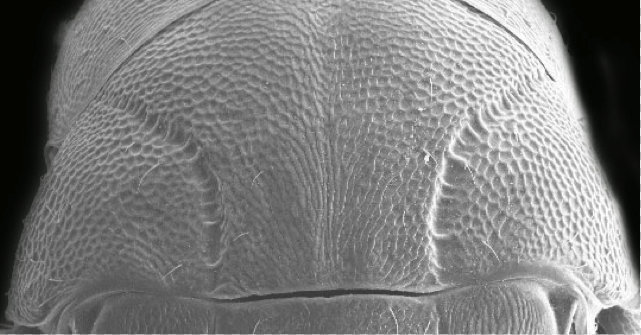

Supplement: Supplemental Information 1 — Multi-entry taxonomic key for Idarnes incertus species-group. The kay is assembled in Lucid: http://www.lucidcentral.com [file peerj-05-2842-s001.zip › Idarnes incertus species group/Media/Images/007_A Mesoscutum with median striae.jpg]

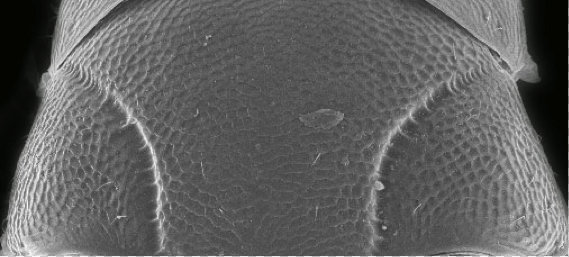

Supplement: Supplemental Information 1 — Multi-entry taxonomic key for Idarnes incertus species-group. The kay is assembled in Lucid: http://www.lucidcentral.com [file peerj-05-2842-s001.zip › Idarnes incertus species group/Media/Images/007_B Mesoscutum without median striae.jpg]

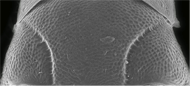

Supplement: Supplemental Information 1 — Multi-entry taxonomic key for Idarnes incertus species-group. The kay is assembled in Lucid: http://www.lucidcentral.com [file peerj-05-2842-s001.zip › Idarnes incertus species group/Media/Images/007_B Mesoscutum without median striae.png]

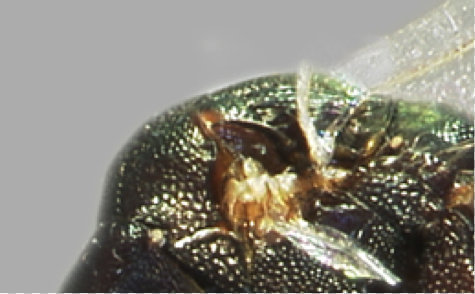

Supplement: Supplemental Information 1 — Multi-entry taxonomic key for Idarnes incertus species-group. The kay is assembled in Lucid: http://www.lucidcentral.com [file peerj-05-2842-s001.zip › Idarnes incertus species group/Media/Images/008_A Mesonotum curved.jpg]

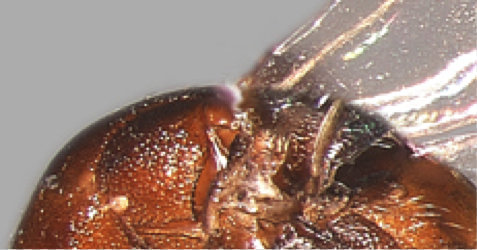

Supplement: Supplemental Information 1 — Multi-entry taxonomic key for Idarnes incertus species-group. The kay is assembled in Lucid: http://www.lucidcentral.com [file peerj-05-2842-s001.zip › Idarnes incertus species group/Media/Images/008_B Mesonotum not strongly curved.jpg]

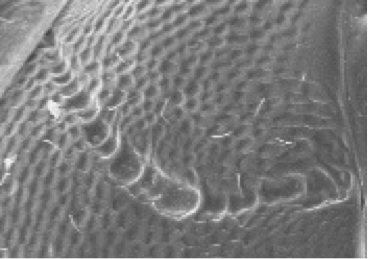

Supplement: Supplemental Information 1 — Multi-entry taxonomic key for Idarnes incertus species-group. The kay is assembled in Lucid: http://www.lucidcentral.com [file peerj-05-2842-s001.zip › Idarnes incertus species group/Media/Images/009_A notaulus crenulate.png]

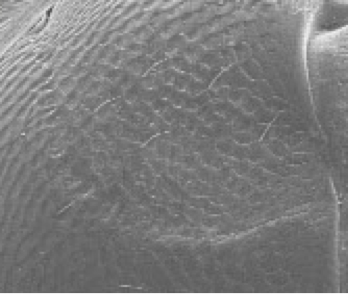

Supplement: Supplemental Information 1 — Multi-entry taxonomic key for Idarnes incertus species-group. The kay is assembled in Lucid: http://www.lucidcentral.com [file peerj-05-2842-s001.zip › Idarnes incertus species group/Media/Images/009_B notaulus not crenulate.png]

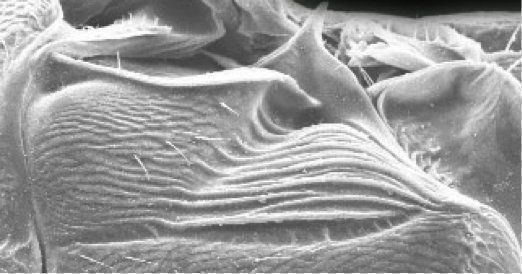

Supplement: Supplemental Information 1 — Multi-entry taxonomic key for Idarnes incertus species-group. The kay is assembled in Lucid: http://www.lucidcentral.com [file peerj-05-2842-s001.zip › Idarnes incertus species group/Media/Images/010_A Axillula striate.jpg]

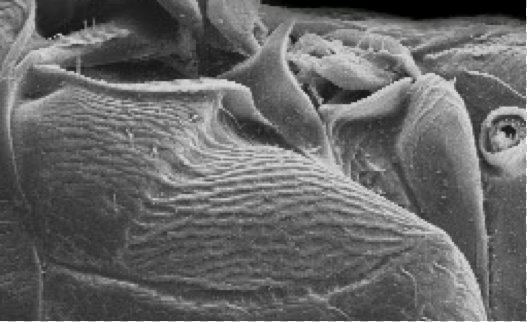

Supplement: Supplemental Information 1 — Multi-entry taxonomic key for Idarnes incertus species-group. The kay is assembled in Lucid: http://www.lucidcentral.com [file peerj-05-2842-s001.zip › Idarnes incertus species group/Media/Images/010_B Axillula reticulate.jpg]

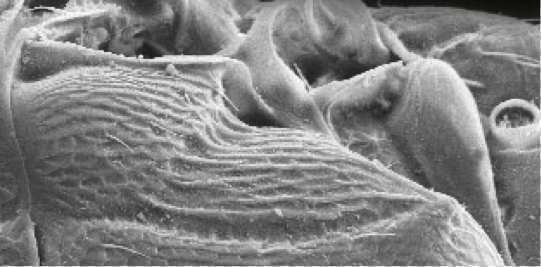

Supplement: Supplemental Information 1 — Multi-entry taxonomic key for Idarnes incertus species-group. The kay is assembled in Lucid: http://www.lucidcentral.com [file peerj-05-2842-s001.zip › Idarnes incertus species group/Media/Images/010_C Axillula striate reticulate.jpg]

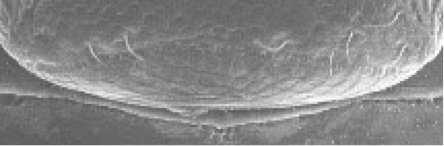

Supplement: Supplemental Information 1 — Multi-entry taxonomic key for Idarnes incertus species-group. The kay is assembled in Lucid: http://www.lucidcentral.com [file peerj-05-2842-s001.zip › Idarnes incertus species group/Media/Images/011_A Frenal sulcus smooth.png]

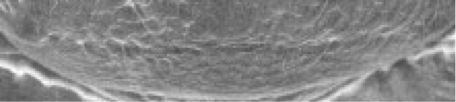

Supplement: Supplemental Information 1 — Multi-entry taxonomic key for Idarnes incertus species-group. The kay is assembled in Lucid: http://www.lucidcentral.com [file peerj-05-2842-s001.zip › Idarnes incertus species group/Media/Images/011_B Frenal barely crenulate.png]

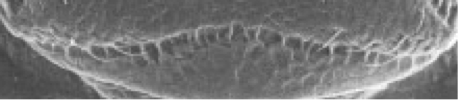

Supplement: Supplemental Information 1 — Multi-entry taxonomic key for Idarnes incertus species-group. The kay is assembled in Lucid: http://www.lucidcentral.com [file peerj-05-2842-s001.zip › Idarnes incertus species group/Media/Images/011_C Frenal sulcus crenulate.png]

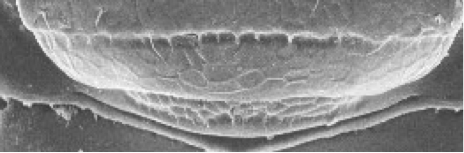

Supplement: Supplemental Information 1 — Multi-entry taxonomic key for Idarnes incertus species-group. The kay is assembled in Lucid: http://www.lucidcentral.com [file peerj-05-2842-s001.zip › Idarnes incertus species group/Media/Images/012_B Frenal sulcus conspicuous.png]

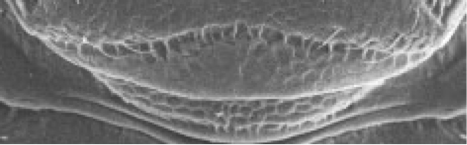

Supplement: Supplemental Information 1 — Multi-entry taxonomic key for Idarnes incertus species-group. The kay is assembled in Lucid: http://www.lucidcentral.com [file peerj-05-2842-s001.zip › Idarnes incertus species group/Media/Images/013_B Frenal sulcus arched.png]

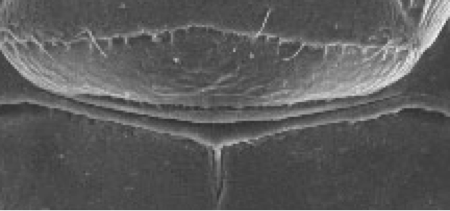

Supplement: Supplemental Information 1 — Multi-entry taxonomic key for Idarnes incertus species-group. The kay is assembled in Lucid: http://www.lucidcentral.com [file peerj-05-2842-s001.zip › Idarnes incertus species group/Media/Images/014_A Metascutellum inconspicuous.png]

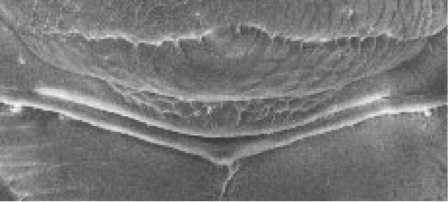

Supplement: Supplemental Information 1 — Multi-entry taxonomic key for Idarnes incertus species-group. The kay is assembled in Lucid: http://www.lucidcentral.com [file peerj-05-2842-s001.zip › Idarnes incertus species group/Media/Images/014_B Metascutellum short.png]

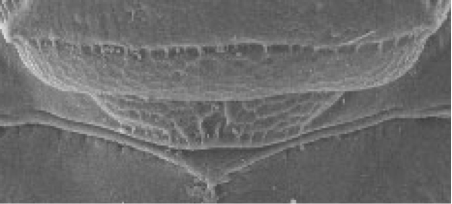

Supplement: Supplemental Information 1 — Multi-entry taxonomic key for Idarnes incertus species-group. The kay is assembled in Lucid: http://www.lucidcentral.com [file peerj-05-2842-s001.zip › Idarnes incertus species group/Media/Images/014_C Metascutellum long.png]

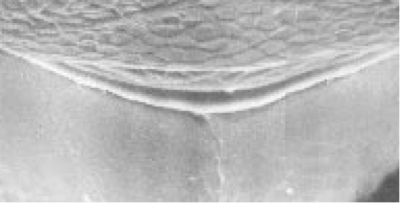

Supplement: Supplemental Information 1 — Multi-entry taxonomic key for Idarnes incertus species-group. The kay is assembled in Lucid: http://www.lucidcentral.com [file peerj-05-2842-s001.zip › Idarnes incertus species group/Media/Images/015_A propodeal margin concave.png]

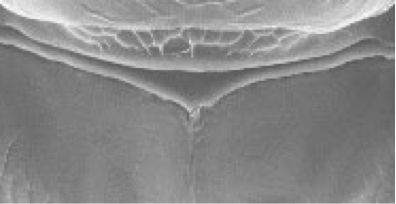

Supplement: Supplemental Information 1 — Multi-entry taxonomic key for Idarnes incertus species-group. The kay is assembled in Lucid: http://www.lucidcentral.com [file peerj-05-2842-s001.zip › Idarnes incertus species group/Media/Images/015_B propodeal margin angulose.png]

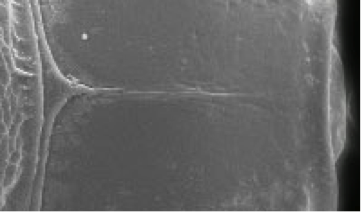

Supplement: Supplemental Information 1 — Multi-entry taxonomic key for Idarnes incertus species-group. The kay is assembled in Lucid: http://www.lucidcentral.com [file peerj-05-2842-s001.zip › Idarnes incertus species group/Media/Images/016_A propodeal sulcus conspicuous.png]

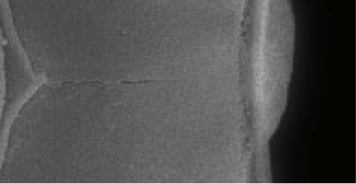

Supplement: Supplemental Information 1 — Multi-entry taxonomic key for Idarnes incertus species-group. The kay is assembled in Lucid: http://www.lucidcentral.com [file peerj-05-2842-s001.zip › Idarnes incertus species group/Media/Images/016_B propodeal sulcus shallow, traceable.png]

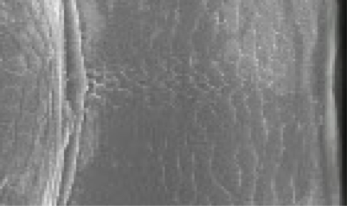

Supplement: Supplemental Information 1 — Multi-entry taxonomic key for Idarnes incertus species-group. The kay is assembled in Lucid: http://www.lucidcentral.com [file peerj-05-2842-s001.zip › Idarnes incertus species group/Media/Images/016_C propodeal sulcus as longit reticulation.png]

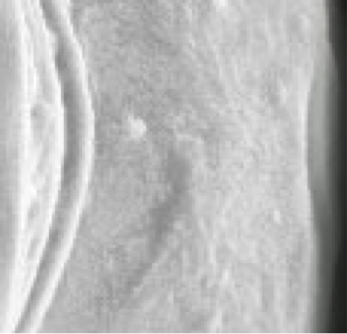

Supplement: Supplemental Information 1 — Multi-entry taxonomic key for Idarnes incertus species-group. The kay is assembled in Lucid: http://www.lucidcentral.com [file peerj-05-2842-s001.zip › Idarnes incertus species group/Media/Images/016_D propodeal sulcus inconspicuous.png]

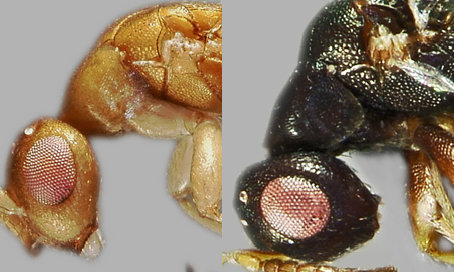

Supplement: Supplemental Information 1 — Multi-entry taxonomic key for Idarnes incertus species-group. The kay is assembled in Lucid: http://www.lucidcentral.com [file peerj-05-2842-s001.zip › Idarnes incertus species group/Media/Images/017_A Pronotum same colour.jpg]

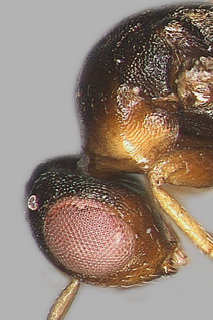

Supplement: Supplemental Information 1 — Multi-entry taxonomic key for Idarnes incertus species-group. The kay is assembled in Lucid: http://www.lucidcentral.com [file peerj-05-2842-s001.zip › Idarnes incertus species group/Media/Images/017_B Pronotum yellow.jpg]

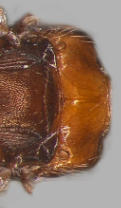

Supplement: Supplemental Information 1 — Multi-entry taxonomic key for Idarnes incertus species-group. The kay is assembled in Lucid: http://www.lucidcentral.com [file peerj-05-2842-s001.zip › Idarnes incertus species group/Media/Images/018_A Propodeum different.png]

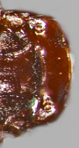

Supplement: Supplemental Information 1 — Multi-entry taxonomic key for Idarnes incertus species-group. The kay is assembled in Lucid: http://www.lucidcentral.com [file peerj-05-2842-s001.zip › Idarnes incertus species group/Media/Images/018_B Propodeum same brown.png]

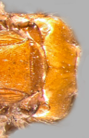

Supplement: Supplemental Information 1 — Multi-entry taxonomic key for Idarnes incertus species-group. The kay is assembled in Lucid: http://www.lucidcentral.com [file peerj-05-2842-s001.zip › Idarnes incertus species group/Media/Images/018_C Propodeum same yellow.png]

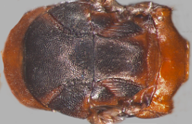

Supplement: Supplemental Information 1 — Multi-entry taxonomic key for Idarnes incertus species-group. The kay is assembled in Lucid: http://www.lucidcentral.com [file peerj-05-2842-s001.zip › Idarnes incertus species group/Media/Images/019_A mes. Dorsally black.png]

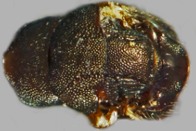

Supplement: Supplemental Information 1 — Multi-entry taxonomic key for Idarnes incertus species-group. The kay is assembled in Lucid: http://www.lucidcentral.com [file peerj-05-2842-s001.zip › Idarnes incertus species group/Media/Images/019_B meso black brown.png]

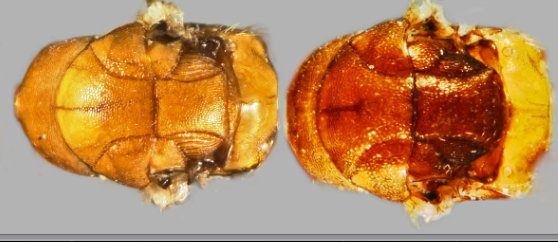

Supplement: Supplemental Information 1 — Multi-entry taxonomic key for Idarnes incertus species-group. The kay is assembled in Lucid: http://www.lucidcentral.com [file peerj-05-2842-s001.zip › Idarnes incertus species group/Media/Images/019_C Mes yellow smoky.jpg]

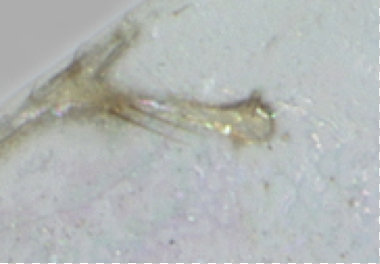

Supplement: Supplemental Information 1 — Multi-entry taxonomic key for Idarnes incertus species-group. The kay is assembled in Lucid: http://www.lucidcentral.com [file peerj-05-2842-s001.zip › Idarnes incertus species group/Media/Images/020_A Pm vein short.jpg]

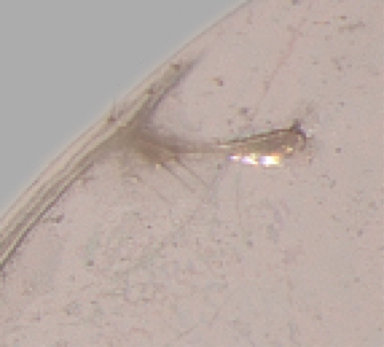

Supplement: Supplemental Information 1 — Multi-entry taxonomic key for Idarnes incertus species-group. The kay is assembled in Lucid: http://www.lucidcentral.com [file peerj-05-2842-s001.zip › Idarnes incertus species group/Media/Images/020_B Pm vein long.jpg]

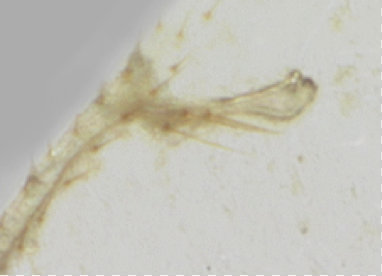

Supplement: Supplemental Information 1 — Multi-entry taxonomic key for Idarnes incertus species-group. The kay is assembled in Lucid: http://www.lucidcentral.com [file peerj-05-2842-s001.zip › Idarnes incertus species group/Media/Images/020_C Pm vein absent.jpg]

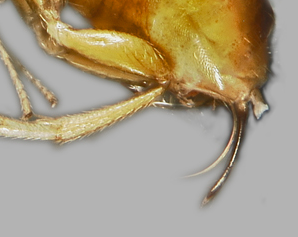

Supplement: Supplemental Information 1 — Multi-entry taxonomic key for Idarnes incertus species-group. The kay is assembled in Lucid: http://www.lucidcentral.com [file peerj-05-2842-s001.zip › Idarnes incertus species group/Media/Images/021_A shorter ovip.png]

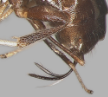

Supplement: Supplemental Information 1 — Multi-entry taxonomic key for Idarnes incertus species-group. The kay is assembled in Lucid: http://www.lucidcentral.com [file peerj-05-2842-s001.zip › Idarnes incertus species group/Media/Images/021_BA~1.PNG]

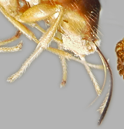

Supplement: Supplemental Information 1 — Multi-entry taxonomic key for Idarnes incertus species-group. The kay is assembled in Lucid: http://www.lucidcentral.com [file peerj-05-2842-s001.zip › Idarnes incertus species group/Media/Images/021_C 1.5x as long as.png]

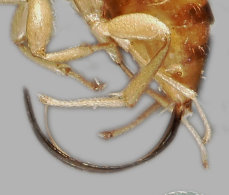

Supplement: Supplemental Information 1 — Multi-entry taxonomic key for Idarnes incertus species-group. The kay is assembled in Lucid: http://www.lucidcentral.com [file peerj-05-2842-s001.zip › Idarnes incertus species group/Media/Images/021_D 2x las lon or more.jpg]

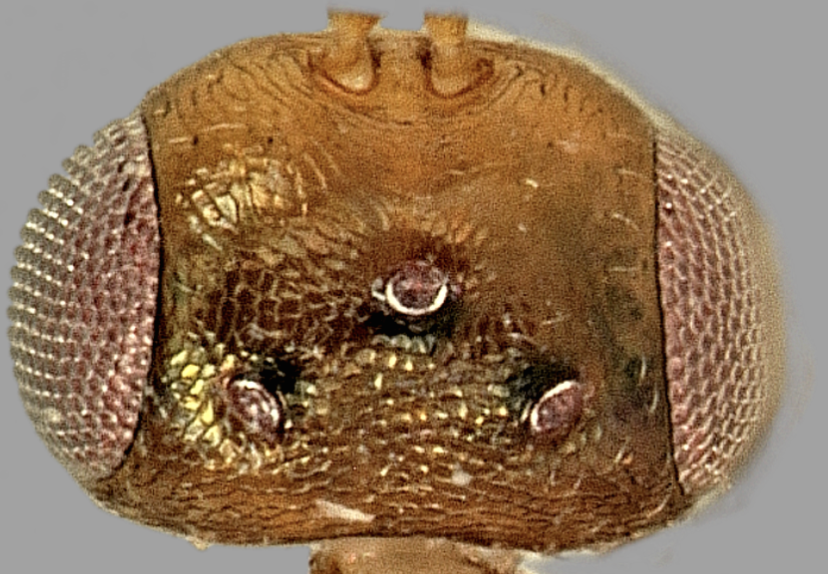

Supplement: Supplemental Information 1 — Multi-entry taxonomic key for Idarnes incertus species-group. The kay is assembled in Lucid: http://www.lucidcentral.com [file peerj-05-2842-s001.zip › Idarnes incertus species group/Media/Images/brown metallic.png]

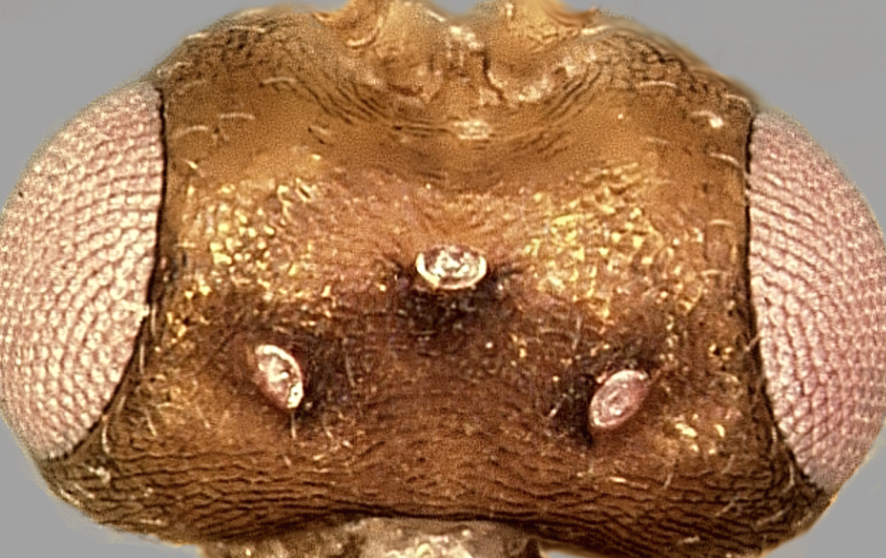

Supplement: Supplemental Information 1 — Multi-entry taxonomic key for Idarnes incertus species-group. The kay is assembled in Lucid: http://www.lucidcentral.com [file peerj-05-2842-s001.zip › Idarnes incertus species group/Media/Images/brown.png]

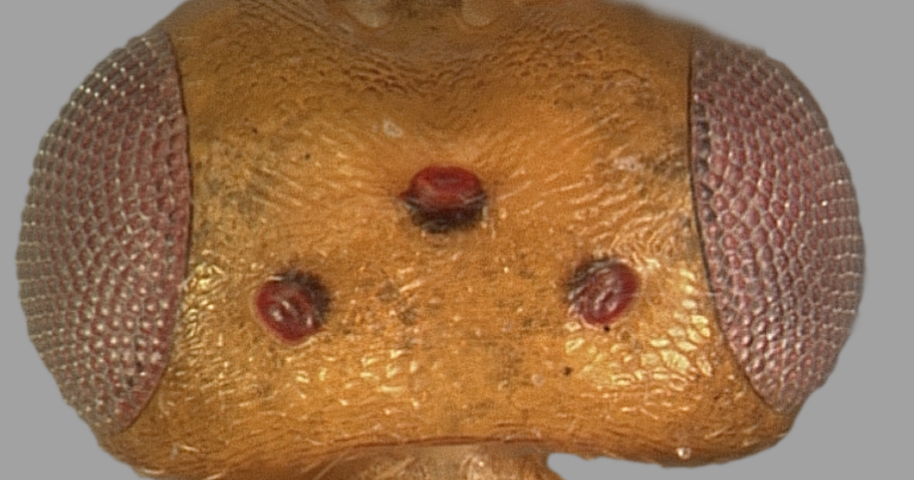

Supplement: Supplemental Information 1 — Multi-entry taxonomic key for Idarnes incertus species-group. The kay is assembled in Lucid: http://www.lucidcentral.com [file peerj-05-2842-s001.zip › Idarnes incertus species group/Media/Images/concolorous.png]

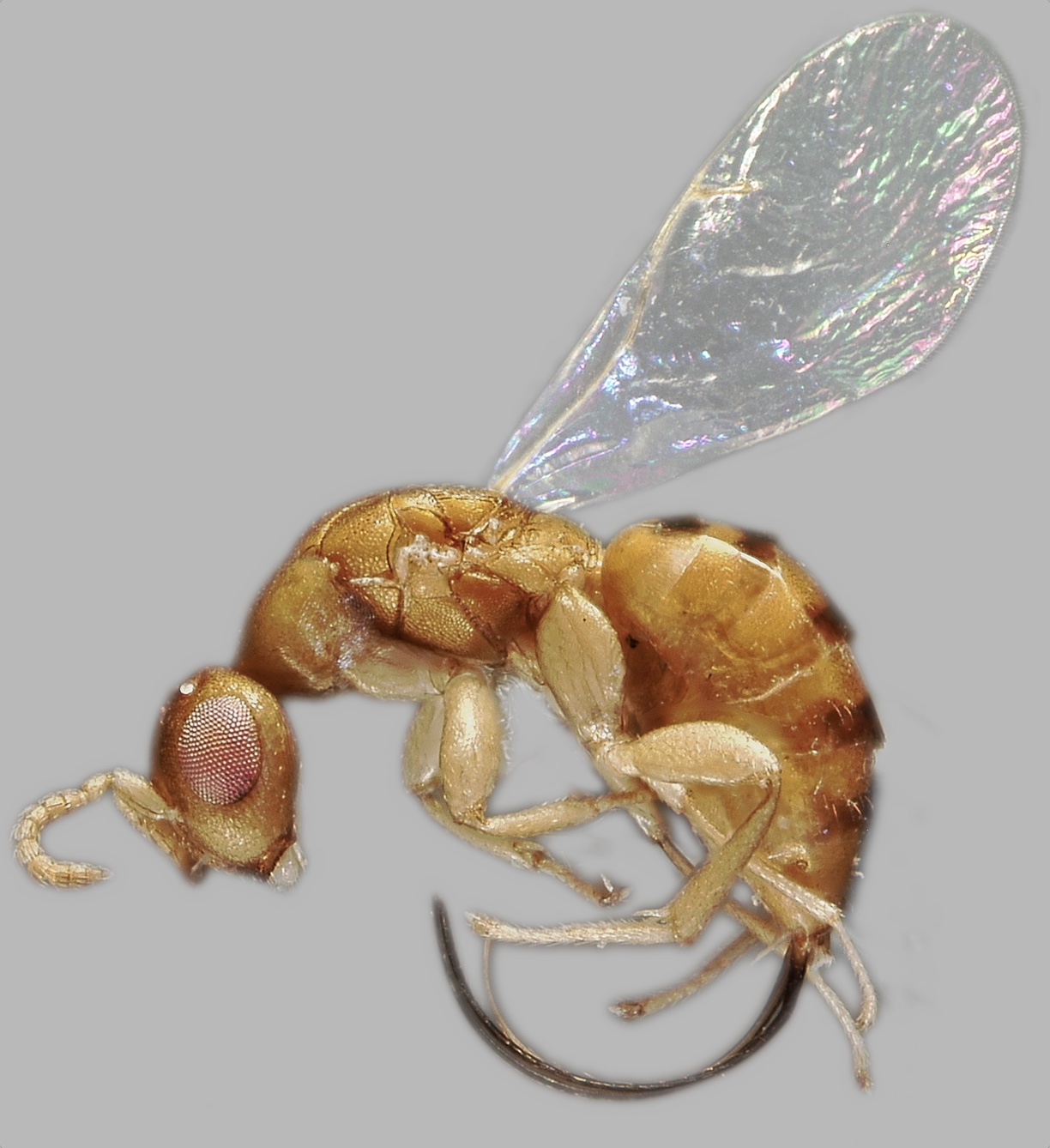

Supplement: Supplemental Information 1 — Multi-entry taxonomic key for Idarnes incertus species-group. The kay is assembled in Lucid: http://www.lucidcentral.com [file peerj-05-2842-s001.zip › Idarnes incertus species group/Media/Images/hab_albiventris.jpg]

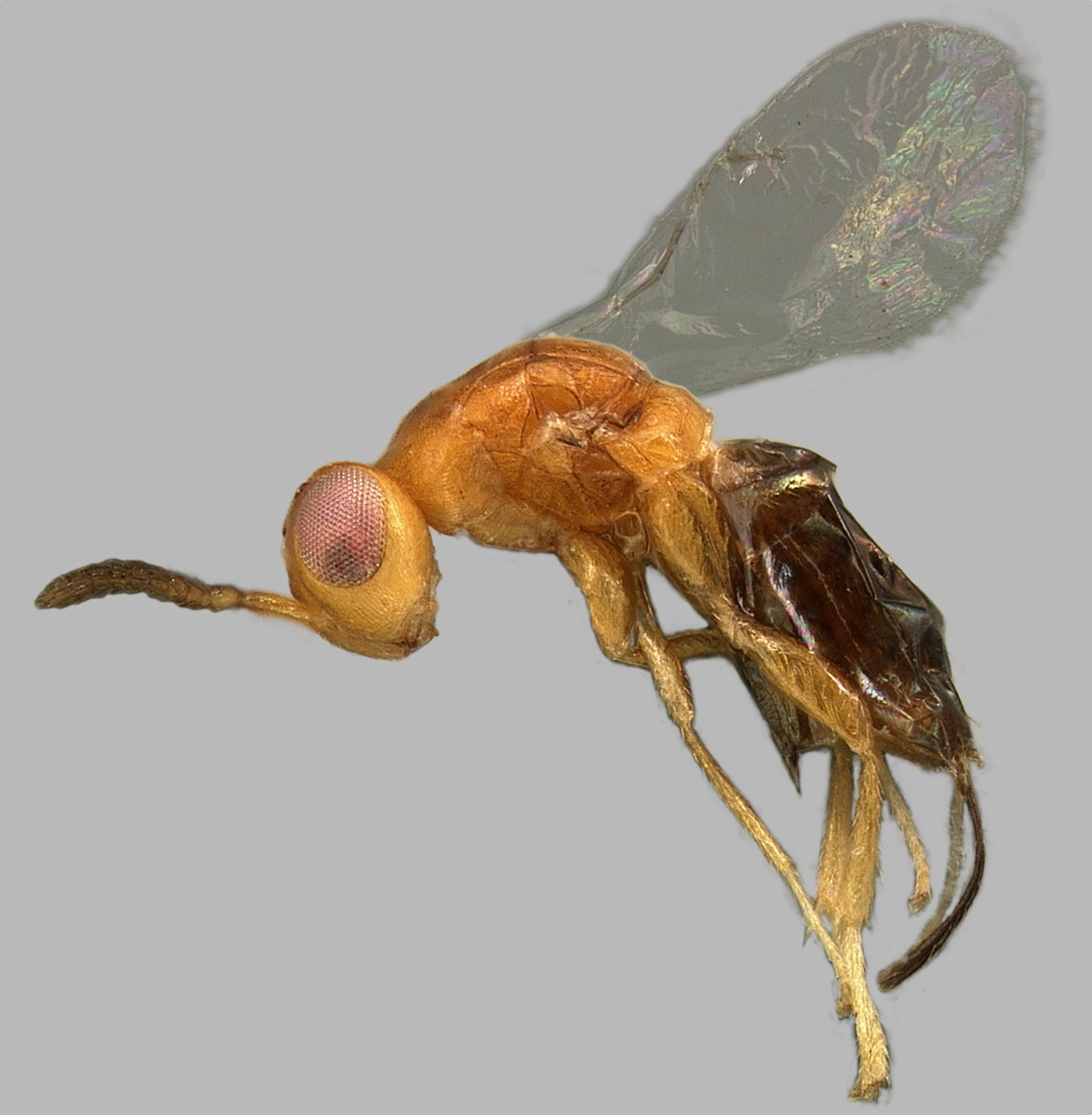

Supplement: Supplemental Information 1 — Multi-entry taxonomic key for Idarnes incertus species-group. The kay is assembled in Lucid: http://www.lucidcentral.com [file peerj-05-2842-s001.zip › Idarnes incertus species group/Media/Images/hab_amacayacuensis.jpg]

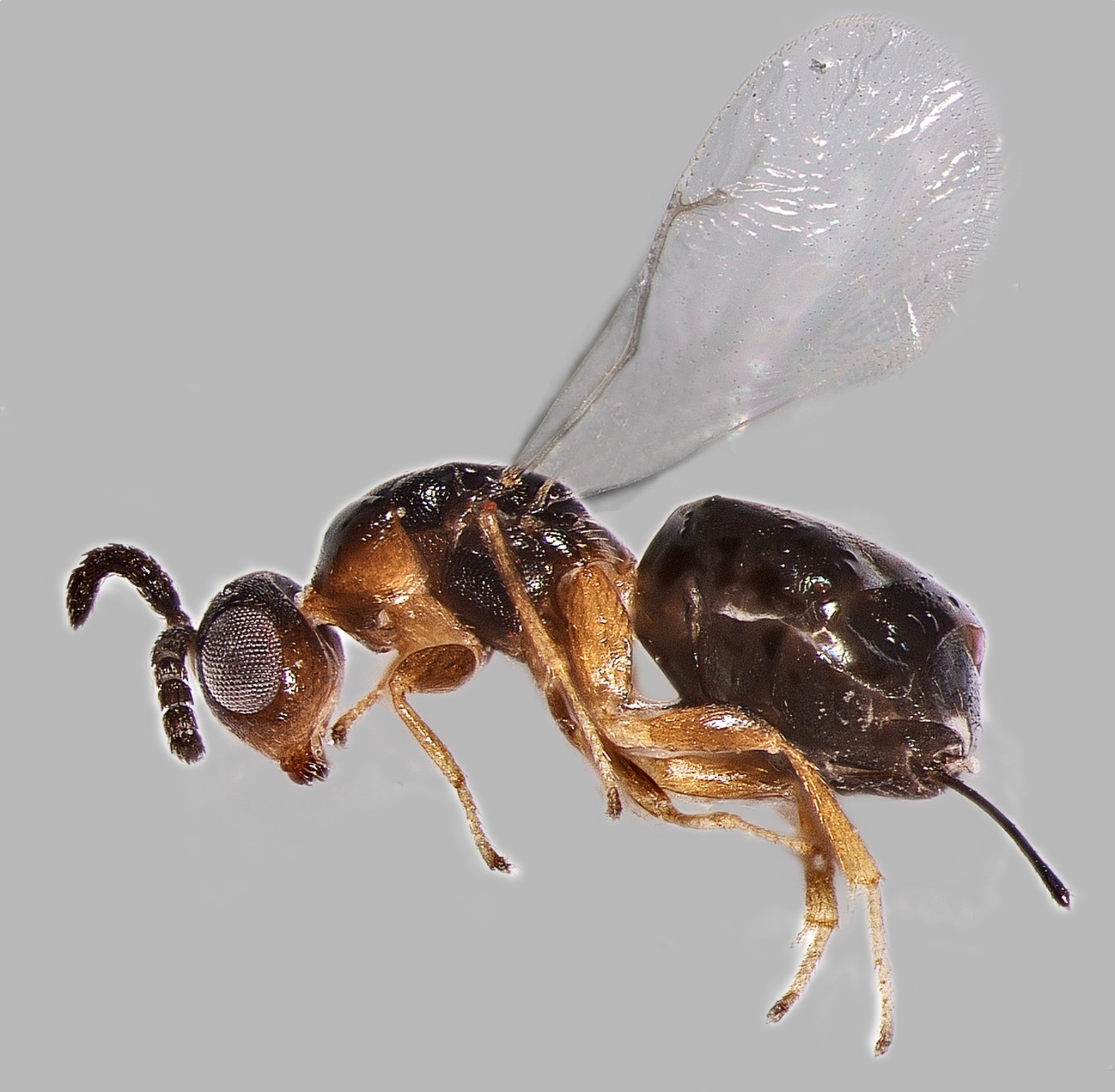

Supplement: Supplemental Information 1 — Multi-entry taxonomic key for Idarnes incertus species-group. The kay is assembled in Lucid: http://www.lucidcentral.com [file peerj-05-2842-s001.zip › Idarnes incertus species group/Media/Images/hab_amazonicus.jpg]

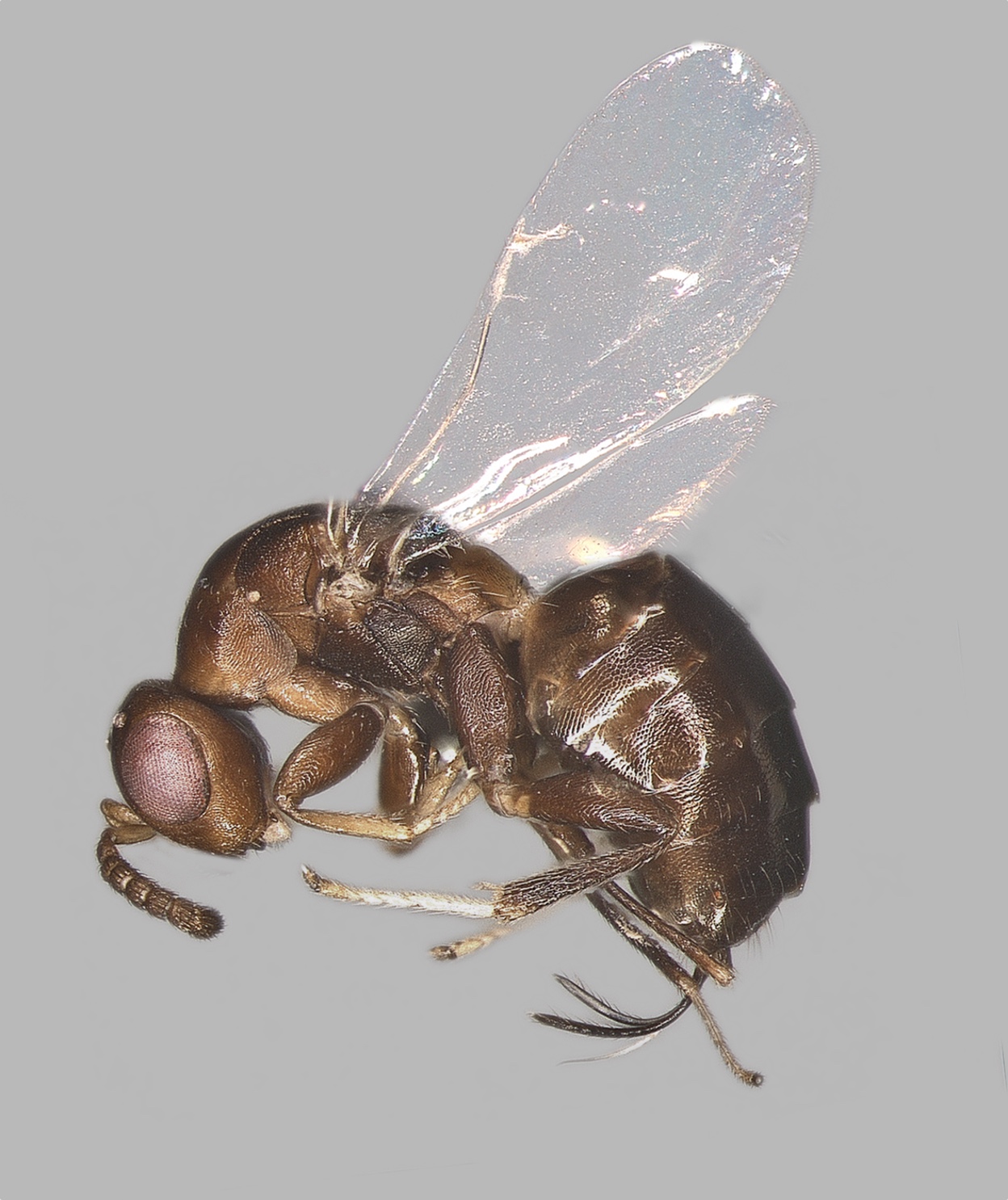

Supplement: Supplemental Information 1 — Multi-entry taxonomic key for Idarnes incertus species-group. The kay is assembled in Lucid: http://www.lucidcentral.com [file peerj-05-2842-s001.zip › Idarnes incertus species group/Media/Images/hab_americanae.jpg]

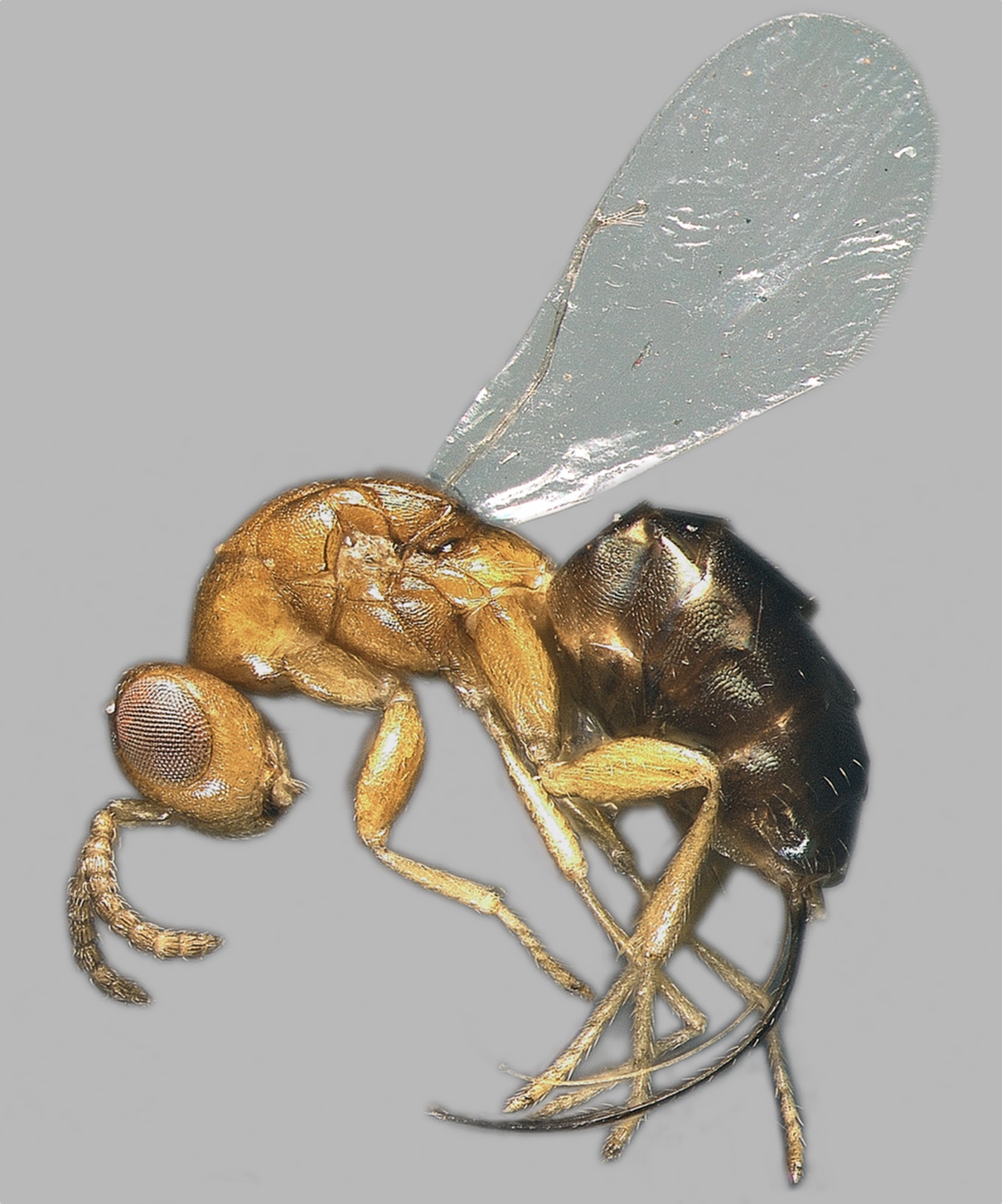

Supplement: Supplemental Information 1 — Multi-entry taxonomic key for Idarnes incertus species-group. The kay is assembled in Lucid: http://www.lucidcentral.com [file peerj-05-2842-s001.zip › Idarnes incertus species group/Media/Images/hab_aureonigrus.jpg]

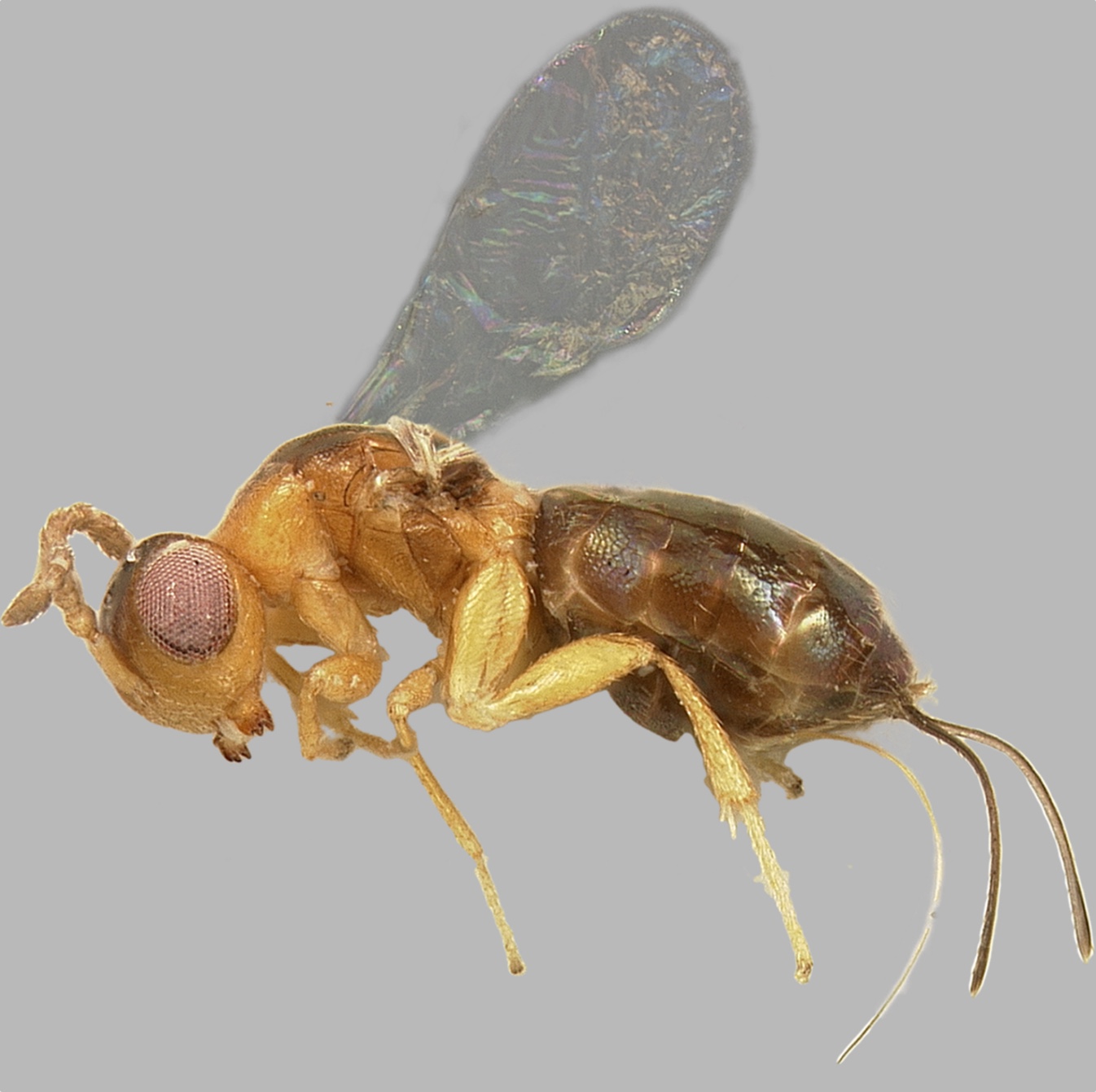

Supplement: Supplemental Information 1 — Multi-entry taxonomic key for Idarnes incertus species-group. The kay is assembled in Lucid: http://www.lucidcentral.com [file peerj-05-2842-s001.zip › Idarnes incertus species group/Media/Images/hab_badiovertex.jpg]

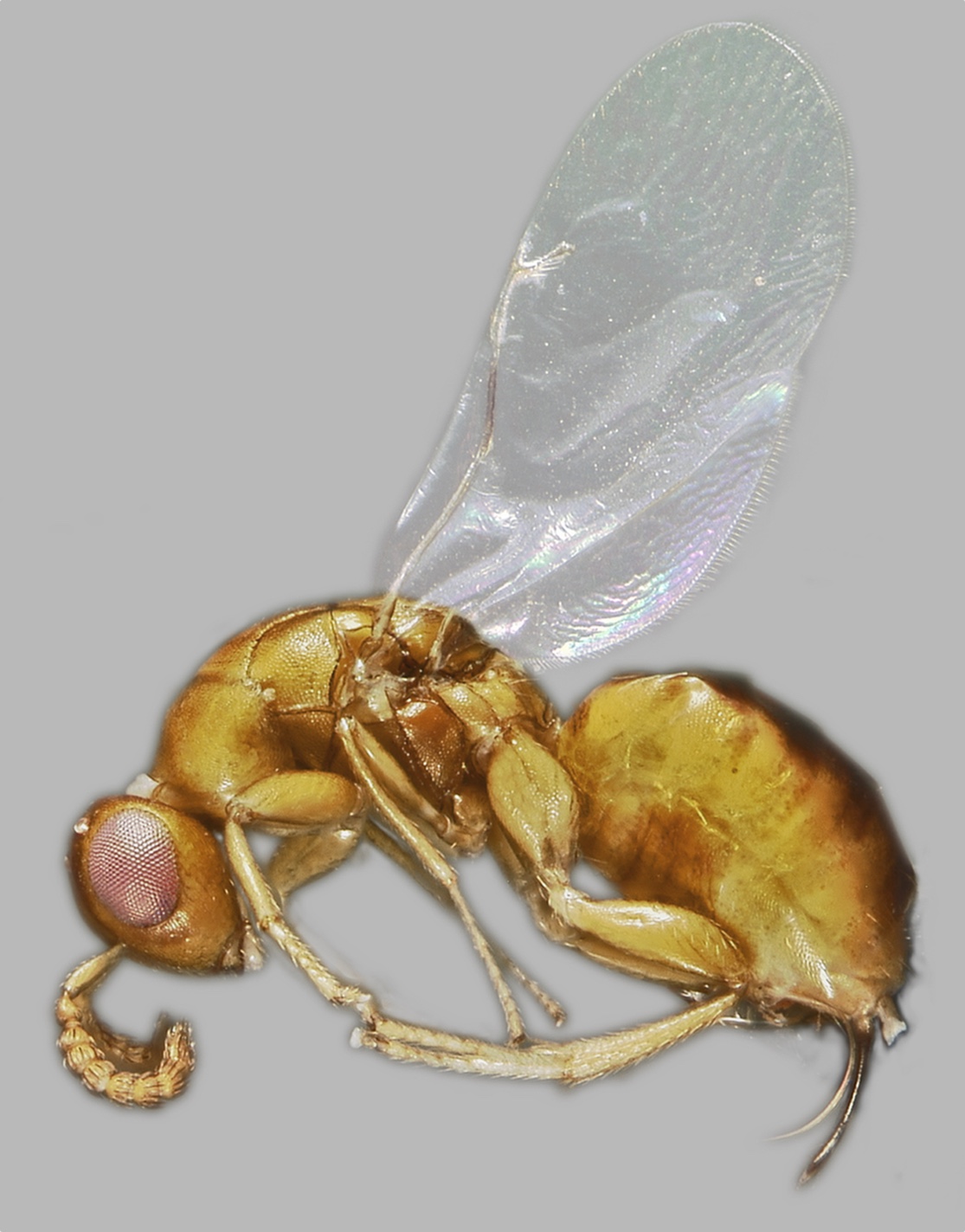

Supplement: Supplemental Information 1 — Multi-entry taxonomic key for Idarnes incertus species-group. The kay is assembled in Lucid: http://www.lucidcentral.com [file peerj-05-2842-s001.zip › Idarnes incertus species group/Media/Images/hab_brevis.jpg]

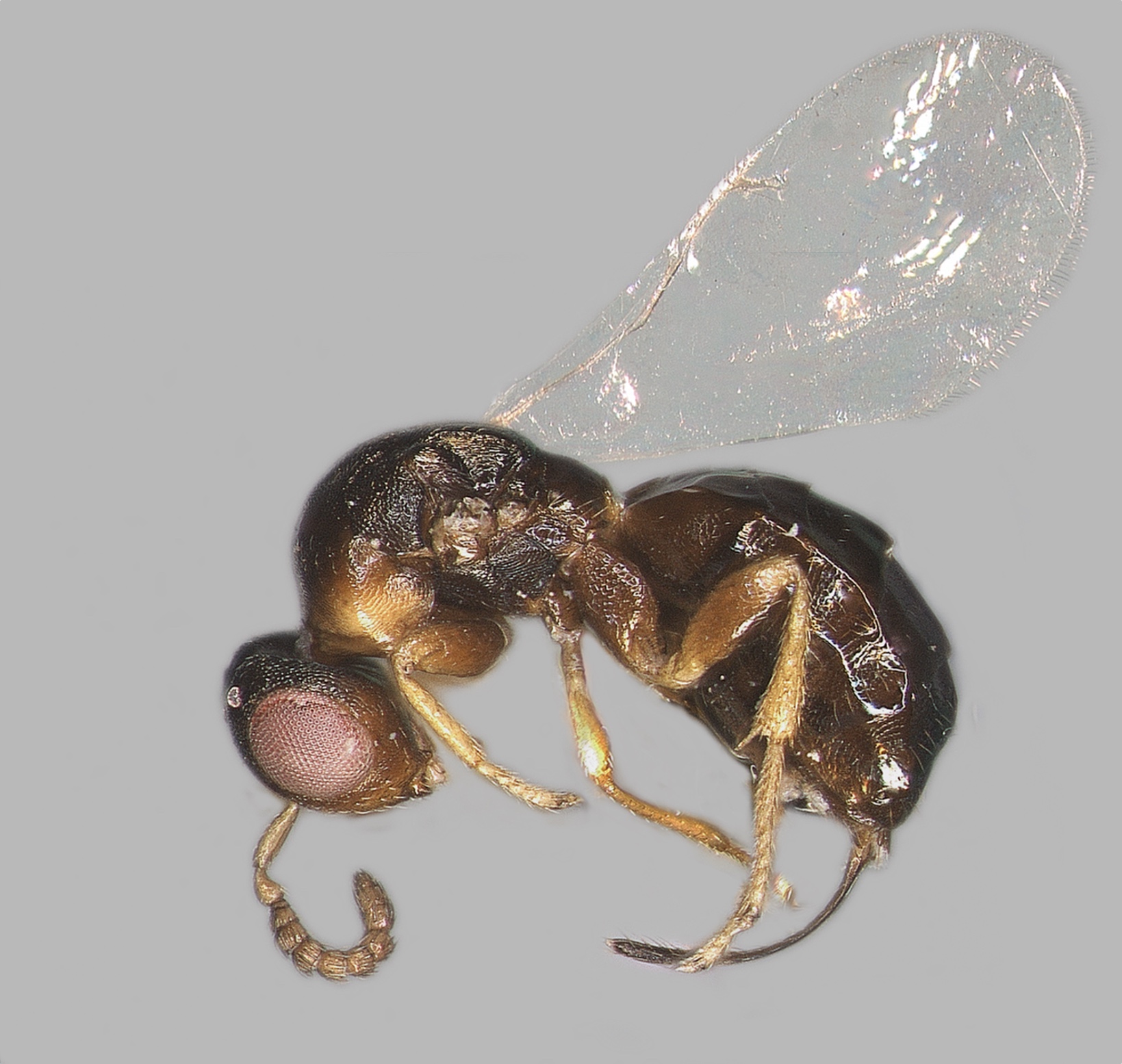

Supplement: Supplemental Information 1 — Multi-entry taxonomic key for Idarnes incertus species-group. The kay is assembled in Lucid: http://www.lucidcentral.com [file peerj-05-2842-s001.zip › Idarnes incertus species group/Media/Images/hab_brunneus.jpg]

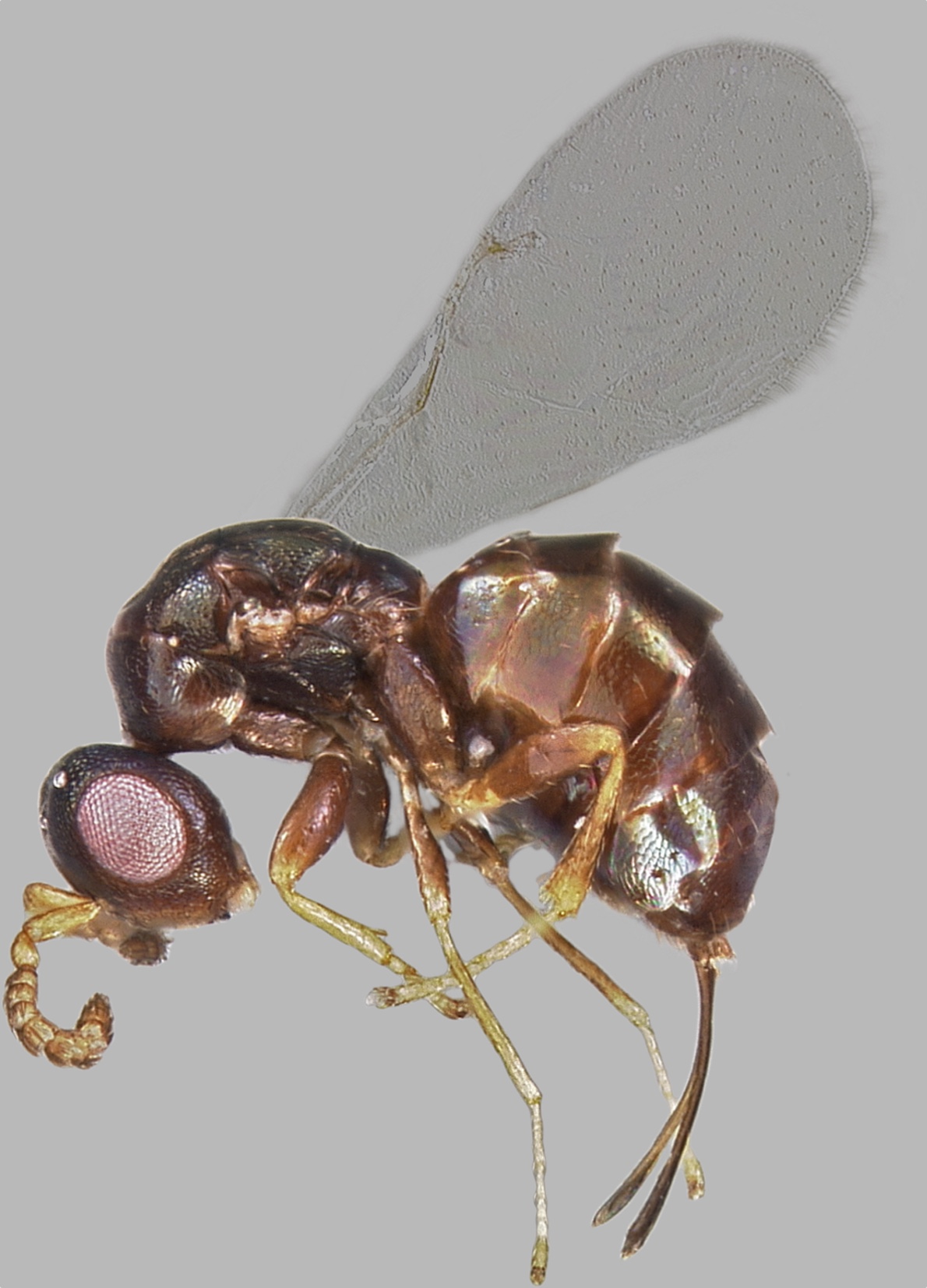

Supplement: Supplemental Information 1 — Multi-entry taxonomic key for Idarnes incertus species-group. The kay is assembled in Lucid: http://www.lucidcentral.com [file peerj-05-2842-s001.zip › Idarnes incertus species group/Media/Images/hab_comptoni.jpg]

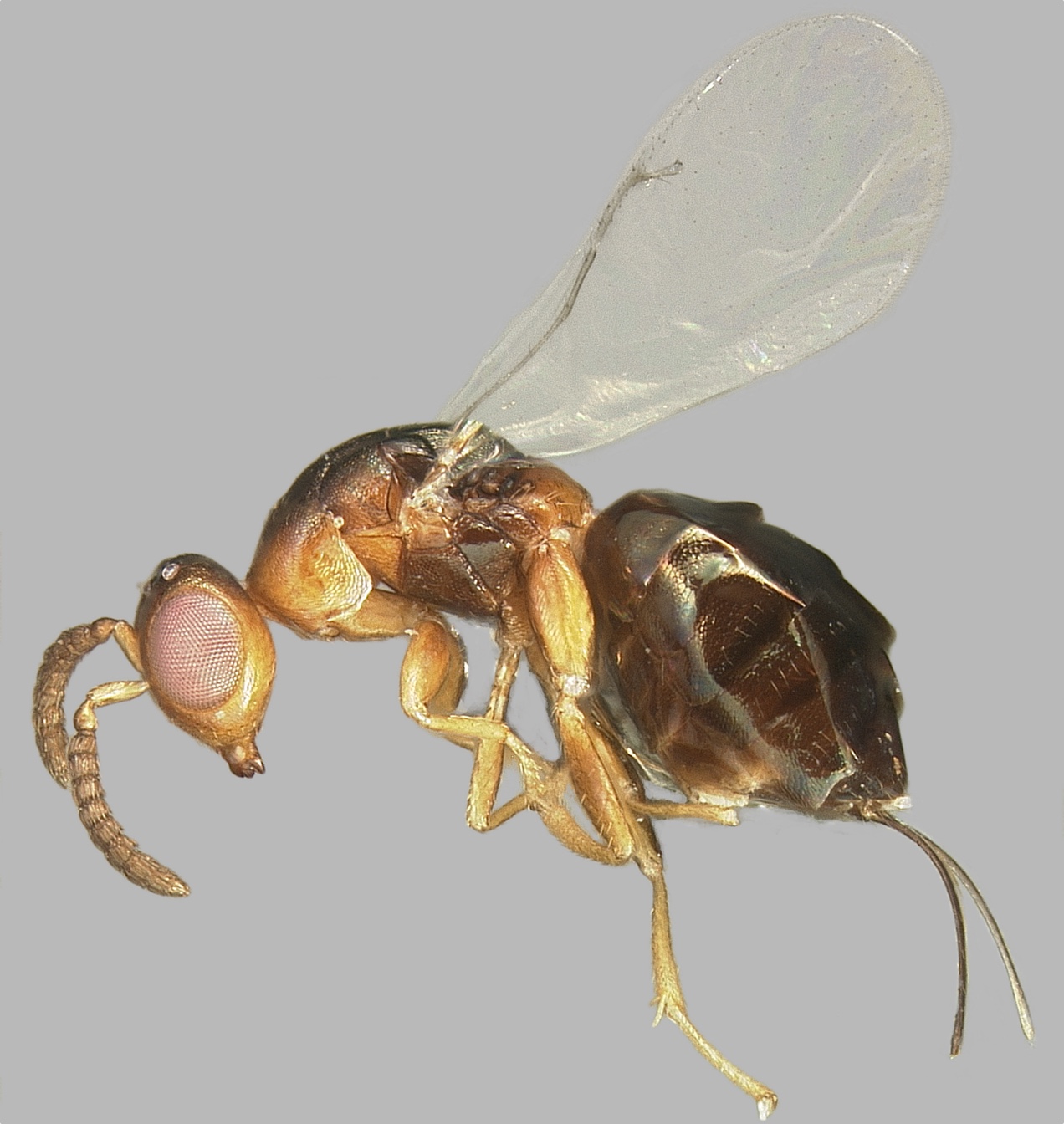

Supplement: Supplemental Information 1 — Multi-entry taxonomic key for Idarnes incertus species-group. The kay is assembled in Lucid: http://www.lucidcentral.com [file peerj-05-2842-s001.zip › Idarnes incertus species group/Media/Images/hab_cremersae.jpg]

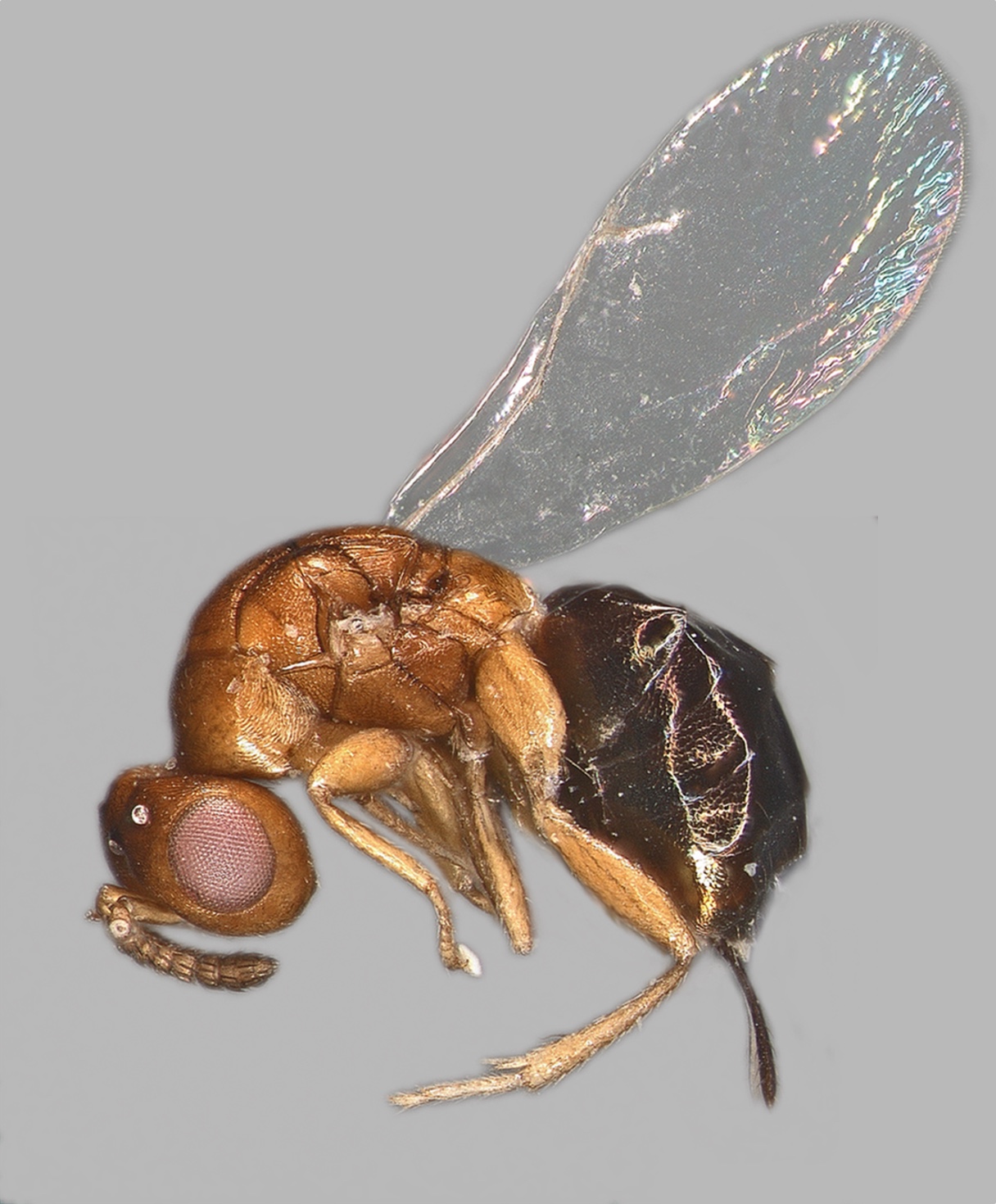

Supplement: Supplemental Information 1 — Multi-entry taxonomic key for Idarnes incertus species-group. The kay is assembled in Lucid: http://www.lucidcentral.com [file peerj-05-2842-s001.zip › Idarnes incertus species group/Media/Images/hab_dimorphicus.jpg]

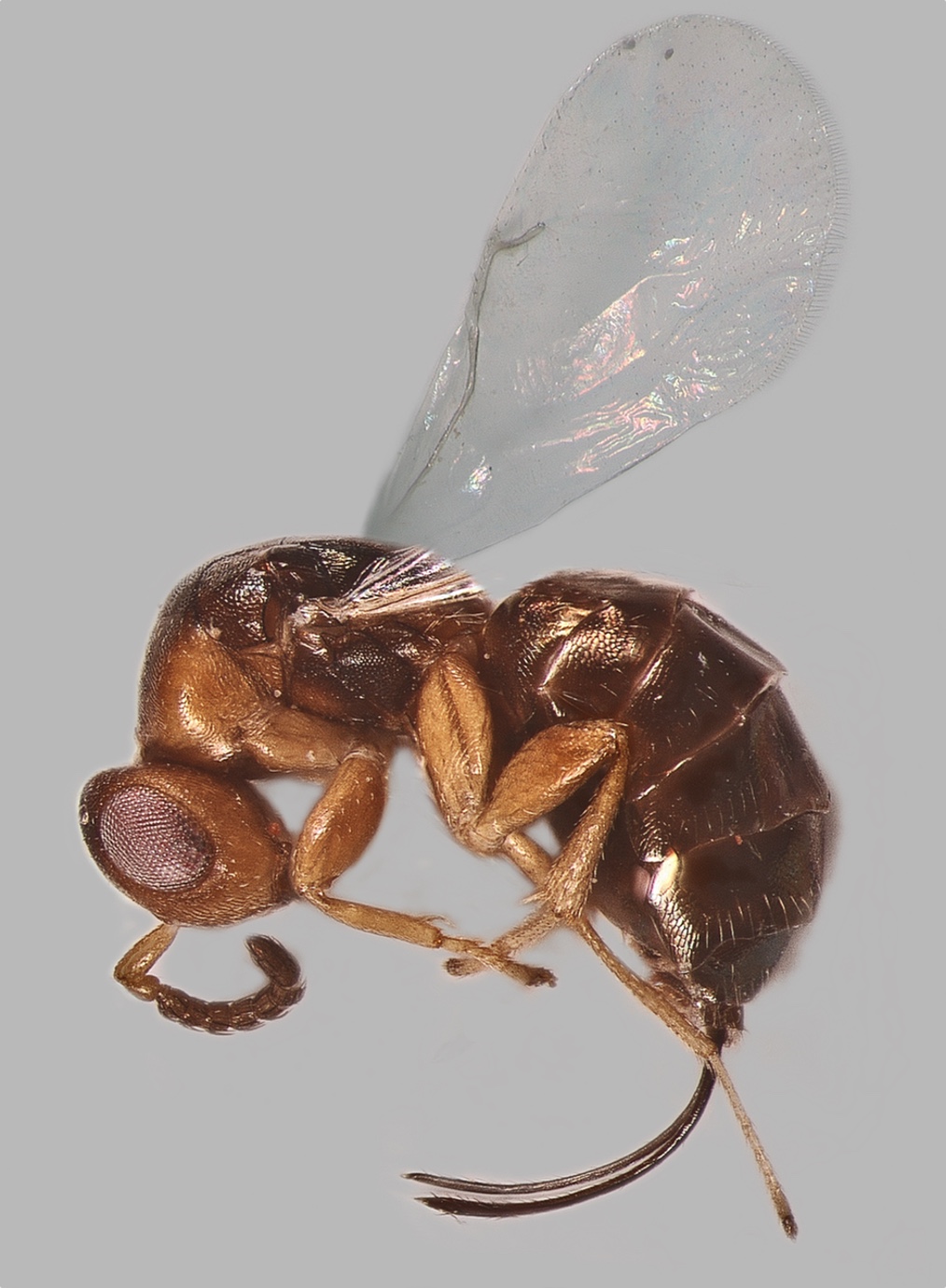

Supplement: Supplemental Information 1 — Multi-entry taxonomic key for Idarnes incertus species-group. The kay is assembled in Lucid: http://www.lucidcentral.com [file peerj-05-2842-s001.zip › Idarnes incertus species group/Media/Images/hab_flavicrus.jpg]

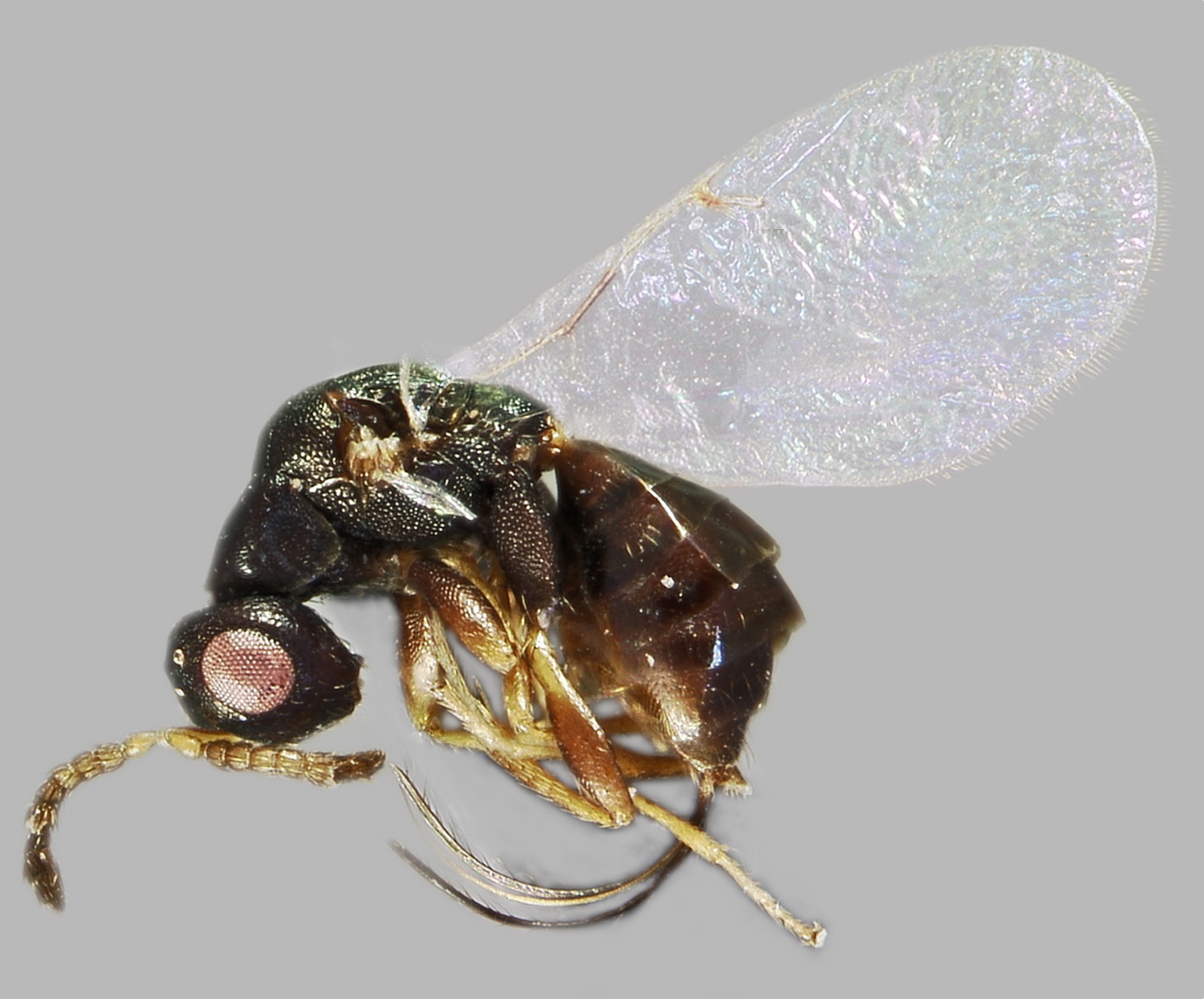

Supplement: Supplemental Information 1 — Multi-entry taxonomic key for Idarnes incertus species-group. The kay is assembled in Lucid: http://www.lucidcentral.com [file peerj-05-2842-s001.zip › Idarnes incertus species group/Media/Images/hab_gibberosus.jpg]

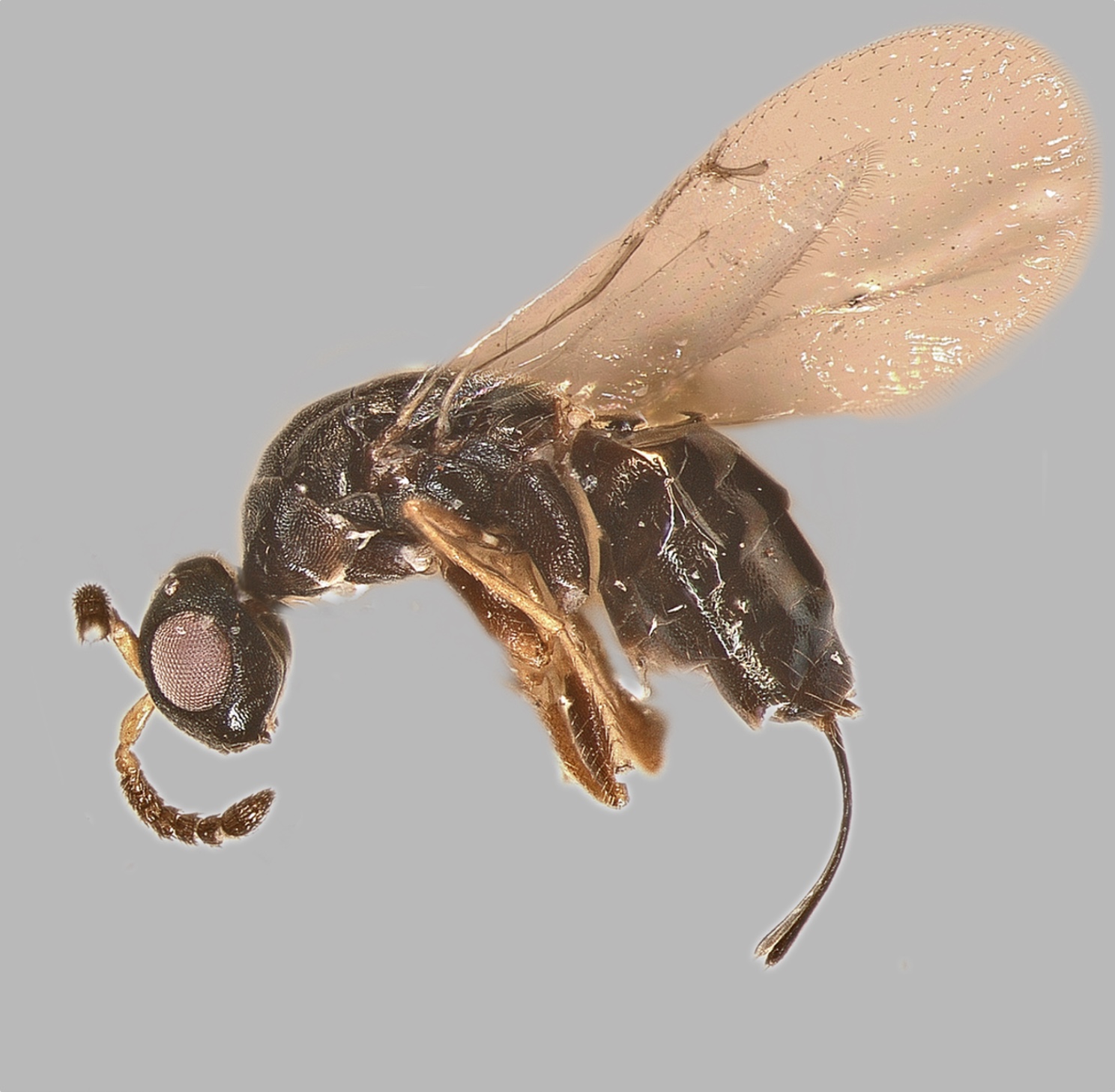

Supplement: Supplemental Information 1 — Multi-entry taxonomic key for Idarnes incertus species-group. The kay is assembled in Lucid: http://www.lucidcentral.com [file peerj-05-2842-s001.zip › Idarnes incertus species group/Media/Images/hab_hansoni.jpg]

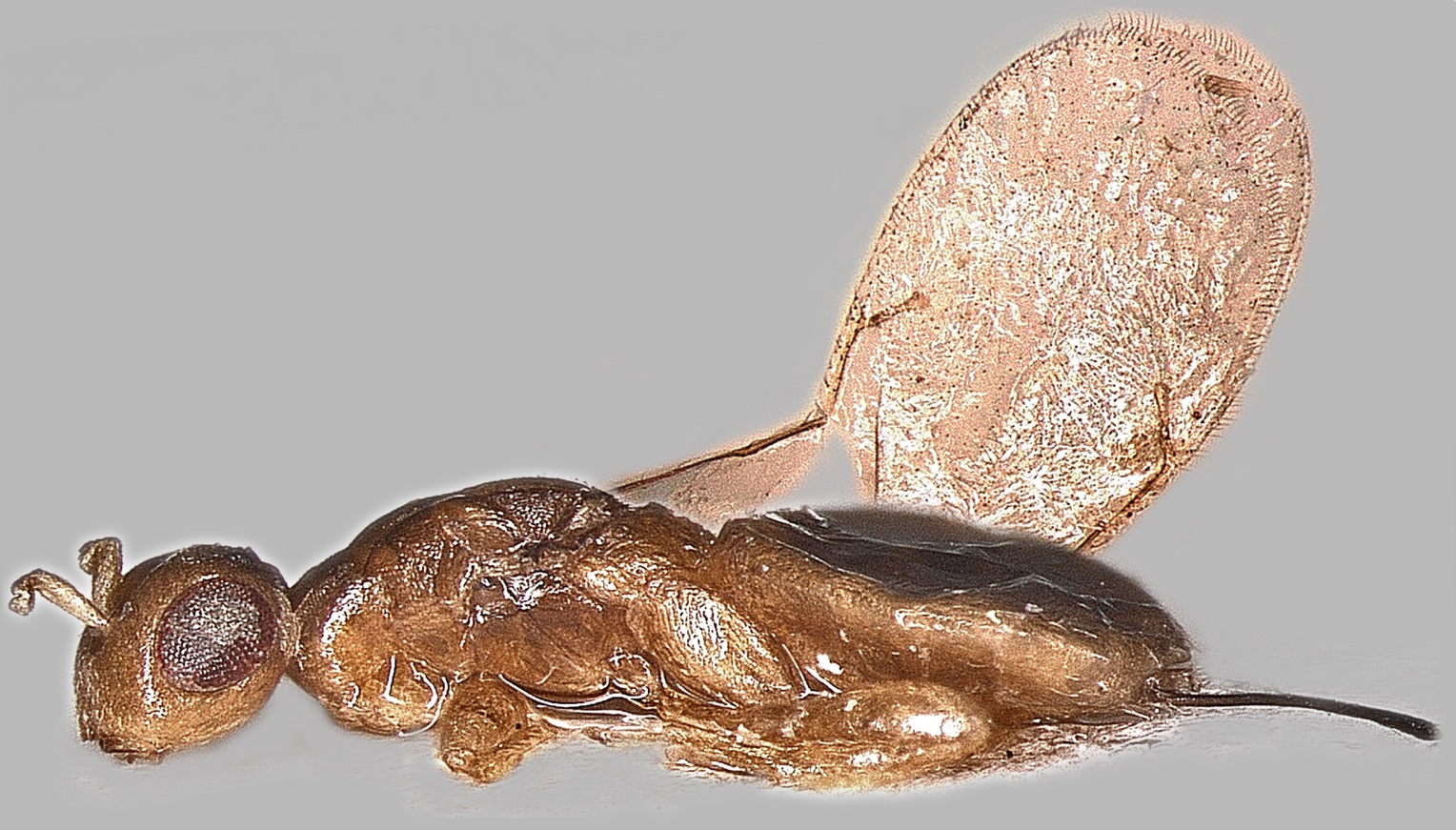

Supplement: Supplemental Information 1 — Multi-entry taxonomic key for Idarnes incertus species-group. The kay is assembled in Lucid: http://www.lucidcentral.com [file peerj-05-2842-s001.zip › Idarnes incertus species group/Media/Images/hab_incertus.jpg]

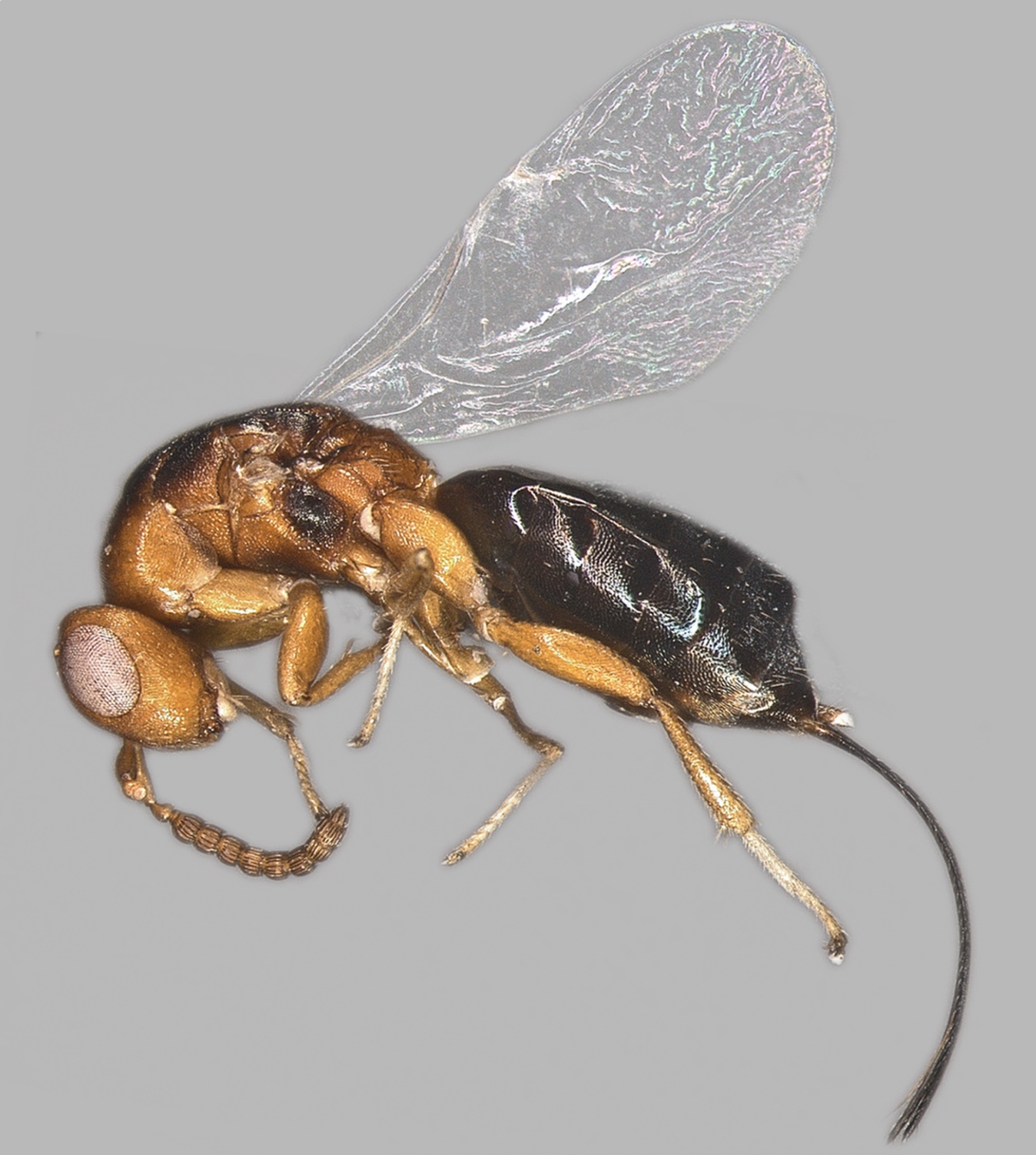

Supplement: Supplemental Information 1 — Multi-entry taxonomic key for Idarnes incertus species-group. The kay is assembled in Lucid: http://www.lucidcentral.com [file peerj-05-2842-s001.zip › Idarnes incertus species group/Media/Images/hab_maximus.jpg]

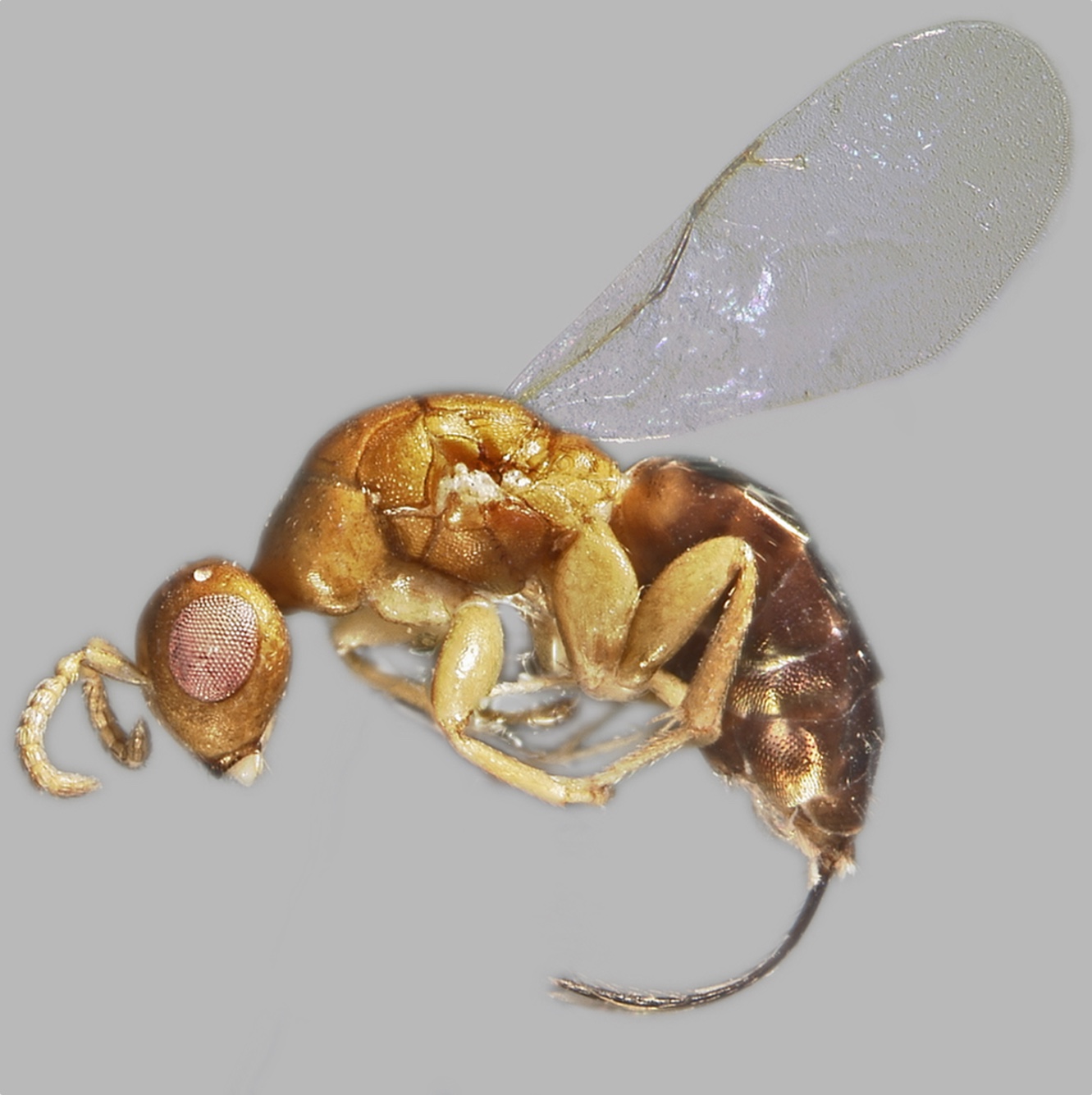

Supplement: Supplemental Information 1 — Multi-entry taxonomic key for Idarnes incertus species-group. The kay is assembled in Lucid: http://www.lucidcentral.com [file peerj-05-2842-s001.zip › Idarnes incertus species group/Media/Images/hab_nigriventris.jpg]

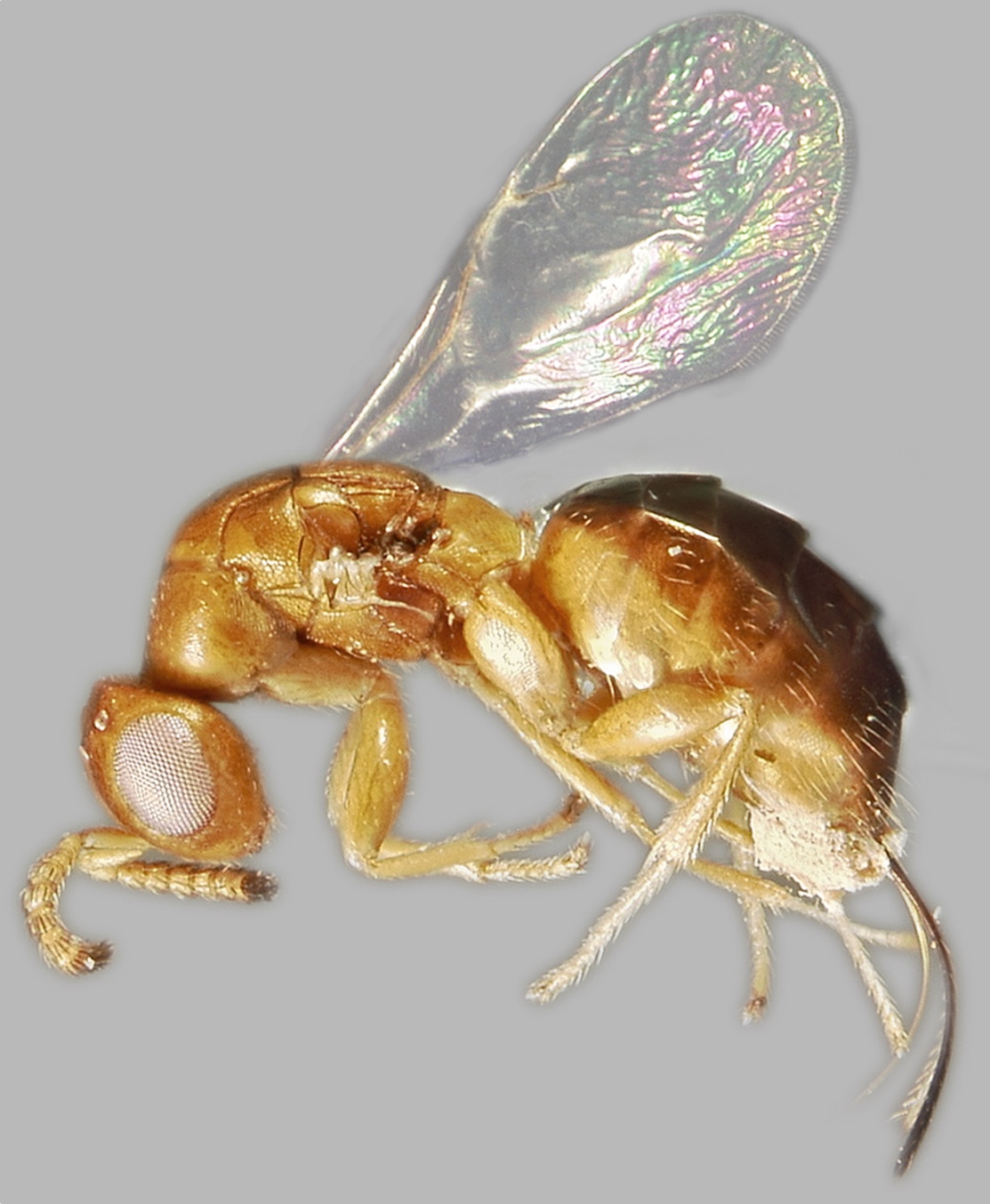

Supplement: Supplemental Information 1 — Multi-entry taxonomic key for Idarnes incertus species-group. The kay is assembled in Lucid: http://www.lucidcentral.com [file peerj-05-2842-s001.zip › Idarnes incertus species group/Media/Images/hab_pseudoflavus.jpg]

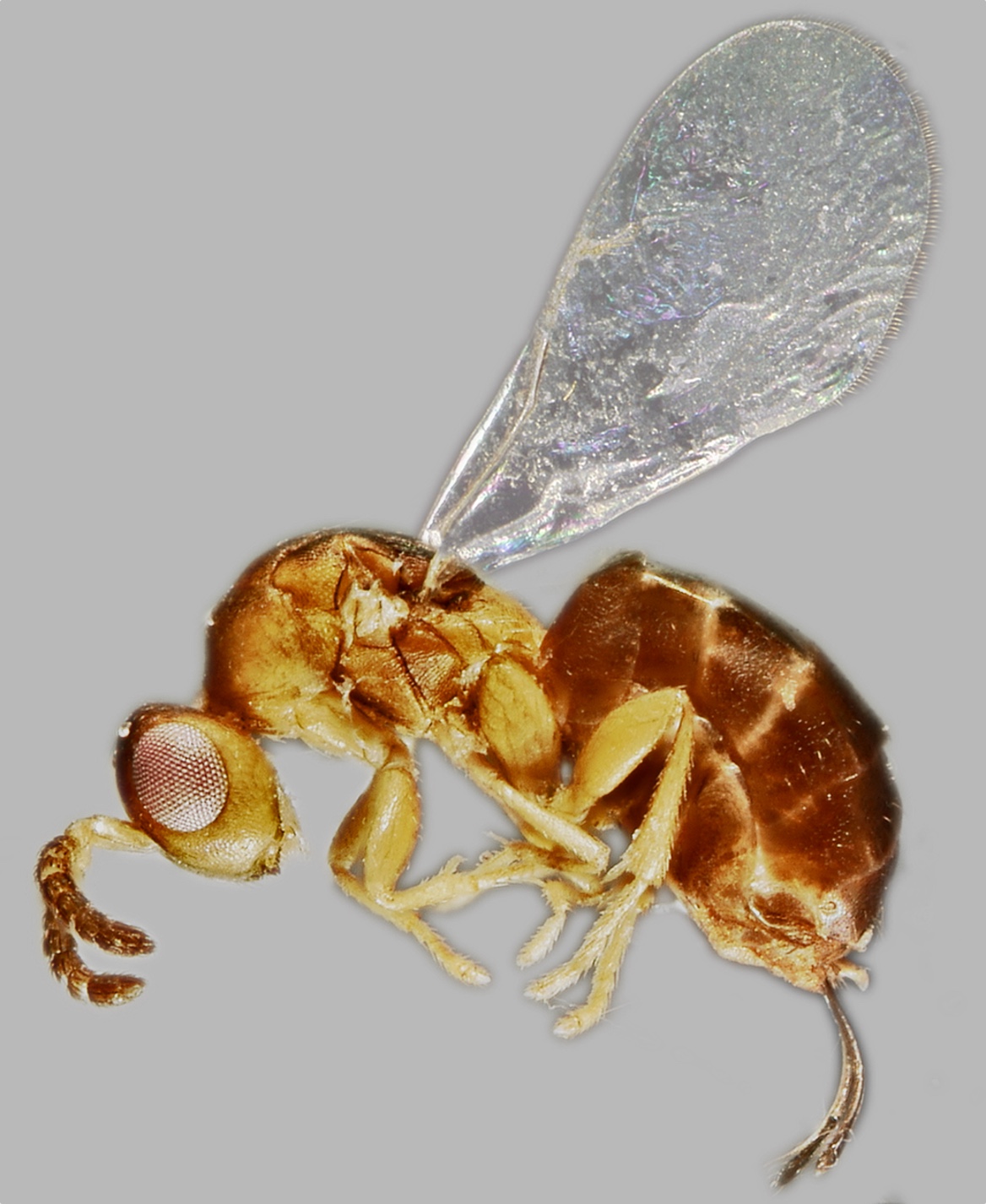

Supplement: Supplemental Information 1 — Multi-entry taxonomic key for Idarnes incertus species-group. The kay is assembled in Lucid: http://www.lucidcentral.com [file peerj-05-2842-s001.zip › Idarnes incertus species group/Media/Images/hab_williamsi.jpg]

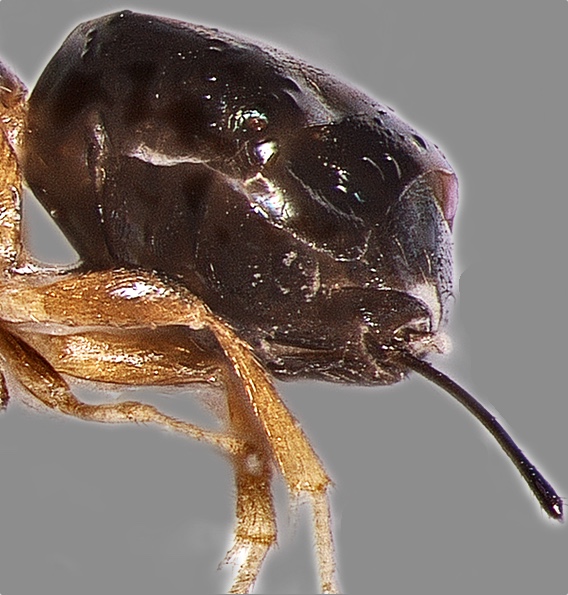

Supplement: Supplemental Information 1 — Multi-entry taxonomic key for Idarnes incertus species-group. The kay is assembled in Lucid: http://www.lucidcentral.com [file peerj-05-2842-s001.zip › Idarnes incertus species group/Media/Images/mesosoma dark.jpg]

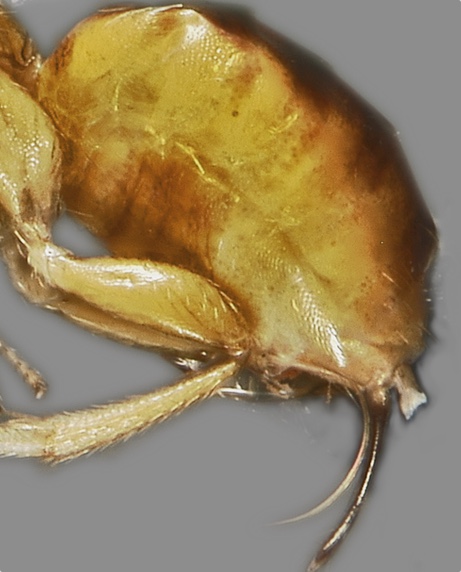

Supplement: Supplemental Information 1 — Multi-entry taxonomic key for Idarnes incertus species-group. The kay is assembled in Lucid: http://www.lucidcentral.com [file peerj-05-2842-s001.zip › Idarnes incertus species group/Media/Images/mesosoma yellow.jpg]

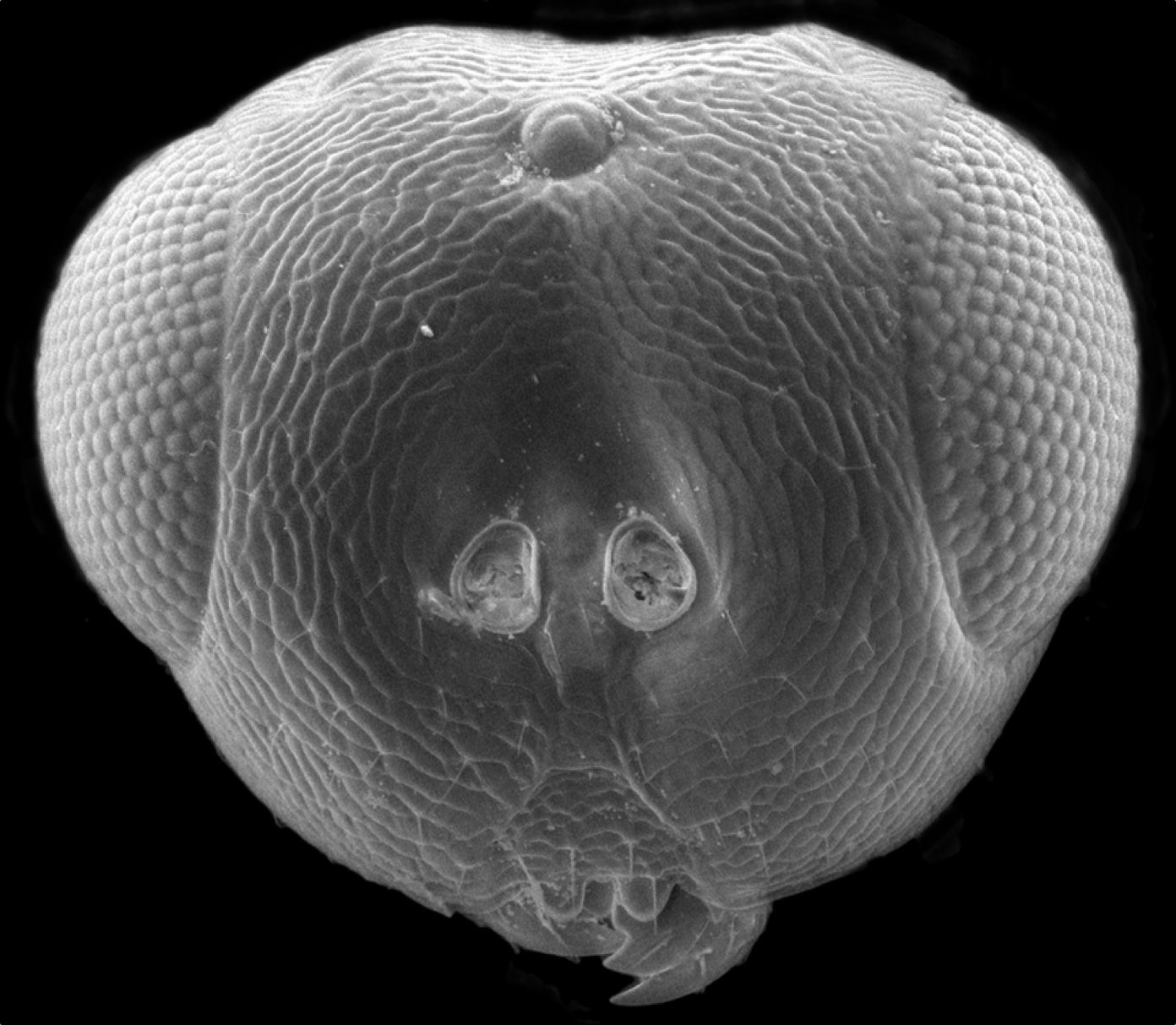

Supplement: Supplemental Information 1 — Multi-entry taxonomic key for Idarnes incertus species-group. The kay is assembled in Lucid: http://www.lucidcentral.com [file peerj-05-2842-s001.zip › Idarnes incertus species group/Media/Images/torulus middle.jpg]

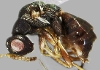

Supplement: Supplemental Information 1 — Multi-entry taxonomic key for Idarnes incertus species-group. The kay is assembled in Lucid: http://www.lucidcentral.com [file peerj-05-2842-s001.zip › Idarnes incertus species group/Media/Thumbs/001_A Body black_1_TN.jpg]

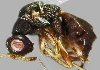

Supplement: Supplemental Information 1 — Multi-entry taxonomic key for Idarnes incertus species-group. The kay is assembled in Lucid: http://www.lucidcentral.com [file peerj-05-2842-s001.zip › Idarnes incertus species group/Media/Thumbs/001_A Body black_TN.jpg]

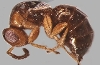

Supplement: Supplemental Information 1 — Multi-entry taxonomic key for Idarnes incertus species-group. The kay is assembled in Lucid: http://www.lucidcentral.com [file peerj-05-2842-s001.zip › Idarnes incertus species group/Media/Thumbs/001_B_Body brown yellow_TN.jpg]

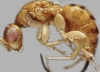

Supplement: Supplemental Information 1 — Multi-entry taxonomic key for Idarnes incertus species-group. The kay is assembled in Lucid: http://www.lucidcentral.com [file peerj-05-2842-s001.zip › Idarnes incertus species group/Media/Thumbs/001_C Body yellow_TN.jpg]

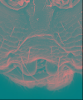

Supplement: Supplemental Information 1 — Multi-entry taxonomic key for Idarnes incertus species-group. The kay is assembled in Lucid: http://www.lucidcentral.com [file peerj-05-2842-s001.zip › Idarnes incertus species group/Media/Thumbs/002_A Epistomal delim clypeus_TN.jpg]

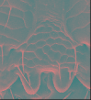

Supplement: Supplemental Information 1 — Multi-entry taxonomic key for Idarnes incertus species-group. The kay is assembled in Lucid: http://www.lucidcentral.com [file peerj-05-2842-s001.zip › Idarnes incertus species group/Media/Thumbs/002_B Epistomal Shallow_TN.jpg]

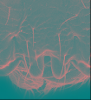

Supplement: Supplemental Information 1 — Multi-entry taxonomic key for Idarnes incertus species-group. The kay is assembled in Lucid: http://www.lucidcentral.com [file peerj-05-2842-s001.zip › Idarnes incertus species group/Media/Thumbs/002_C Epistomal inconspicuous_TN.jpg]

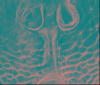

Supplement: Supplemental Information 1 — Multi-entry taxonomic key for Idarnes incertus species-group. The kay is assembled in Lucid: http://www.lucidcentral.com [file peerj-05-2842-s001.zip › Idarnes incertus species group/Media/Thumbs/003_A Supraclyp narrow_TN.jpg]

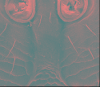

Supplement: Supplemental Information 1 — Multi-entry taxonomic key for Idarnes incertus species-group. The kay is assembled in Lucid: http://www.lucidcentral.com [file peerj-05-2842-s001.zip › Idarnes incertus species group/Media/Thumbs/003_B Supraclyp as wide_TN.jpg]

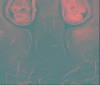

Supplement: Supplemental Information 1 — Multi-entry taxonomic key for Idarnes incertus species-group. The kay is assembled in Lucid: http://www.lucidcentral.com [file peerj-05-2842-s001.zip › Idarnes incertus species group/Media/Thumbs/003_C Supraclyp wider_TN.jpg]

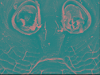

Supplement: Supplemental Information 1 — Multi-entry taxonomic key for Idarnes incertus species-group. The kay is assembled in Lucid: http://www.lucidcentral.com [file peerj-05-2842-s001.zip › Idarnes incertus species group/Media/Thumbs/004_A Subantennal short_TN.jpg]

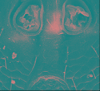

Supplement: Supplemental Information 1 — Multi-entry taxonomic key for Idarnes incertus species-group. The kay is assembled in Lucid: http://www.lucidcentral.com [file peerj-05-2842-s001.zip › Idarnes incertus species group/Media/Thumbs/004_B Subantennal as long_TN.jpg]

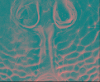

Supplement: Supplemental Information 1 — Multi-entry taxonomic key for Idarnes incertus species-group. The kay is assembled in Lucid: http://www.lucidcentral.com [file peerj-05-2842-s001.zip › Idarnes incertus species group/Media/Thumbs/004_C Subantennal longer_TN.jpg]

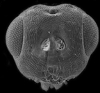

Supplement: Supplemental Information 1 — Multi-entry taxonomic key for Idarnes incertus species-group. The kay is assembled in Lucid: http://www.lucidcentral.com [file peerj-05-2842-s001.zip › Idarnes incertus species group/Media/Thumbs/005_A Torulus closer ocellus_TN.jpg]

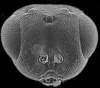

Supplement: Supplemental Information 1 — Multi-entry taxonomic key for Idarnes incertus species-group. The kay is assembled in Lucid: http://www.lucidcentral.com [file peerj-05-2842-s001.zip › Idarnes incertus species group/Media/Thumbs/005_B Torulus closer oral margin_TN.jpg]

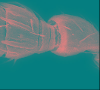

Supplement: Supplemental Information 1 — Multi-entry taxonomic key for Idarnes incertus species-group. The kay is assembled in Lucid: http://www.lucidcentral.com [file peerj-05-2842-s001.zip › Idarnes incertus species group/Media/Thumbs/006_A funicle 2 anelli_TN.jpg]

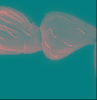

Supplement: Supplemental Information 1 — Multi-entry taxonomic key for Idarnes incertus species-group. The kay is assembled in Lucid: http://www.lucidcentral.com [file peerj-05-2842-s001.zip › Idarnes incertus species group/Media/Thumbs/006_B funicle 2 anelli (nearly fused)_TN.jpg]

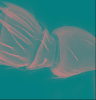

Supplement: Supplemental Information 1 — Multi-entry taxonomic key for Idarnes incertus species-group. The kay is assembled in Lucid: http://www.lucidcentral.com [file peerj-05-2842-s001.zip › Idarnes incertus species group/Media/Thumbs/006_C funicle 1 anellus_TN.jpg]

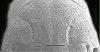

Supplement: Supplemental Information 1 — Multi-entry taxonomic key for Idarnes incertus species-group. The kay is assembled in Lucid: http://www.lucidcentral.com [file peerj-05-2842-s001.zip › Idarnes incertus species group/Media/Thumbs/007_A Mesoscutum with median striae_TN.jpg]
