# Supplementary material for: Taxonomic revision and molecular phylogenetics of the Idarnes incertus species-group (Hymenoptera, Agaonidae, Sycophaginae)
Source: PeerJ. 2017 Jan 5;5:e2842. doi: 10.7717/peerj.2842 (PMC5289451; doi:10.7717/peerj.2842)
Supplement: Supplemental Information 5 — Idarnes incertus sp. gp. Species re-descriptions and additional images. [file peerj-05-2842-s005.doc]

Farache, F. H. A.; Cruaud, A; Genson, G.; Rasplus, J.Y.R. & Pereira, R.A.S. (2016). **Taxonomic revision and molecular phylogenetics of the *Idarnes incertus* species group (Hymenoptera, Agaonidae, Sycophaginae)**. *PeerJ*.

**Supplementary material 2.** *Idarnes incertus* sp. gp. Species re-descriptions and additional images.

*Abbreviations for measurements* used in the text include: POL, distance between posterior ocelli; OOL, distance between posterior ocellus and eye margin

***Idarnes brasiliensis* (Mayr, 1906) (comb. nov.)**

Fig. S1

1906 Mayr, G. *Entomologische Zeitung Wien* 25:185. Description (♀♂) (Comb. *Sycophila brasiliensis*)

**Type material.** Lectotype (here designated) **BRAZIL:** **Santa Catarina:** Blumenau, 1♂, [no date], Fritz Müller, ex *Ficus doliaria* (=*F. gomelleira*) (NMW).

**Diagnosis** (♂)**.** Body colour predominantly yellow orange. Mesosoma 1.4× as long as wide. Axillula longitudinally striated. Frenal sulcus inconspicuous. Metascutellum inconspicuous. Propodeal median line present, conspicuous. Postmarginal vein very short, as long as 1/3× stigmal vein length.

**Female:** Described by Mayr (1906), but we could not find any female specimens at NMW.

**Host plant**. *Ficus gomelleira* Kunth & Bouché.

**Remarks:** There is only one male specimen collected by Mayr at NMW, minuten-mounted and decapitated. Despite the absence of head, the following characters ascertain its position within the *Idarnes incertus* species group: (1) Winged male, (2) body colour, (3) postmarginal vein compared to stigmal, (4) shape of mesoscutellum and (4) striated axillulae.


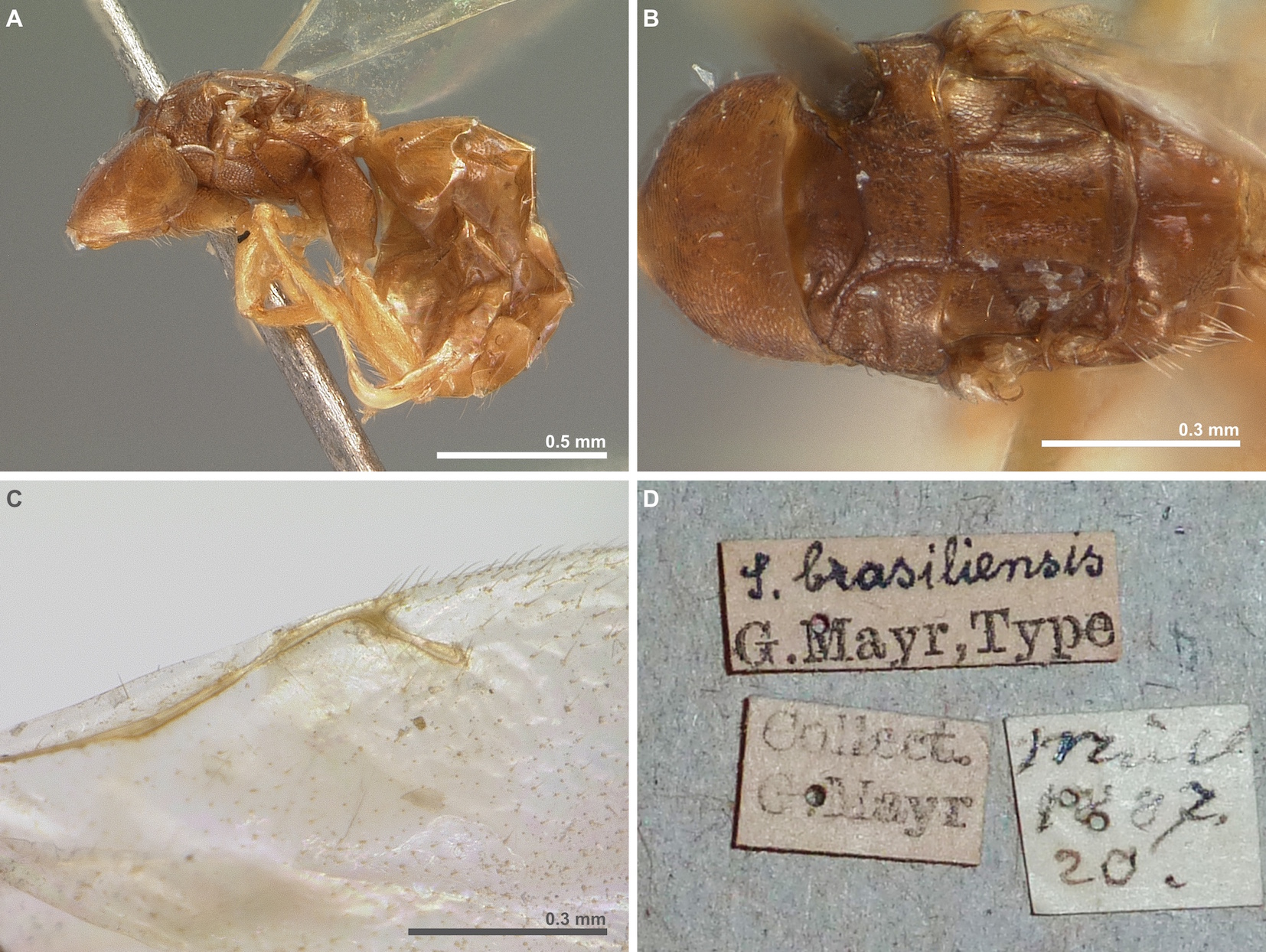


Fig. S1: *Idarnes brasiliensis* (Mayr, 1906). Lectotype. A, *habitus*; B, mesosoma in dorsal view; C, wing venation; D, specimen labels.

***Idarnes hansoni* Bouček, 1993**

(Figs 3E, 5E, 7E, 9E, 11E, 13E, 15E)

1993 Bouček, Z., *Journal of Natural History* 27: 202-203, Fig. 38. Description (♀♂).

**Type material.** Holotype: ♀, **COSTA RICA:** **San José:** Zarcero, Llano Bonito, XII.1987, Hanson P., ex *Ficus* (BMNH, examined).

Paratypes. **COSTA RICA: Guanacaste**: N.P. Santa Rosa, 1♀, I.1987, Gauld, I (BMNH); **San José:** Zarcero, Llano Bonito, 4♀, XII.1987, Hanson P., ex *Ficus* (BMNH), Zurqui de Moravia, 1600m, 1♀, 2♂, 7-9.IX.1991, Hanson P., ex *Ficus brenesi* (= *F. hartwegii*) (EBCR, USNM, BMNH)

**Diagnosis**. Body predominantly brown black. Supraclypeal area wider than torulus. Subantennal groove as long as torulus. Distance from torulus to median ocellus 0.9× distance from torulus to oral margin. Metascutellum inconspicuous. Anterior margin of propodeum not angulose medially. Ovipositor sheaths 1.4–1.5× as long as hind tibia.

**Female***.*

*Size and colour.* Body length 1.8 mm. Ovipositor length 0.6 mm. Predominantly brown black. Scape and pedicel yellow. Flagellum brown. Legs brown black. Tibia, tarsi, proximal portion of femur, trochanter and trochantellus yellow orange.

*Head.* Supraclypeal area wider than torulus. Subantennal groove as long as torulus. Distance from torulus to median ocellus 0.9× distance from torulus to oral margin. POL 3× OOL. Scape 2× as long as pedicel. Antenna with one anellus. First funicular segment 0.7× as long as wide, with 4–5 multiporous plate sensillae.

*Mesosoma.* Mesoscutum reticulate to punctate reticulate. Mesoscutum and scutellar-axillar complex not strongly curved in lateral view. Notaulus with shallow crenulation. Mesoscutellum 1.3× as long as wide near transscutal articulation. Axillula longitudinally striate to reticulate. Frenal sulcus barely crenulate and inconspicuous. Metascutellum inconspicuous. Anterior margin of propodeum not angulose medially. Propodeal median line present as a faint longitudinal reticulation. Stigmal vein 0.9–1× as long as marginal vein, with two adstigmal setae. Postmarginal vein nearly as long as 0.5× stigmal vein length.

*Metasoma.* Ovipositor sheaths 1.4–1.5× as long as hind tibia.

**Male**. Not observed.

**Host plant**. *Ficus hartwegii* (Miquel) Miquel

**Remarks.** One paratype analysed (Guanacaste, N. P. Santa Rosa, January 1987, I. Gauld leg. (BMNH)) actually belongs to an undescribed species. Since only one specimen is known and because we have no host information, we decided not to describe it waiting for more information and specimens. This species can be distinguished from *I. hansoni* by the following characters: (1) head, pronotum, and propodeum yellow brown, (2) propodeal median line present and conspicuous, (3) anterior margin of propodeum slightly angulose medially.

***Idarnes incertus* (Ashmead, 1900)**

(Figs 3F, 5F, 7F, 9F, 11F, 13F, 15F)

1900 Ashmead, W.H., *Transactions of the Entomological Society of London* 33:253 Description (♀ ♂) (Comb.: *Sycophila incerta*).

1993 Bouček, Z., *Journal of Natural History* 27: 202, Fig. 37. Lectotype designation. (Comb.: *Idarnes incerta*).

**Type material.** Lectotype. ♀, **USA:** Florida: Coconut Grove (USNM).

Paralectotypes: **ST. VINCENT:** 2♀, Smith HH (USNM). **USA:** **Florida:** Florida city, 1♂, V.1989, Nadel, H, ex *Ficus citrifolia* (BMNH)

**Diagnosis**. Body predominantly yellow orange. Metasoma dorsally brown black, first tergite yellow. Supraclypeal area as wide as torulus. Subantennal groove as long as torulus. Distance from torulus to median ocellus 1× distance from torulus to oral margin. Frenal sulcus smooth. Metascutellum nearly 0.5× as long as frenum to inconspicuous. Postmarginal vein nearly absent, shorter than 1/5× stigmal vein length. Ovipositor sheaths 1.4× as long as hind tibia.

**Female***.*

*Size and colour.* Body length 1.7 mm. Ovipositor length 0.5 mm. Predominantly yellow orange. Vertex brown black near each ocelli. Metasoma dorsally brown black, first tergite yellow.

*Head.* Supraclypeal area as wide as torulus or wider. Subantennal groove as long as torulus. Distance from torulus to median ocellus 1× distance from torulus to oral margin. POL 2.2× OOL. Scape 2.0–2.2× as long as pedicel. Antenna with two anelli. First funicular segment 0.7× as long as wide, with 5–8 multiporous plate sensillae

*Mesosoma.* Mesoscutum reticulate. Mesoscutum and scutellar-axillar complex not strongly curved in lateral view. Notaulus with shallow crenulation. Mesoscutellum 1.4–1.5× as long as wide near transscutal articulation. Axillula longitudinally striate to reticulate. Frenal sulcus smooth. Metascutellum nearly 0.5× as long as frenum to inconspicuous. Anterior margin of propodeum medially angulose (holotype, sometimes concave in other specimens). Propodeal median line traceable at least in the anterior half of propodeum. Stigmal vein as long as marginal vein, with two adstigmal setae. Postmarginal vein nearly absent, shorter than 0.2× stigmal vein length.

*Metasoma.* Ovipositor sheaths 1.4× as long as hind tibia.

**Male**. Similar to female, except sexual characters.

**Host plant**. *Ficus aurea* form *aurea* Nuttal and *Ficus citrifolia* Miller
